# A Flexible Phosphonate Metal–Organic Framework for Enhanced Cooperative Ammonia Capture

**DOI:** 10.1021/jacs.4c12430

**Published:** 2024-11-08

**Authors:** Dukula De Alwis Jayasinghe, Yinlin Chen, Jiangnan Li, Justyna M. Rogacka, Meredydd Kippax−Jones, Wanpeng Lu, Sergei Sapchenko, Jinyue Yang, Sarayute Chansai, Tianze Zhou, Lixia Guo, Yujie Ma, Longzhang Dong, Daniil Polyukhov, Lutong Shan, Yu Han, Danielle Crawshaw, Xiangdi Zeng, Zhaodong Zhu, Lewis Hughes, Mark D. Frogley, Pascal Manuel, Svemir Rudić, Yongqiang Cheng, Christopher Hardacre, Martin Schröder, Sihai Yang

**Affiliations:** † Department of Chemistry, The University of Manchester, Manchester M13 9PL, U.K.; ‡ College of Chemistry and Molecular Engineering, Beijing National Laboratory for Molecular Sciences, Peking University, Beijing 100871, China; § Department of Micro, Nano and Bioprocess Engineering, Faculty of Chemistry Wroclaw University of Science and Technology, Wroclaw 50-370, Poland; ∥ Diamond Light Source, Harwell Science Campus, Oxfordshire OX11 0DE, U.K.; ⊥ Department of Chemical Engineering, The University of Manchester, Manchester M13 9PL, U.K.; # Department of Earth and Environmental Sciences, The University of Manchester, Manchester M13 9PL, U.K.; ¶ ISIS Neutron and Muon Facility, Rutherford Appleton Laboratory, Chilton OX11 0QX, U.K.; ∇ Neutron Scattering Division, Neutron Sciences Directorate, Oak Ridge National Laboratory, Oak Ridge, Tennessee 37831, United States

## Abstract

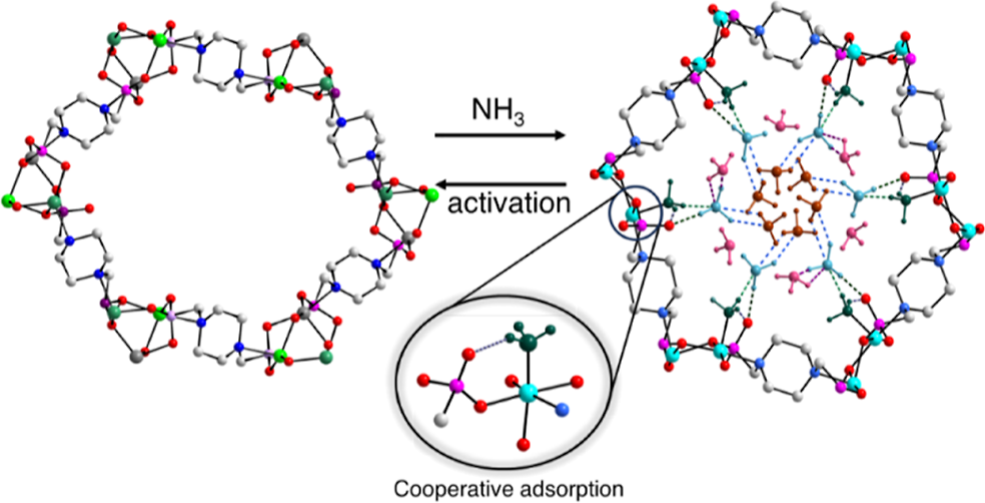

Ammonia (NH_3_) production in 2023 reached 150 million tons and is associated with potential concomitant production of up to 500 million tons of CO_2_ each year. Efforts to produce green NH_3_ are compromised since it is difficult to separate using conventional condensation chillers, but in situ separation with minimal cooling is challenging. While metal–organic framework materials offer some potential, they are often unstable and decompose in the presence of caustic and corrosive NH_3_. Here, we address these challenges by developing a pore-expansion strategy utilizing the flexible phosphonate framework, STA-12(Ni), which shows exceptional stability and capture of NH_3_ at ppm levels at elevated temperatures (100–220 °C) even under humid conditions. A remarkable NH_3_ uptake of 4.76 mmol g^–1^ at 100 μbar (equivalent to 100 ppm) is observed, and in situ neutron powder diffraction, inelastic neutron scattering, and infrared microspectroscopy, coupled with modeling, reveal a pore expansion from triclinic to a rhombohedral structure on cooperative binding of NH_3_ to unsaturated Ni(II) sites and phosphonate groups. STA-12(Ni) can be readily engineered into pellets or monoliths without losing adsorption capacity, underscoring its practical potential.

## Introduction

Global NH_3_ production, crucial for fertilizer and pharmaceutical production and as a hydrogen carrier, is expected to rise due to ever-increasing demand.^1−4^ The Haber–Bosch process operates above 400 °C and at 150 bar and is linked to condensation chillers operating at −25 °C and 140 bar to recycle unreacted N_2_ and H_2_.^5^ It is estimated that 1.87 tons of CO_2_ are emitted per ton of NH_3_ produced, accounting for 1.4–1.8% of global CO_2_ emissions annually.^3^ Efforts to decarbonize NH_3_ production have led to the development of second-generation ruthenium catalysts that operate at 300–400 °C at lower pressures^6,7^ and of greener routes using photo-^8,9^ and electrocatalysts.^10^ However, the separation of NH_3_ via condensation is challenging as NH_3_ is produced at low concentrations.^11,12^ Successful capture and separation of NH_3_ at low concentrations can potentially reduce capital costs by at least 5-fold.^13,14^ Separation beds using metal halides such as MgCl_2_ have been suggested as replacements for chillers as they exhibit high NH_3_ uptake at low NH_3_ partial pressures (0.002–0.1 bar) and show high-temperature operability.^15^ However, they require high energy regeneration and often decompose after a few cycles with decreased NH_3_ uptake efficiency.^16^

**Figure 1:**
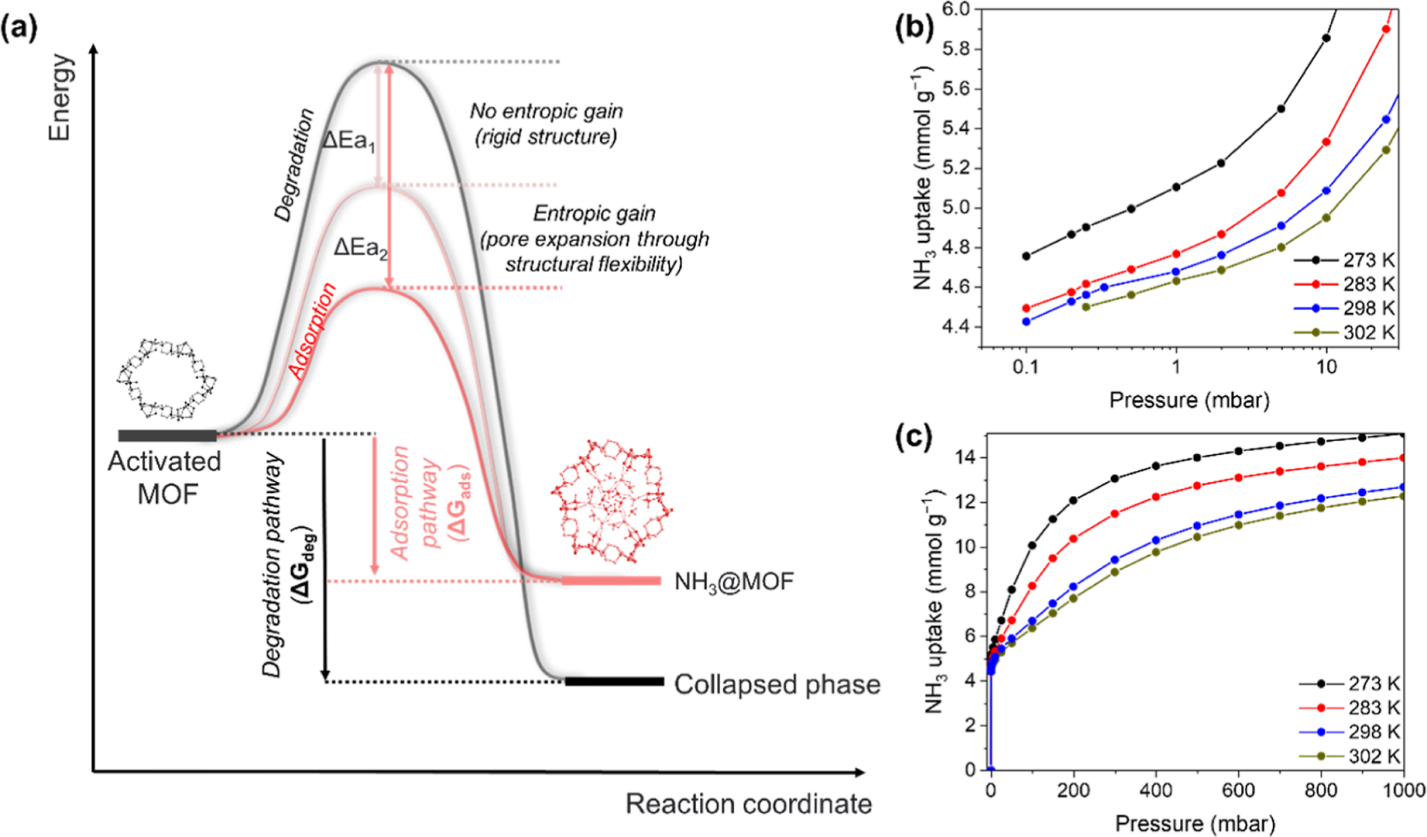
(a) Energy profile diagram for STA-12(Ni) depicting adsorption control using a pore-expansion strategy to favor adsorption over structural degradation (and desorption). Entropic gain facilitates adsorption by reducing the kinetic barrier (Δ*E*a_2_) relative to the barrier for degradation. The activation energy barrier (Δ*E*a_1_) between the adsorption and degradation pathways is smaller, increasing the likelihood of adsorption with structural collapse. (b,c) View of the low-pressure and overall NH_3_ uptake of [Ni_2_(L)] between 273 and 302 K.

Metal–organic framework (MOF) materials have emerged as tunable sorbents due to their high NH_3_ uptake capacities.^4,17−25^ However, sorbents that combine high adsorption and capture efficacy at low pressures, coupled to stability against corrosive and caustic NH_3_ under relevant conditions, such as high temperature and humidity, remain elusive.^26^ To date, sorbents that satisfy the stringent requirements for effective and reliable NH_3_ management in industrial settings remain limited. Herein, we establish a pathway to engineer a robust sorbent for NH_3_ capture using a novel pore-expansion strategy that drives uptake by an increase in the entropy on substrate adsorption (i.e., a positive Δ*S*_ads_^‡^). The chemical stability of MOFs relies on the choice of metal ions and appropriate linkers, utilizing the enthalpy (Δ*H*_ads_^‡^) of metal–ligand binding to drive substrate uptake and avoid host degradation. On substrate uptake, there tends to be a net decrease in entropy (i.e., a negative Δ*S*^‡^_ads_) since polar gaseous adsorbates such as NH_3_ become typically more ordered on adsorption within pores. Our strategy is based around the use of a flexible MOF to give an entropic drive for substrate adsorption through pore expansion, especially at low substrate partial pressure (Figure 1a), thus pivoting the system toward adsorption rather than structural degradation as is typically observed with NH_3_ uptake in MOFs.^4,26^

## Results and Discussion

STA-12(Ni) is a phosphonate-based flexible MOF containing Ni(II) sites bridged by *N*,*N′*-piperazine-bis(methylenephosphonate) linkers (L^4–^).^27,28^ Synthesis in water affords the fully hydrated rhombohedral framework, [Ni_2_(L)(H_2_O)_2_]·6H_2_O, which can be dehydrated (activated) by heating at 100 °C under a dynamic vacuum of 4 × 10^–3^ mbar for 6–8 h to constant weight to give the partially hydrated form of STA-12(Ni), [Ni_2_(L)(H_2_O)_2_], with H_2_O bound to Ni(II). Heating at 170 °C under a dynamic vacuum of 4 × 10^–5^ mbar for 6 h affords a fully dehydrated (activated) form STA-12(Ni)_act_, [Ni_2_(L)], incorporating a narrower triclinic framework.

Upon exposure to NH_3_, STA-12(Ni)_act_ undergoes a transformation to a rhombohedral structure, which induces a distinct stepped isotherm demonstrating a record-high NH_3_ uptake of 4.76 mmol g^–1^ at an ultra-low pressure of 100 μbar (equivalent to 100 ppm) (Figure 1b,c). This is driven by NH_3_ coordination to the Ni(II) sites to give an overall uptake of 15.0 mmol g^–1^ at 1 bar, 273 K (Figure 1c), corresponding to a high storage density of 0.34 g cm^–3^. STA-12(Ni) can maintain its structural integrity through at least 90 adsorption–desorption cycles and remains stable after being immersed in an 18 M NH_3_ solution for 30 h and boiling in the same solution for 2 h at 100 °C (Figure S1). This performance is underpinned by synergy between the Brønsted basic phosphonate moiety of the framework and the vacant metal site at Ni(II) that facilitates the binding of NH_3_ to Ni(II) coupled to hydrogen bonding between NH_3_ and P=O groups. This drives the packing of NH_3_ close to that of solid NH_3_ at 195 K,^29^ as confirmed by neutron powder diffraction (NPD) and inelastic neutron scattering (INS) (see below).

STA-12(Ni)_act_ undergoes a pore expansion on uptake of NH_3_ leading to a net increase in entropy of Δ*S* = +79 J K^–1^ mol^–1^ on going from 4.5 to 7.0 mmol g^–1^ surface coverage (Figure S2). Thus, adsorption is favored over framework degradation and is supported by the enthalpic stability gained through Ni(II)–NH_3_ bonding. The analogue STA-12(Mn), despite possessing the same structure, exhibits reduced stability toward NH_3_ due to the kinetic lability of Mn(II) compared with Ni(II) (Figures S3 and S4). The rigid, isostructural STA-16(Ni) material, incorporating the extended linker *N*,*N′*-4,4′-bipiperidine-bis(methylenephosphonate),^30^ demonstrates lower NH_3_ uptake at low pressures (Figures S3 and S5) suggesting that the remarkable NH_3_ uptake of STA-12(Ni) at 100 μbar is driven not only by entropic gain but also by the narrower pore structure.

### Performance under Dynamic Conditions

**Figure 2:**
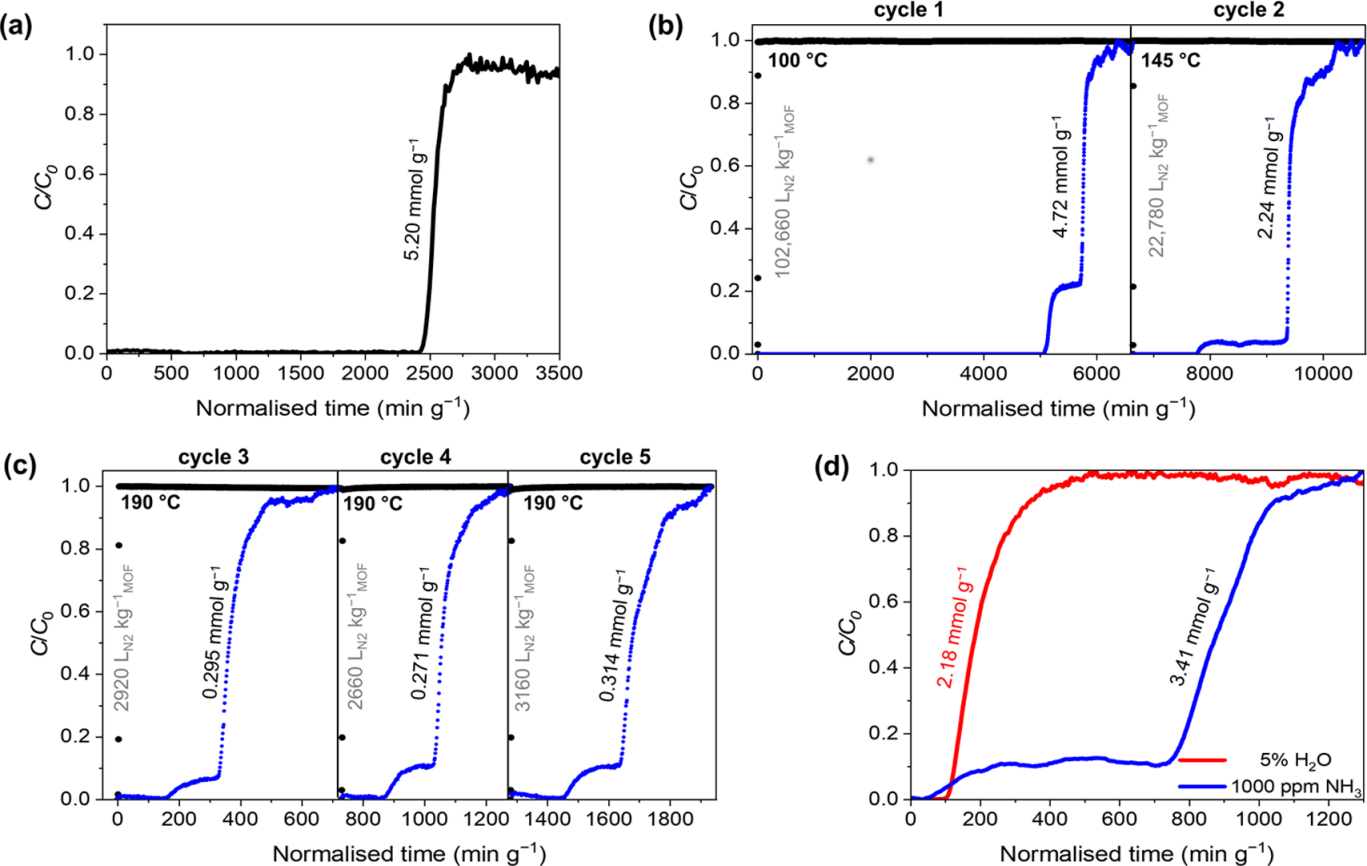
(a) Breakthrough of dry NH_3_ through STA-12(Ni) using a 50 mL flow of 1000 ppm of NH_3_ diluted in He at 298 K. Dry NH_3_ breakthrough cycling experiments under a flow of 20 mL of 1000 ppm of NH_3_ diluted in N_2_ with STA-12(Ni) at (b) 100 and 145 °C (cycle 1 and 2) and (c) 190 °C (cycles 3–5). The N_2_ working capacity in each cycle is shown in gray. (d) NH_3_ and H_2_O breakthrough at 150 °C through STA-12(Ni) under a flow of Ar containing 1000 ppm of NH_3_ (blue) and 5% H_2_O (100 mL/min).

The dynamic uptake of STA-12(Ni)_act_ at 298 K under a flow of 1000 ppm of NH_3_ in He was measured as 5.20 mmol g^–1^ (Figure 2a), consistent with the value obtained from the static uptake measurements and exemplifying its potential application in capturing NH_3_ at low partial pressures. The purity of the gas emitted under flow through STA-12(Ni)_act_ remains less than 6 ppm of NH_3_ until full breakthrough is achieved. This level is lower than the dangerous (300 ppm) and short-term workplace exposure limits (50 ppm) for NH_3_ as set by the National Institute for Occupational Safety and Health.^31^

To evaluate the applicability of STA-12(Ni)_act_ for NH_3_ capture at elevated temperatures, dynamic breakthrough experiments using a fixed-bed column of STA-12(Ni)_act_ under a 1000 ppm flow of dry NH_3_ diluted in N_2_ were undertaken at 100, 145, and 190 °C (Figure 2b,c). The material could be readily recycled throughout these experiments. STA-12(Ni)_act_ successfully captures and separates NH_3_ under these harsh conditions and is comparable to alkaline metal halides.^32,33^ In all cases, a high dynamic uptake corresponding to a distinctive two-step breakthrough was observed, attributed to competition between the different strengths of the NH_3_ binding sites coupled with the structural dynamics of the framework. Crucially, STA-12(Ni)_act_ generates N_2_ in 99.998% purity at the outlet with working capacities of 102,660 and 22,780 L kg^–1^ at 100 and 145 °C, respectively. A high average dynamic uptake for NH_3_ of 0.293 mmol g^–1^ is observed at 190 °C in the cycling experiment. In situ infrared (IR) microspectroscopy using pure NH_3_ and NH_3_ diluted to 1% and 10% in N_2_, conditions replicating the Haber–Bosch process, confirms that the material is capable of adsorbing NH_3_ from 25 to 275 °C (Figure S6). The capture of NH_3_ under humid conditions is another challenging task, with most previous studies focusing on hydrophobic sorbents.^34,35^ Remarkably, STA-12(Ni)_act_ is also capable of capturing NH_3_ effectively from a flow of 1000 ppm of NH_3_ and 5% H_2_O in Ar at 150 °C with a dynamic uptake of 3.41 mmol g^–1^ (Figure 2d). Heating NH_3_-saturated STA-12(Ni) at 190 °C releases all of the captured NH_3_. In an industrial setting, this would result in ca. 0.6 GJ ton^–1^_NH_3__ energy loss.^13^ Under similar conditions, metal halides require heating to 300 °C for full regeneration, with a significant energy penalty of 3.4 GJ ton^–1^_NH_3__.

### Visualization and Mechanism of Host–Guest Binding and Dynamics

**Figure 3:**
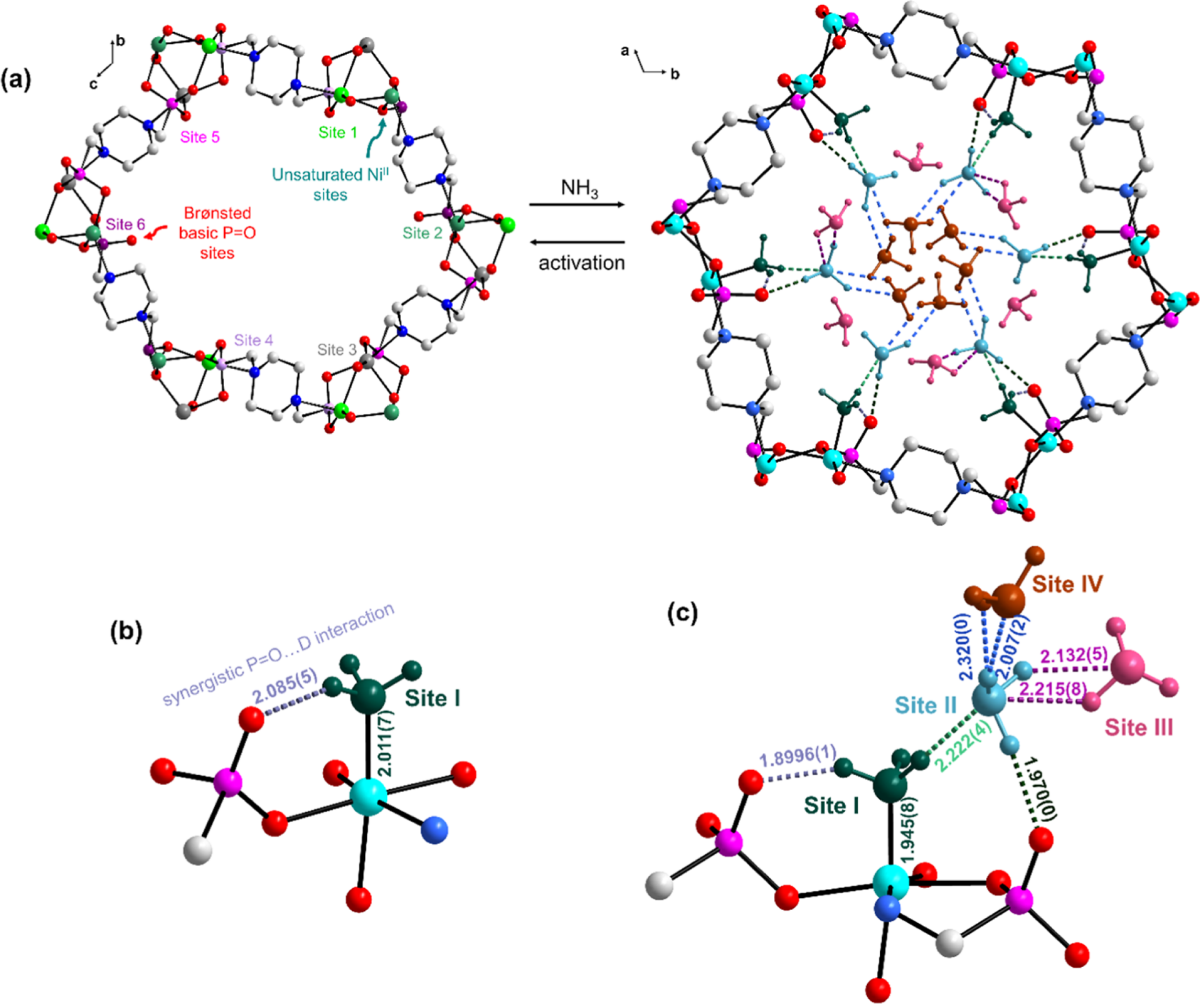
(a) View of structural transformation of STA-12(Ni) from triclinic (left) to rhombohedral (right) symmetry induced on NH_3_ uptake, showing framework sites 1–6. View of binding sites in STA-12(Ni) of (b) low-loaded (0.95 ND_3_/Ni) and (c) high-loaded (1.82 ND_3_/Ni) [Ni_2_(L)] showing sites I–IV for NH_3_ positions. The Ni^II^, N(ligand), C, O, and P atoms are shown in light blue, sea blue, white, red, and purple, respectively. ND_3_ binding sites I to IV are highlighted in dark green, bright blue, cream pink, and maroon, respectively. The synergistic interactions between Brønsted basic P=O groups and Ni-bound chemisorbed NH_3_ are also indicated.

To visualize the binding domains of NH_3_ in STA-12(Ni), in situ NPD data for bare and ND_3_-loaded STA-12(Ni) were collected with ND_3_:Ni loadings of 0.96 (low loading) and 1.82 (high loading). Rietveld refinement of STA-12(Ni)_act_, [Ni_2_(L)], confirmed three crystallographically distinct Ni^II^ sites (sites 1 to 3) and three [C–PO_3_]^2–^ sites (sites 4 to 6) (Figure 3a). Rietveld refinement of data for low loading confirmed the location of chemisorbed ND_3_ molecules at site I (0.96 ND_3_/Ni) with a Ni···ND_3_ distance of 2.022 and a strong synergistic hydrogen bonding interaction P=O···D–N = 2.085 Å (Figure 3b). At high loading, three additional binding sites for NH_3_ (sites II–IV) were revealed (Figure 3c). At site I, with tighter substrate packing, the Ni···ND_3_ and P=O···D–N distances decreased to 1.946 and 1.900 Å, respectively. Physisorbed ND_3_ at site II with an occupancy of 0.67 ND_3_/Ni revealed a further strong hydrogen bonding interaction with the P=O group and with other ND_3_ molecules (P=O···D_3_N = 1.972, ND···ND_3_ = 2.222 Å). Site II further interacts with sites III and IV with ND_3_:Ni occupancies of 0.181 and 0.046, respectively, and hydrogen bonding interactions (site III, ND···ND_3_ = 2.1325; D_3_N···DN = 2.216 Å, site IV, DN···D_3_N = 2.036 Å). These are comparable to distances observed in solid NH_3_ at 7 K (H_3_N···HN_3_ = 2.130 Å)^17^ and confirm the presence of a combination of chemisorbed and physisorbed ND_3_, amplified by strong synergistic interactions (Figure 3a) between the Brønsted basic phosphonate P=O groups and adsorbed NH_3_ molecules. Furthermore, packing of NH_3_ at a packing density of 0.74 g cm^–3^ at 273 K confirms its potential for NH_3_ capture, storage, and transportation, particularly associated with the Haber–Bosch process.

**Figure 4:**
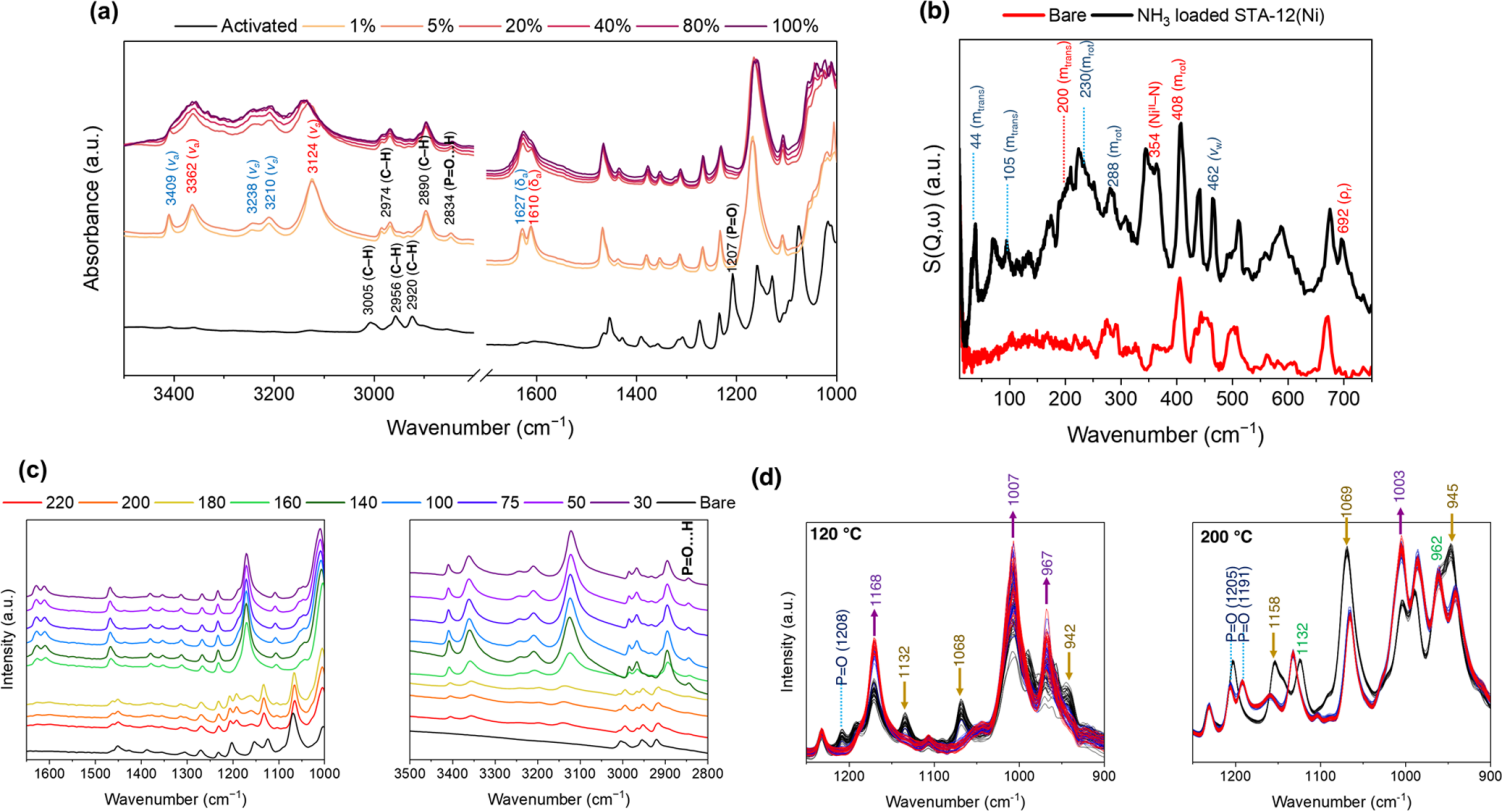
(a) In situ IR spectra of STA-12(Ni), [Ni_2_(L)], with increasing NH_3_ loading and (b) experimental INS spectra of bare (red) and NH_3_-loaded [Ni_2_(L)] (black). In both cases, vibrational and molecular motions associated with chemisorbed and physisorbed NH_3_ are highlighted in red and dark blue, respectively. Bands associated with [C–PO_3_]^2–^ which decrease in intensity are marked in blue, with new bands indicated in purple. (c) IR spectra of [Ni_2_(L)] under a 100 mL flow of 1% NH_3_ diluted in N_2_ from 30 to 220 °C. (d) IR spectra obtained using rapid scans at 120 °C (left) and 200 °C (right) under flow; the color scheme indicates the time at which the spectra were obtained from the point of start of flow. In each case, the P=O group peaks are highlighted in blue. Bands associated with [C–PO_3_]^2–^ perturbations are also indicated; bands expected to decrease and increase are shown in brown and purple, respectively. The bands which did not undergo significant changes are highlighted in green.

The host–guest binding dynamics in this system were investigated further using a combination of in situ INS, density functional theory simulations, and in situ synchrotron IR microspectroscopy. Substantial changes in IR spectra upon loading of NH_3_ into STA-12(Ni)_act_ were observed due to the structural transition that occurs on NH_3_ chemisorption (Figure 4a). Peaks associated with C–H stretching (3005–2920 cm^–1^) convert to two bands centered at 2890 and 2970 cm^–1^ during NH_3_ sorption, suggesting that the piperazine moieties become symmetrically equivalent. This is further confirmed through changes in the P–O vibrational region where bands at 1127, 1074, and 943 cm^–1^ decrease in intensity and peaks at 1158, 1011, and 965 cm^–1^ are generated. This suggests a significant distortion of the [C-PO_3_]^2–^ moieties, related to rotation of the [C-PO_3_]^2–^ tetrahedra at binding sites 4 and 5 during NH_3_ chemisorption and the formation of symmetrically equivalent phosphonate tetrahedra. Bands corresponding to N–H stretches (3409–3124 cm^–1^) and deformations (1627–1610 and 1184–1052 cm^–1^) of physisorbed and chemisorbed NH_3_ appear upon contact with NH_3_. The rocking (ρ_r_) and stretching modes of Ni–NH_3_ are observed at 683 and 354 cm^–1^, respectively. IR bands associated with chemisorbed NH_3_ shift and broaden with increasing NH_3_ loading (Figure S7), consistent with the formation of hydrogen bonding between sites I and II. The presence of the symmetric N–H stretch of physisorbed NH_3_ suggests the formation of NH_3_ clusters facilitated by extensive hydrogen bonding interactions.^36,37^ Interestingly, the band at 1207 cm^–1^ assigned to the P=O stretching mode weakens in intensity and red-shifts by ca. 18 cm^–1^ upon NH_3_ loading due to weakening of the π bond of P=O via strong hydrogen bonding interactions. The appearance of the weak peak at 2834 cm^–1^ further suggests the formation of an intramolecular P–O···H interaction.^38^

The INS spectra (Figure 4b) reveal the molecular-level behavior of adsorbed NH_3_. Rotational motion (*m*_rot_) of chemisorbed and physisorbed NH_3_ was observed at 408 and 288–230 cm^–1^, and translational motion (*m*_trans_) was observed at 200 and 105–53 cm^–1^. This reflects the host-guest interactions of the NH_3_ molecules with the framework. Compared to the INS spectra of solid NH_3_,^17^ bands associated with physisorbed NH_3_ are blue-shifted and broadened, consistent with hydrogen bonding between NH_3_ molecules and the framework. The increase in intensity of existing peaks associated with the framework dynamics upon NH_3_ loading confirms responsiveness of the material toward NH_3_.

IR spectroscopic measurements using a flow of 1% NH_3_ diluted in N_2_ (100 mL/min) between 25 and 220 °C confirmed the presence of both chemisorbed and physisorbed NH_3_, with the intensities of the bands associated with the latter decreasing with an increase of temperature (Figure 4c). To investigate further the dynamics and adsorption mechanisms at higher temperatures, we undertook rapid IR measurements under flow at 120 and 200 °C. Significant differences were observed in the spectra at 200 °C compared with those at 120 °C. At 200 °C, instead of an increase, the band at 1158 cm^–1^ attributed to the [C-PO_3_]^2–^ moiety blue-shifts and decreases in intensity, while the band at 1125 cm^–1^ is blue-shifted to 1132 cm^–1^ but does not show a notable decrease (Figure 4d). The split and red shift of the band assigned to the P=O stretching mode is also clearly observed in these measurements; at 120 °C, the band initially at 1208 cm^–1^ slowly disappears, while a band at 1191 cm^–1^ grows, which then overlaps with the intense band at 1168 cm^–1^. At 200 °C, these bands are clearer (1205 and 1191 cm^–1^) since the intensity of the band at 1158 cm^–1^ associated with the [C-PO_3_]^2–^ unit is less intense. The band at 2834 cm^–1^ assigned to the P–O···H interaction also appears under these conditions (Figure 4c), becoming sharper at lower temperature, further illustrating the interplay of the Brønsted basic P=O sites in facilitating strong hydrogen bonding with NH_3_. This indicates that adsorbed NH_3_ is strongly immobilized with limited motion, consistent with INS data. These observations also suggest at elevated temperatures that only the accessible [C-PO_3_]^2–^ units rotate, and an intermediate between triclinic and rhombohedral symmetry exists upon NH_3_ adsorption, possibly linked to partial coordination to Ni(II) sites within the structure. This also confirms that the driving force for NH_3_ capture at high temperatures is linked to the associated increase in the entropy during adsorption. Computational modeling of the NH_3_ isotherms further supports this hypothesis (Figure S8), which confirms a higher uptake for the rhombohedral framework with more accessible Ni(II) sites at lower pressure than the triclinic structure.

### Scalable Material Synthesis and Engineering

We have also developed a microwave synthesis for STA-12(Ni) to give the material in greater than 75% yield (based on linker) in 3 min, a striking improvement over the published method (Figure S9).^27,28^ It can also be prepared under reflux at 160 °C for 6 h (Figure S9), suggesting a route to a cost-effective scale-up of this material.^39^

**Figure 5:**
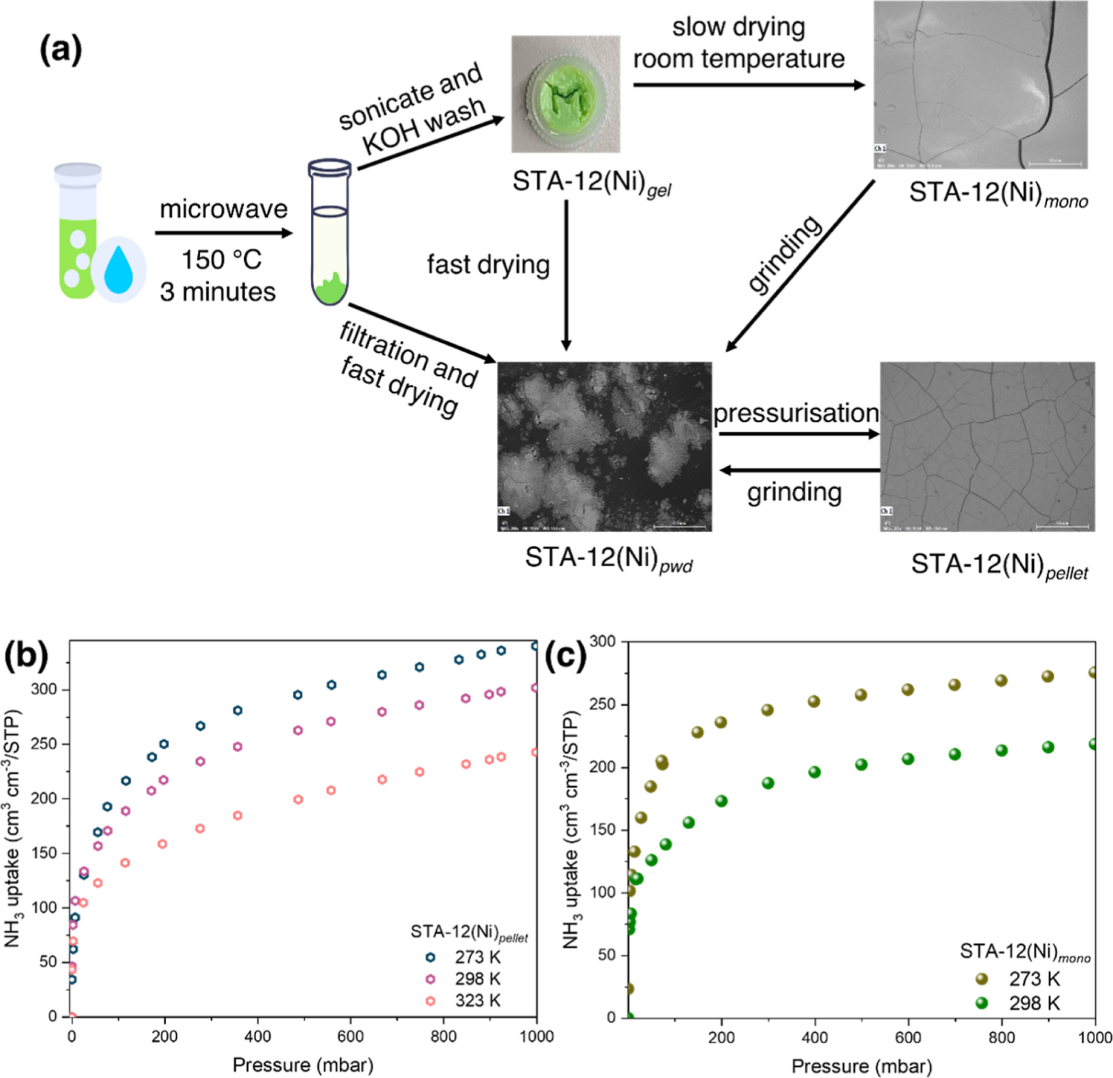
(a) Schematic of synthesis of powdered, gel, pelletized, and xerogel-derived monolithic forms of STA-12(Ni). Volumetric NH_3_ uptakes of (b) fully activated [Ni_2_(L)]_pellet_ and (c) partially activated [Ni_2_(L)(H_2_O)_2_]_mono_.

Evaluation of sorbents is often based on their powdered forms and theoretical crystallographic framework densities. However, these theoretical values significantly differ from the practical densities. For example, the tapped density of STA-12(Ni) in powdered form, STA-12(Ni)_pwd_, is ca. 0.057 g cm^–3^ (Figure S10), nearly 26 times less than its crystallographic density. Addressing this discrepancy, the compression of MOF powders into pellets to enhance packing efficiency and volumetric gas storage density is attractive, but this can potentially lead to mechanical collapse of the material resulting in lower uptakes.^40,41^ Recent work has also highlighted monolithic forms of MOFs which can avoid such mechanical collapse.^42−44^ We assessed the practicality of STA-12(Ni)_act_ for sorption by processing it into pellets under 3 tonnes pressure (Figure 5a). STA-12(Ni)_pellet_ shows a density of 1.14 g cm^–3^ with an overall gravimetric uptake at 1 bar for NH_3_ only slightly reduced by 0.2 mmol g^–1^ compared to its powder form STA-12(Ni)_pwd_ at 323 K. This represents only a 2% reduction in capacity (Figure S11). However, STA-12(Ni)_pellet_ shows a volumetric NH_3_ uptake of 289 cm^3^ cm^–3^/STP at 1 bar, 298 K (Figure 5b). This is significantly higher than the volumetric uptake of 15.3 cm^3^ cm^–3^/STP under the same conditions for fully activated STA-12(Ni)_pwd_. This confirms the efficacy of pelletization for improved volumetric uptake capacities without compromising gravimetric uptake.

A xerogel-based monolithic form of STA-12(Ni) was prepared using a base-induced gelation method followed by slow-drying at room temperature (Figures 5a and S12–S19). Interestingly, the moldability of precursor STA-12(Ni)_gel_ before drying offers flexibility in shaping the material to fit specific storage containers, presenting a potential higher packing efficiency than both pellet and powder forms. Drying of the gel form of STA-12(Ni)_gel_ afforded the first example of monolithic phosphonate-based MOF STA-12(Ni)_mono_ incorporating [Ni_2_(L)(H_2_O)_2_] with H_2_O still bound to Ni(II). The fully dehydrated, activated form of the monolith could be prepared but collapses in air and in the presence of NH_3_ presumably due to the bulk material undergoing structural transformation on coordination of NH_3_ to Ni(II). A stable monolithic form could be generated by heating the sol–gel form at 100 °C under a dynamic vacuum of 4 × 10^–3^ mbar for 6–8 h to constant weight such that only physisorbed H_2_O in the pore is removed, to yield STA-12(Ni)_mono_, [Ni_2_(L)(H_2_O)_2_]. STA-12(Ni)_mono_ shows a density of 1.25 g cm^–3^ (Figure 5c) with a volumetric NH_3_ uptake of 202 cm^3^ cm^–3^/STP at 1 bar, 298 K. We thus sought to compare the partially hydrated form of STA-12(Ni)_pwd_ with all physisorbed H_2_O molecules removed but with coordinated water still present within STA-12(Ni)_mono_. At low pressure (<5 mbar NH_3_), partially hydrated STA-12(Ni)_pwd_ and STA-12(Ni)_mono_ changed color to blue green suggesting NH_3_ coordination to Ni(II) sites within the sample. STA-12(Ni)_mono_ shows an NH_3_ uptake of 202 cm^3^ cm^–3^/STP at 1 bar, 298 K (as described above), and partially hydrated STA-12(Ni)_pwd_ has a tapped density of 0.063 g cm^–3^ and a much lower volumetric NH_3_ uptake of 12.4 cm^3^ cm^–3^/STP confirming that monolith formation affords a significant 16-fold improvement in NH_3_ uptake. The NH_3_-loaded materials can be fully rehydrated by washing with water. This is particularly useful in NH_3_ capture during wastewater treatments, and this exchange of NH_3_ under flow conditions was confirmed further using in situ synchrotron IR spectroscopy (Figure S20).

## Conclusions

We confirm the use of STA-12(Ni) as a sorbent that meets the criteria for practical NH_3_ capture and storage by demonstrating adaptability and stability under appropriate conditions. STA-12(Ni)_act_ efficiently captures NH_3_ at low concentrations and maintains efficacy across a wide temperature range (25–220 °C), marking a significant advance over existing sorbents. Its resilience, evidenced by sustained structural integrity even after rigorous NH_3_ cycling and exposure to harsh environments including pure NH_3_, is attributed to the increase in entropy due to structural change on substrate uptake, and the cooperative interaction between vacant metal sites and Brønsted basic phosphonate P=O groups to enhance binding and hydrogen bonding of trapped NH_3_. The excellent performance of STA-12(Ni), particularly in pellet and monolithic forms, underscores its potential as a scalable and efficient solution for industrial NH_3_ management challenges.

## Supporting Information Available

Synthesis, characterization, and analysis of NH_3_-loaded materials and neutron diffraction, INS, modeling, and synchrotron IR microspectroscopy data (PDF)
