# Supplementary material for: A Flexible Phosphonate Metal–Organic Framework for Enhanced Cooperative Ammonia Capture
Source: J Am Chem Soc. 2024 Nov 8;146(46):32040–8. doi: 10.1021/jacs.4c12430 (PMC11583364; doi:10.1021/jacs.4c12430)
Supplement: Supplementary file 1 — ja4c12430_si_001.pdf [file ja4c12430_si_001.pdf]

Supplementary Materials

A Flexible Phosphonate Metal Organic Framework for Enhanced Cooperative Ammonia Capture

Dukula De Alwis Jayasinghe,[1] Yinlin Chen,[1] Jiangnan Li,[1,2] Justyna M. Rogacka,[1,3] Meredydd Kippax–Jones,[1,4] Wanpeng Lu,[1] Sergei Sapchenko,[1] Jinyue Yang,[1] Sarayute Chansai,[5] Tianze Zhou,[1] Lixia Guo,[2] Yujie Ma,[1] Longzhang Dong,[1] Daniil Polyukhov,[1] Lutong Shan,[1] Yu Han,[1] Danielle Crawshaw,[1] Xiangdi Zeng,[1] Zhaodong Zhu,[1] Lewis Hughes,[6] Mark D. Frogley,[4] Pascal Manuel,[7] Svemir Rudić,[7] Yongqiang Cheng,[8] Christopher Hardacre,[5] Martin Schröder[1]* and Sihai Yang[1,2]*

1. Department of Chemistry, The University of Manchester, Manchester, M13 9PL, UK. M.Schroder@manchester.ac.uk; Sihai.Yang@manchester.ac.uk
2. College of Chemistry and Molecular Engineering, Beijing National Laboratory for Molecular Sciences, Peking University, Beijing 100871, China. Sihai.Yang@pku.edu.cn
3. Department of Micro, Nano and Bioprocess Engineering, Faculty of Chemistry Wroclaw University of Science and Technology, Wroclaw, Poland.
4. Diamond Light Source, Harwell Science Campus, Oxfordshire, OX11 0DE, UK.
5. Department of Chemical Engineering, The University of Manchester, Manchester, M13 9PL, UK.
6. Department of Earth and Environmental Sciences, The University of Manchester, Manchester, M13 9PL, UK.
7. ISIS Neutron and Muon Facility, Rutherford Appleton Laboratory, Chilton, OX11 0QX, UK.
8. Neutron Scattering Division, Neutron Sciences Directorate, Oak Ridge National Laboratory, Oak Ridge, Tennessee 37831, United States.

TABLE OF CONTENTS

Synthetic procedures	S3
Materials engineering and processing	S4
Gas sorption measurements	S5
Breakthrough and temperature programmed desorption	S5
In situ neutron powder diffraction	S5
Inelastic neutron scattering	S6
In situ synchrotron IR spectroscopy	S8
Density functional theory calculations	S8
Molecular simulations	S9
Adsorption density calculations	S9
Supplementary text	S10
Supplementary Figures	S12
Supplementary Tables	S63
Supplementary References	S68

Synthetic procedures

All chemicals were purchased from Alfa Aesar, Sigma Aldrich or ACROS Organics and used as provided without further purification. Ultrahigh purity-grade (99.999 – 99.98) N_2, CO_2 and NH_3 used were purchased from BOC, UK. ^{1}H and ^{31}P Nuclear Magnetic Resonance (NMR) spectra were recorded on a B500 Bruker Advance II+ spectrometer, and thermal gravimetric analyses (TGA) carried out under air flow (50 mL min^{-1}) at a heating rate of 3 °C min^{-1} using a TA SDT-600 thermogravimetric analyser. Elemental analyses were performed on a Thermo iCap 6300 inductively coupled plasma emission spectrometer (ICP-OES) and a CE-440 elemental analyser (EAI company). Powder X-ray diffraction (PXRD) data were recorded on a Philips X'pert X-ray diffractometer (45 kV and 30 mA) using Cu-Kα radiation (λ = 1.5406 Å). Scanning electron microscopy (SEM) was performed using an FEI/Thermofisher Quanta 650 FEG-(E)SEM equipped with a Bruker X Flash 6-30 silicon drift detector.

Synthesis of *N,N'* -piperazine-bis(methylenephosphonic acid)

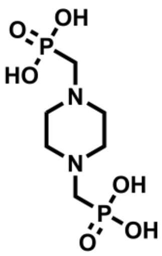

N,N'-Piperazine-bis(methylenephosphonic acid) was synthesised *via* a modified Mannich reaction. In a typical synthesis, piperazine (7.83 g, 0.09 mol) was dissolved in distilled water (75 mL) and phosphoric acid (20.0 g, 0.23 mol), and hydrobromic acid (18 *wt.*%, 100 mL) was added. Formaldehyde (35 *wt.*%, 40 mL) was then added dropwise over 45 mins with stirring, and the resulting mixture refluxed for 20 h. The mixture was then cooled down to 2 °C for 10 h to ensure complete precipitation. The white solid was collected by vacuum filtration, washed with copious amounts of distilled water, and cold EtOH, then dried at 60 °C for 12 h. 21.2 g, 86% yield. ^{1}H NMR (500 MHz, D_2O/NaOH, 298 K) δ 2.35 (br, 8H), 2.26 (d, J = 12.3 Hz, 4H_B). ^{31}P NMR (162 MHz, D_2O) δ 14.45 (*t*, J = 13.0 Hz, *P* - *H*).

Synthesis of *N,N'*-4,4'-bipiperidine-bis(methylenephosphonic acid)

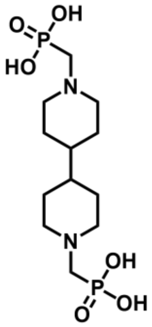

The same procedure as above was carried out but using 4,4'-bipiperidine dihydrochloride (10.9 g, 0.018 mol). 28.0 g, 91% yield. ^{1}H NMR (500 MHz, D_2O/NaOH, 298 K) δ 2.85 (*d*, J = 11.3 Hz, 4H), 2.27 (*d*, J = 12.1 Hz, 4H), 2.03 – 1.88 (m, 4H), 1.50 (*d*, J = 12.7 Hz, 4H), 1.09 – 0.78 (m, 6H). ^{31}P NMR (162 MHz, D_2O/NaOH) δ 15.13 (*t*, J = 12.1 Hz, P - H).

Synthesis of MOF materials

STA-12(Ni), STA-16(Ni) and STA-12(Mn) were prepared according to the general method previously published.(*1*-*3*) For STA-12(Ni), the synthesis was modified. In a typical synthesis, 157 mg of *N,N'* - piperazine-bis(methylenephosphonic acid) and 209 mg of $Ni(OAc)_2$ were added to water (15 mL). At

this point the synthesis could be carried out using microwave or conventional heating. For the former the solution was placed in a 35 mL capacity microwavable tube. Heating was programmed on a My Discover 2.0 Microwave to pre-stirring for 2 mins, and ramping to 150 °C with a maximum pressure of 240 psi and power of 120 W for 3 mins. The resulting solid was isolated *via* vacuum filtration, washed with distilled water, and oven dried at 75 °C for 20 h. Alternatively, the solution above was placed in a 50 mL round bottom flask and heated at 160 °C under reflux for 6 h. In each case, a yield of *ca.* 70–75% based on the linker was obtained. Phase purity of samples was confirmed through PXRD (**Fig. S9**) and elemental analysis.

Materials engineering and processing

STA-12(Ni)$_{gel}$ and STA-12(Ni)$_{mono}$

MOF gels and related architectures such as monoliths have been engineered through use of functionalised linker precursors, binders, freeze drying (typically used for aerogels) and through controlled nucleation methods.(*4,5*) Solidification of sol-gels can form MOF superstructures, which are usually referred to as monoliths.(*6*) Generally, addition of secondary binders to form MOF-gels may reduce the internal porosity and effect the composition of the MOF material. Thus, attention has led in recent years to the development of binder-free MOF gels and monoliths. However, the underlying chemistry for such gelation is still poorly understood.

In STA-12(Ni), the surface of the material consists of the Brønsted basic P=O groups and we proposed that these groups may aid cross-linking of crystallites of STA-12(Ni)$_{pwd}$ and promote gelation. However, centrifugation and slow drying of the collected material can lead to disaggregation into a powder on application of mechanical force. This suggested that the interaction between the crystallites forming the gel was not sufficient to stabilise the overall bulk structure. We then sought a method to try improving the interactions further; looking into ceramic materials and metal oxides,(*7*) we realized that the use of an appropriate base solution may allow further cross-linking between the crystallites. Hence, we then centrifuged the obtained powder using a *ca.* 1 M KOH solution, at a rate of 10,000 rpm, followed by several washings with distilled water. Intriguingly, the solid became more compacted than before; slow drying of the obtained material and analysing it using PXRD confirmed the structure to be STA-12(Ni). Elemental analysis **(Table S2**) and SEM images (**Figs. S16-S18**) confirmed the presence of K^+ ions, suggesting that they play an important role in the formation of STA-12(Ni)$_{mono.}$

STA-12(Ni)$_{pellet}$

To convert the powder STA-12(Ni)$_{pwd}$ into pellets of STA-12(Ni)$_{pellet}$, 60–80 mg of finely ground STA-12(Ni)$_{pwd}$ was placed in a holder of 8 mm diameter and this was pressed under 3 tonnes (46 MPa) for 5 mins.

Gas sorption measurements

Static NH_3 adsorption isotherms (0-1.0 bar) were measured using an Intelligent Gravimetric Sorption Analyser (IGA) (Hiden™ Isochema, Warrington, UK). As-synthesised samples were activated completely prior to adsorption measurements under dynamic vacuum (4×10^{-5} mbar) at 170 °C for 6 h. For experiments involving $[Ni_2(L)(H_2O)_2]$, the as-synthesised material was activated at 100 °C under a dynamic vacuum of 4×10^{-3} mbar for up to 8 h to constant weight. The isosteric enthalpy of adsorption (ΔH_n) and change in entropy during adsorption (ΔS_n) for NH_3 uptake as a function of gas loading (n) obtained from isotherms run at 273, 283 and 298 K were fitted to the Van't Hoff isochore. The isotherms were fitted to a dual-site Langmuir isotherm model, and a plot of $\ln(p)_n$ *vs.* 1/T at constant loading allowed the differential enthalpy and entropy of adsorption to be determined.

$$\ln(p)_n = \frac{\Delta H_n}{RT} - \frac{\Delta S_n}{R}$$

Breakthrough and temperature programmed desorption

Breakthrough experiments of dry NH_3 were carried out using a fixed-bed flow reactor consisting of a tubular quartz reactor (ID 4 mm). Prior to measurement, the sample was activated at 483 K under dynamic vacuum overnight and *ca.* 60 mg of activated sample (of 250-425 μm thickness) was packed and held in place between plugs of quartz wool. A K-type thermocouple was placed in the centre of the bed. The MOF sample was further treated at 483 K under an Ar flow for 3 h to ensure complete activation before cooling down to 298 K. The breakthrough experiment was performed with a stream of 1000 ppm NH_3 diluted in Ar, at a flow rate of 50 mL min^{-1}. A Hiden™ HPR-20 quadrupole mass spectrometer equipped with a heated quartz inlet capillary was used to analyse the outlet gas for detection of NH_3 (m/z = 17) and Kr (m/z = 84). The gas concentration, C, of NH_3 at the outlet was compared with the corresponding inlet concentration C_0, where C/C_0 = 1.0 indicates complete breakthrough.

For breakthrough studies of wet NH_3 *ca.* 66 mg of an activated sample was packed into a fixed-bed reactor and the breakthrough experiment performed with a stream of 1000 ppm NH_3 and 5% H_2O diluted in Ar, at a flow rate of 100 mL min^{-1}. The outlet stream was analysed using mass spectrometry.

For temperature programmed desorption (TPD) a stream of NH_3 diluted in He was passed through the activated sample using a heating rate of 5 °C min^{-1} from 25 °C to 230 °C.

***In situ* neutron powder diffraction**

Neutron powder diffraction (NPD) experiments for ND_3-loaded STA-12(Ni)$_{act}$ were undertaken on the WISH diffractometer at the ISIS Facility at Rutherford Appleton Laboratory (UK).(*8*) The instrument has a solid methane moderator providing a high flux of cold neutrons with a large bandwidth, transported to the sample via an elliptical guide. The divergence jaws of the WISH system allow tuning of the resolution according to the need of the experiment; in this case, it was set up in high resolution mode. The WISH detectors are 1 m long, 8 mm diameter pixelated 3He tubes positioned at 2.2 m from

the sample and arranged on a cylindrical locus covering a 2θ scattering angle of 10–170°. To reduce the background from the sample environment, WISH was equipped with an oscillating radial collimator that defines a cylinder of radius of approximately 22 mm diameter at 90° scattering angle. STA-12(Ni)$_{act}$, [Ni_2(L)], was loaded into a cylindrical vanadium sample container with an indium vacuum seal connected to a gas handling system. The sample was degassed at 100 °C at 1×10^{-8} mbar to remove any remaining trace of guest molecules. The sample was dosed with ND_3 volumetrically after being warmed to room temperature (298 K) to ensure that the gas was well dispersed and absorbed in the MOF. The amount of ND_3 added was controlled by the pressure difference of the container with known volume and then dosed into the vanadium holder containing [Ni_2(L)]. Data collection for STA-12(Ni)$_{act}$ and two subsequent loadings of ND_3 (0.96 and 1.82 ND_3 molecules per metal site) were performed with the temperature controlled using a He cryostat (7 ± 0.2 K). In the low loading experiment, slightly insufficient ND_3 was dosed into the packed sample resulting in some remaining STA-12(Ni)$_{act}$, as reflected in the obtained NPD pattern. Thus, the refinement shows a 0.96 occupancy of ND_3, which ideally, should have had an occupancy of 1. Structural refinements were carried out using Topas Academic v6.0.(*9*) Background, cell parameters and peak profile underwent Pawley refinement and then transferred to Rietveld refinement for all five banks. Stephens fitting was applied to describe the shape of diffraction peaks and their anisotropic broadening.(*10*) The initial atomic positions were obtained from the published structures with appropriate modification of the number of hydrogen atoms and the length of the hydrogen bonding. Due to the complexity of the initial structure (under P-1 space group and 45 atoms in the asymmetric unit), a local rigid model was applied on all methylene groups (nine in total). In the final stages of the refinement of the bare MOF, the rigid body was replaced by soft restraints on C–H bond length and <H–C–H. The position of the primary ND_3 molecule for the ND_3-loaded MOF was extracted from the difference Fourier map. The positions of other ND_3 molecules were determined from the simulated annealing method provided by TOPAS software, using a rigid body with fixed bond lengths and angles from DFT-optimised molecule geometry. Isotropic displacement parameters (U_{iso}) were used for all atoms including hydrogen and deuterium. The refined structural parameters include the fractional coordinates (*x, y, z*) and isotropic displacement factors (U_{iso}) for all atoms, and the site occupancy factors (SOF) for guest molecules. The final stage of the Rietveld refinement involved only soft restraints to the C–H and N–D bond lengths on the ligand. The quality of the Rietveld refinements was assured with low weighted profile factors.

Inelastic neutron scattering

INS spectra were recorded at TOSCA neutron spectrometer at the ISIS Facility at the Rutherford Appleton Laboratory (UK). Approximately 1 g of STA-12(Ni)$_{act}$ was loaded into cylindrical vanadium sample cells in a glove box, sealed with an indium seal and degassed at 373 K under dynamic vacuum to remove any trace guest molecules. The temperature during data collection was controlled using a He cryostat (7 ± 0.2 K). The loading of NH_3 was performed volumetrically at room temperature, and the

background spectrum of STA-12(Ni)*act* was subtracted from that of NH_3-loaded STA-12(Ni)*act* to obtain the difference spectra.

INS was used to study the binding interaction and structure dynamics in this case because it has several unique advantages:

- INS spectroscopy is ultra-sensitive to the vibrations of hydrogen atoms, and hydrogen is ten times more visible than other elements due to its high neutron cross–section.
- The technique is not subject to any optical selection rules. All vibrations are active and, in principle, measurable.
- INS observations are not restricted to the centre of the Brillouin zone (gamma point) as is the case for optical techniques.
- INS spectra can be readily and accurately modelled. Intensities are proportional to the concentration of elements in the sample and their cross-sections, and the measured INS intensities relate directly to the associated displacements of the scattering atom. Treatment of background correction is also straightforward.
- Neutrons penetrate deeply into materials and pass readily through the walls of metal containers making neutrons ideal to measure bulk properties of this material.
- INS spectrometers cover the whole range of the molecular vibrational spectrum, 0–500 meV (0–4000 cm^{-1}).
- Calculation of the INS spectra by DFT vibrational analysis can be readily achieved, and DFT calculations relate directly to the INS spectra. In the case of solid-state calculations, there are no approximations other than the use of DFT eigenvectors and eigenvalues to determine the spectral intensities.

In situ synchrotron infrared microspectroscopy

In situ synchrotron infrared (IR) microspectroscopy experiments were carried out at the multimode infrared imaging and micro spectroscopy (MIRIAM) beamline at the Diamond Light Source, UK. Measurements were performed using a Bruker Vertex 80 V FTIR equipped with a mid-infrared LN_2-cooled MCT (Mercury Cadmium Telluride) detector and the Diamond Light Source synchrotron as an IR source. Spectra were collected in the range 4000 - 600 cm^{-1} and aperture size at the sample of approximately 20 × 20 μm at 36× magnification. A microcrystalline powder of as-synthesised STA-12(Ni) was scattered onto a 0.5 mm thick ZnSe infrared window and placed within a novel, custom made microreactor gas-tight sample cell equipped with 0.5 mm thick ZnSe windows, a heating stage and gas inlet and outlet. Ultra-pure N_2 and anhydrous NH_3 were dosed volumetrically into the sample cell using mass flow controllers, and the total flow rate was maintained at 100 mL min^{-1} for all experiments. The exhaust from the cell was directly vented to an extraction system and the total pressure in the cell was therefore approximately 1.0 bar for all experiments. For the room temperature experiment, the sample was desolvated under a flow of dry N_2 at 100 mL min^{-1} at 443 K for 30 mins, and cooled to 298 K under a continuous flow of N_2. Dry NH_3 was then dosed as a function of partial pressure, maintaining a total flow of 100 mL min^{-1} with N_2 as the balance gas. The MOF sample was regenerated with a flow of dry N_2. For the partially hydrated experiment, the sample was desolvated under the same flow rate at 343 K for 30 mins and then cooled to room temperature before dosing with NH_3. For higher temperature experiments, the sample was cooled to the required temperature upon activation. Rapid scans were undertaken in some experiments; these were conducted by reducing the number of scans per measurement (*i.e.*, reduced from 256 to only 16 scans, approx 2 secs per measurement) and measurements were undertaken as soon as NH_3 was flowed into the cell. Upon completion, a background measurement was taken and removed from the previously collected spectra to remove any bands associated with gas phase NH_3.

Density functional theory calculations

Spin-polarized density functional theory (DFT) calculations on STA-12(Ni)$_{act}$ and NH_3-loaded STA-12(Ni)$_{act}$ were performed using the Vienna Ab initio Simulation Package (VASP).(*11*) The calculation used the Projector Augmented Wave (PAW) method (*12*, *13*) to describe the effects of core electrons, and Perdew-Burke-Ernzerhof (PBE) implementation of the Generalized Gradient Approximation (GGA) for the exchange-correlation functional.(*14*) Energy cutoff was 800 eV for the plane-wave basis of the valence electrons. The lattice parameters and atomic coordinates solved in this work were used as the initial structure. The total energy tolerance for electronic energy minimization was 10-8 eV. The maximum interatomic force after relaxation was below 0.001 eV/Å. The optB86b-vdW functional(*15*) for dispersion corrections was applied, and a Hubbard U term of 6.2 eV(*16*) was applied to account for the localized 3d orbitals of Ni. The interatomic force constants were calculated by VASP using finite displacement method, and the vibrational eigen-frequencies and modes were then calculated using

Phonopy.(*17*) The OCLIMAX software was used to convert the DFT-calculated phonon results to the simulated INS spectra.(*18*)

Molecular simulations

All simulations of adsorption were performed using Grand Canonical Monte Carlo (GCMC) method. The simulations were conducted at 298 K, in a wide range of pressures (1 mbar-1200 mbar). Simulations were performed using the state-of-the-art RASPA simulation code.(*19*) To describe the gas-gas interactions for NH_3, the forcefield described by Herrera *et al*. was used.(*20*) The interactions within the two STA-12(Ni) structures (*i.e.,* the triclinic and rhombohedral forms before and after pore opening) were modelled using a hybrid approach: (i) the UFF (Universal Force Field) was used for the metal clusters, and (ii) the Dreiding forcefield was applied for the organic linkers.(*21, 22*) The charges on the atoms within the MOFs were calculated using the PACMOF machine learning script.(*23*) The Lennard-Jones potential was used to describe gas-sorbent interactions, an approach which is widely accepted for simulating van der Waals (VdW) forces.(*24-28*) The Lorentz-Berthelot mixing rules were applied to calculate the interactions between different types of atoms. The VdW interactions were cut-off at the distance of 12 Å, and electrostatic interactions were calculated using the Ewald summation technique, which is computationally efficient for systems with periodic boundary conditions. The implemented Monte Carlo moves consisted of translation, rotation, insertion/deletion of the molecules, and swap moves. The production runs consisted of a minimum of 50,000 cycles to ensure system equilibration, and the radial distribution function (RDF) between nitrogen atoms was calculated during the production cycles.

Adsorption density calculations

Methods of determination of pore volume include using, most commonly, N_2 or CO_2 isotherm measurements or alternatively free volume calculations using computational software such as Mercury(*29*) and PLATON SQUEEZE.(*30*) For STA-12(Ni), the pore volume calculated from the N_2 isotherm at 77 K is for the smaller pores of the activated triclinic material, as N_2 does not trigger the structural transition. Thus, this value does not accurately represent the pore volume of the rhombohedral framework. Therefore, we used crystallographic information file (cif) of the ND_3 loaded STA-12 material (ND_3/Ni = 1.82) as a basis. Chemisorbed and physisorbed ND_3 molecules were removed using OLEX2 (v. 1.5),(*31*) and the free volume in the material was determined using Mercury with a probe radius of 1.2 Å and approximate grid spacing of 0.1 Å. This gave a free volume of 45.5%, with a density of 1.348 g cm^{-3}, and from this a pore volume of 0.338 g cm^{-3} was calculated. The NH_3 packing density was then determined by:

Packing density = amount of NH_3 adsorbed (g g^{-1})/pore volume (cm^3 g^{-1})

The theoretical storage density of a material can be calculated by:

Storage density = amount of NH_3 adsorbed (g g^{-1}) × density of material (g cm^{-3})
In order to determine the theoretical storage density, we used the crystallographic density obtained from the cif file. The *theoretical* NH_3 packing density and storage density were calculated as 0.76 g cm^{-3} and 0.337 g cm^{-3}, respectively, at 273 K.
To determine the *tapped density* of STA-12(Ni)$_{pwd}$, 0.62 g of activated STA-12(Ni) was transferred to a 10 mL measuring cylinder in a glove box and the cylinder was tapped manually 3000 times onto a wooden support, until a consistent volume of sample was achieved. A tapped density of 0.057 g cm^{-3} was calculated. The bulk density of STA-12(Ni)$_{mono}$ was determined using Archimedes' Law and the following steps were undertaken (**Fig. S19**):
A measuring cylinder was charged with 2.0 mL of degassed silicon oil, and 0.4994 g of a partially dehydrated sample of STA-12(Ni)$_{mono}$, activated using a vacuum oven at 60 °C for 15 h was submerged in the oil. The level of oil increased to 2.4 mL. By using the formula:
density = weight of STA-12(Ni)$_{mono}$/volume displaced
a density of 1.25 g cm^{-3} for partially hydrated STA-12(Ni)$_{mono}$ was obtained. Using the same procedure, a density of 1.40 g cm^{-3} was determined for fully activated STA-12(Ni)$_{mono}$.

The bulk density of STA-12(Ni)$_{pellet}$ was determined using a geometrical method since a precise cylindrical shaped pellet could be obtained after pressurisation. For a pellet of 8 mm diameter and a thickness of 0.81 mm, a volume of 0.04 cm^3 was calculated. Given the mass of the activated pellet was 47.6 mg, the bulk density was determined as 1.19 g cm^{-3}. Five other pellets were also prepared and their density was determined to be 1.10, 1.18, 1.08, 1.11 and 1.16 g cm^{-3}, thus giving an average density of 1.14 g cm^{-3} . The density of fully activated STA-12(Ni)$_{pellet}$ using Archimedes' Law was 1.16 g cm^{-3}, which is consistent with the geometrical method.

Supplementary text

STA-12(Mn) and STA-16(Ni)

To compare the effect of metal lability on any structural change upon NH_3 sorption, we investigated NH_3 sorption in both STA-12(Mn) and STA-16(Ni). STA-16(Ni) is an analogous material to STA-12(Ni), but is composed of the elongated linker, N,N′-4,4′-bipiperidine-bis(methylenephosphonate. Both these materials retain rhombohedral symmetry upon activation. STA-12 (Mn), unlike STA-12(Ni) did not show a high NH_3 uptake at *ca.* 1 mbar, rather a sudden uptake at 16 mbar was observed. Previous experiments have shown that STA-12(Mn) undergoes a structural change possibly at the surface due to a reconstructive transformation upon dehydration, likely resulting in a smaller pore. This suggests that this transformation is reversible, and at 16 mbar, enough NH_3 has been dosed to generate the original rhombohedral structure affording an entropic driving force (followed by a decrease with further loading) as inclusion of NH_3 expands the pores. STA-16(Ni) in contrast shows a lower NH_3 uptake (11.2 mmol g^{-1} at 273 K, 1 bar) and comparatively low NH_3 uptake at lower pressures, with hysteresis observed during the desorption process. This further supports that

the low pressure NH_3 uptake in STA-12(Ni) is partly driven by pore expansion. A larger pore size does not necessarily drive higher NH_3 uptake, as larger pores may decrease guest-guest and host-guest interactions at low partial pressures. Even at 1 bar, STA-16(Ni) has not reached saturation, compared to STA-12(Ni).

NH_3 sorption in fully activated STA-12(Ni)$_{mono}$

NH_3 loading into fully activated STA-12(Ni)$_{mono}$ led to collapse of the monolith to afford STA-12(Ni)$_{pwd}$. This is consisent with diffusion of NH_3 through the material causing an expansion of the bulk sample leading to collapse of the superstructure. In contrast, STA-12(Ni)$_{pellet}$ has a compact surface with a minimised amount of interstitial space leading to slower diffusion of NH_3 and retention of overall structural integrity. Interestingly, activated STA-12(Ni)$_{mono}$ retains its bulk structure in the presence of N_2 and CO_2.

In situ IR spectroscopy

Partially hydrated STA-12(Ni), $[Ni_2(L)(H_2O)_2]$, shows peaks at 3679 and 1587 cm^{-1} assigned to the Ni–OH_2 stretching and bending modes, respectively. This is entirely consistent with literature reports for water sorption in STA-12(Ni), where the water bound to Ni form strong hydrogen bonds with the P=O group to form P^+–O^-…H interactions. No broad peaks are observed between 3150 to 3400 cm^{-1}, confirming that there is no physisorbed water within the pore. On dosing with 1% NH_3, both peaks at 3679 and 1587 cm^{-1} disappear and peaks corresponding to both chemisorbed and physisorbed NH_3 appears.

Supplementary Figures

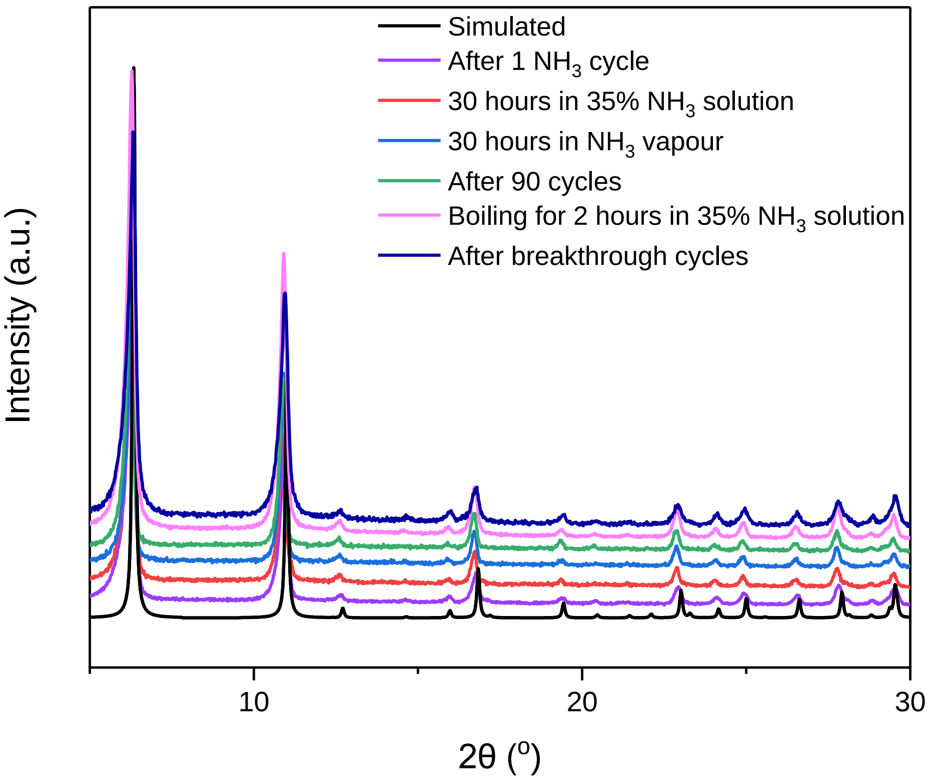

Figure S1: PXRD patterns of STA-12(Ni). Simulated (black); after 1 NH_3 cycle (violet); after 30 h in a 18 M (35%) NH_3 solution (red); after 30 h under NH_3 vapour (blue); after 90 NH_3 adsorption-desorption cycles (green); after boiling in a 18 M NH_3 solution for 2 h (pink); after 5 breakthrough cycles under a flow of 1000 ppm NH_3 at 190, 145 and 100 °C.

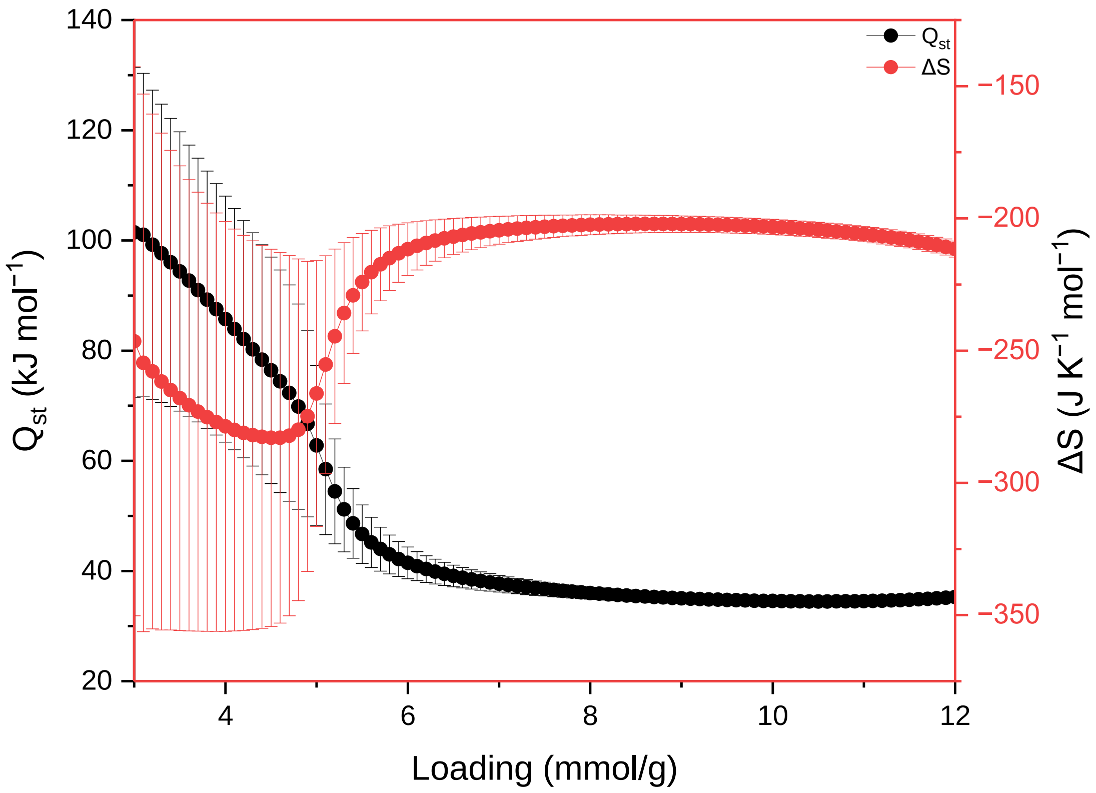

Figure S2: Isosteric heat and entropy of adsorption of STA-12(Ni) at different NH_3 loadings. Calculations were carried out using isotherms obtained between 273 and 302 K. The value of Q_{st} was within the range calculated by the molecular simulations (*ca.* 38 kJ mol^{-1}). Large errors at low-loadings are attributed to both fast adsorption kinetics and chemisorption of NH_3 which the Van't Hoff isochore does not account for.

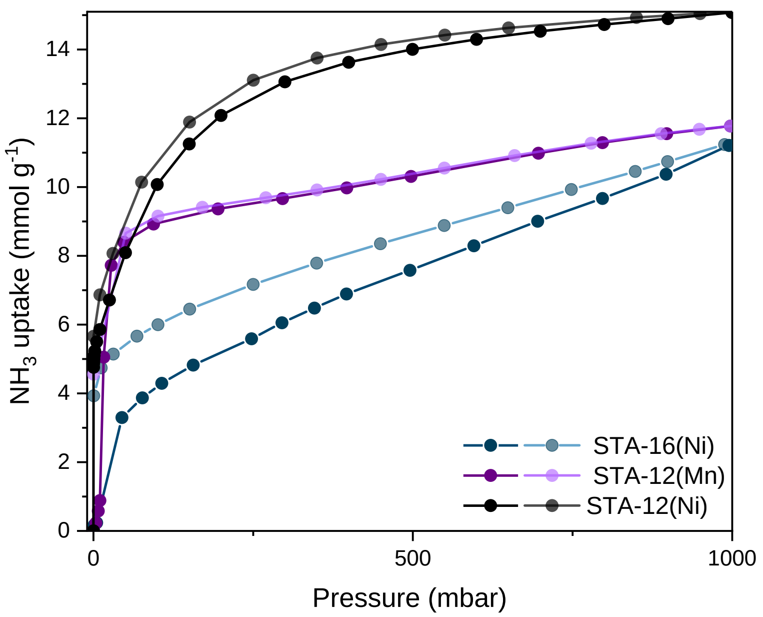

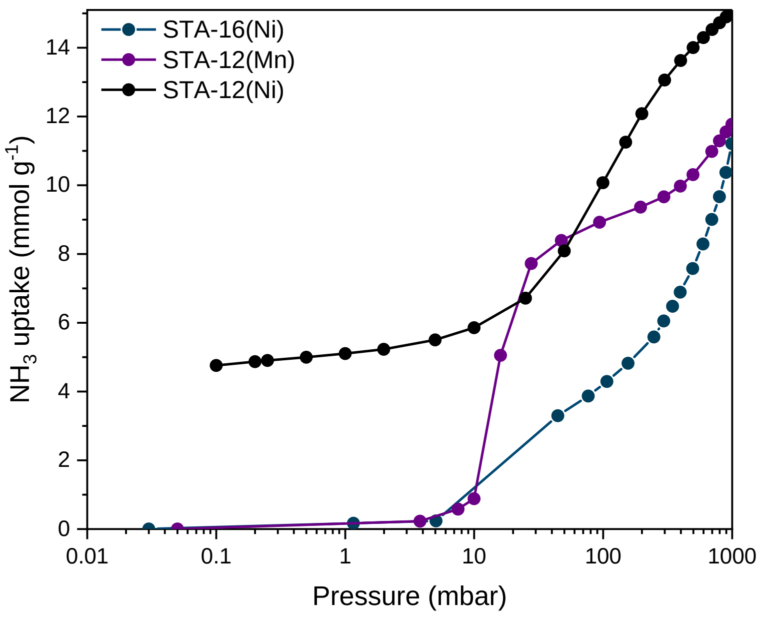

Figure S3: Gravimetric NH_3 isotherms of STA-12(Mn) (purple), STA-16(Ni) (blue-green) and STA-12(Ni) (black) at 273 K. The low-pressure region (up to 100 mbar) is highlighted in the bottom graph; desorption isotherm is omitted for clarity.

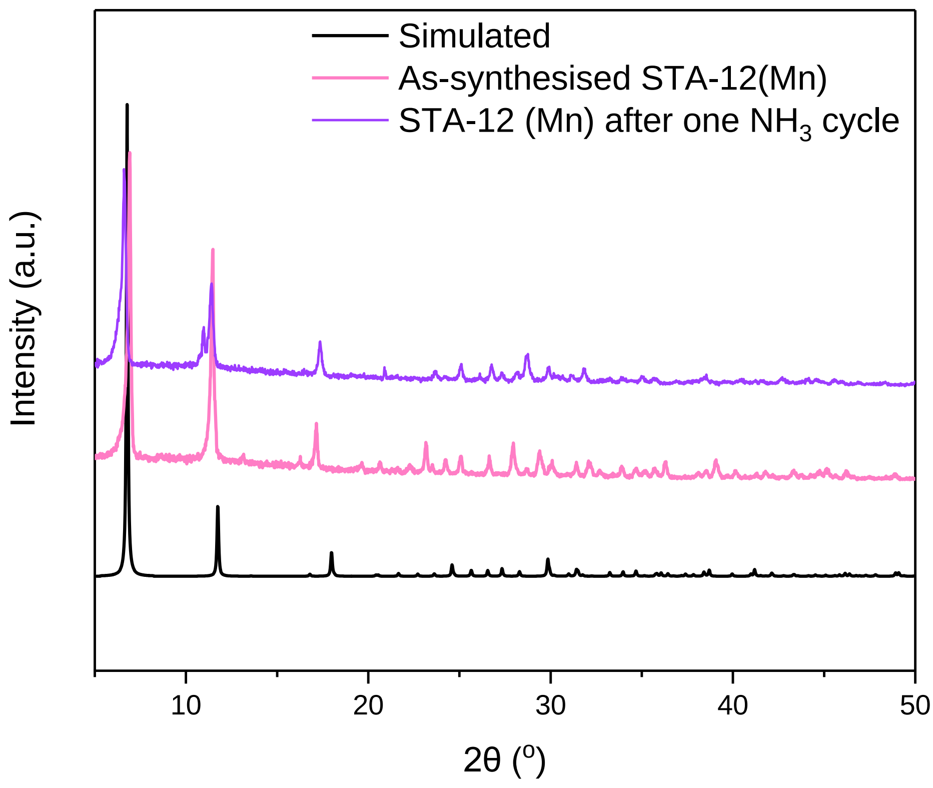

Figure S4: PXRD patterns of simulated (black), as-synthesised (pink) and NH_3@STA-12(Mn) (purple), confirming that the material has slightly decomposed upon exposure to NH_3.

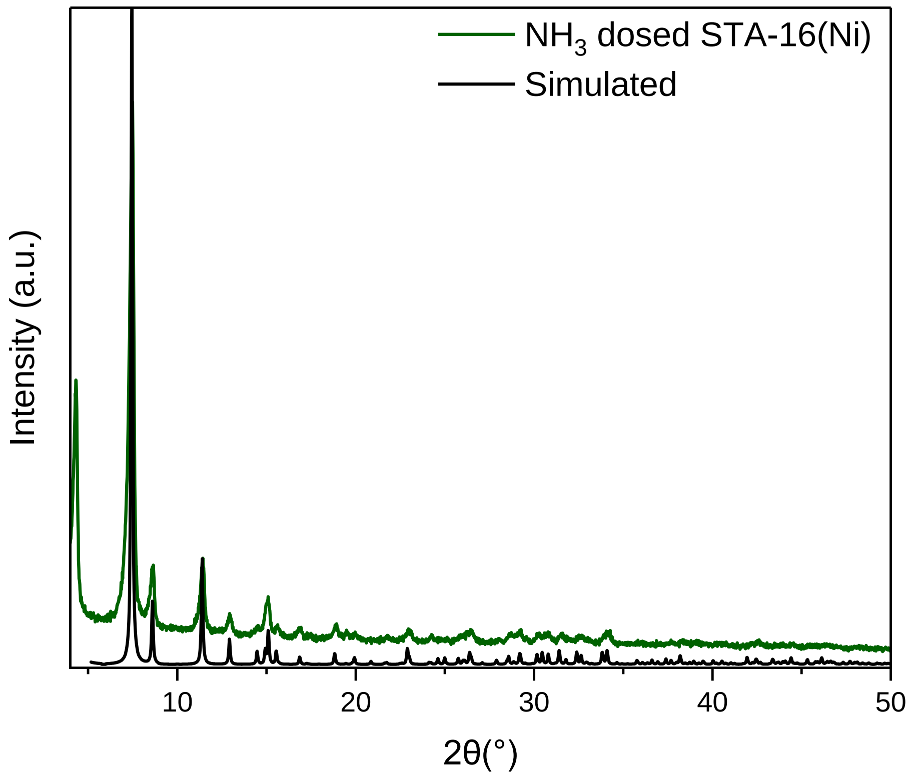

Figure S5: PXRD pattern of simulated STA-16(Ni) (black) and NH_3@STA-16(Ni) (green), confirming retention of crystallinity upon exposure to NH_3.

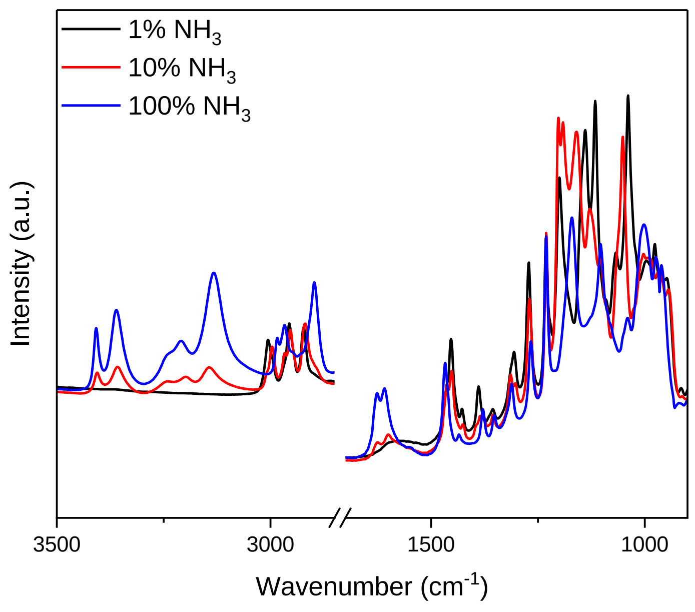

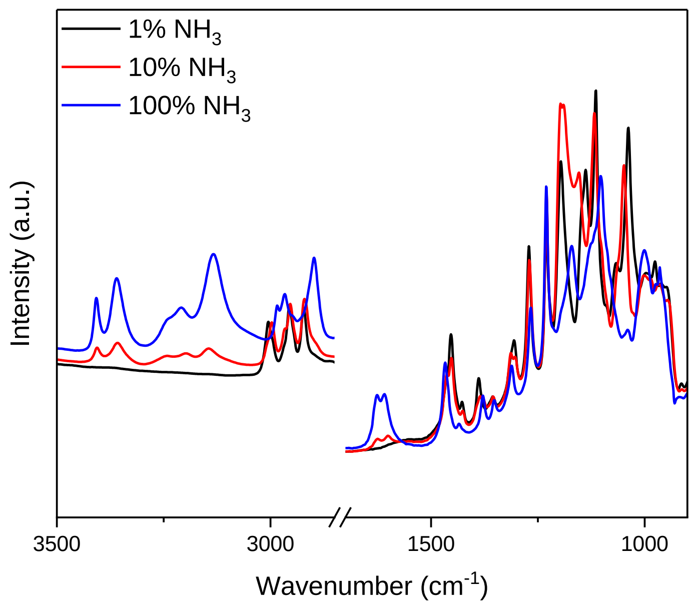

Figure S6: IR spectra of STA-12(Ni) under 1% (black), 10% (red) and 100% (blue) NH_3 flow at 25 °C (top) and 275 °C (bottom).

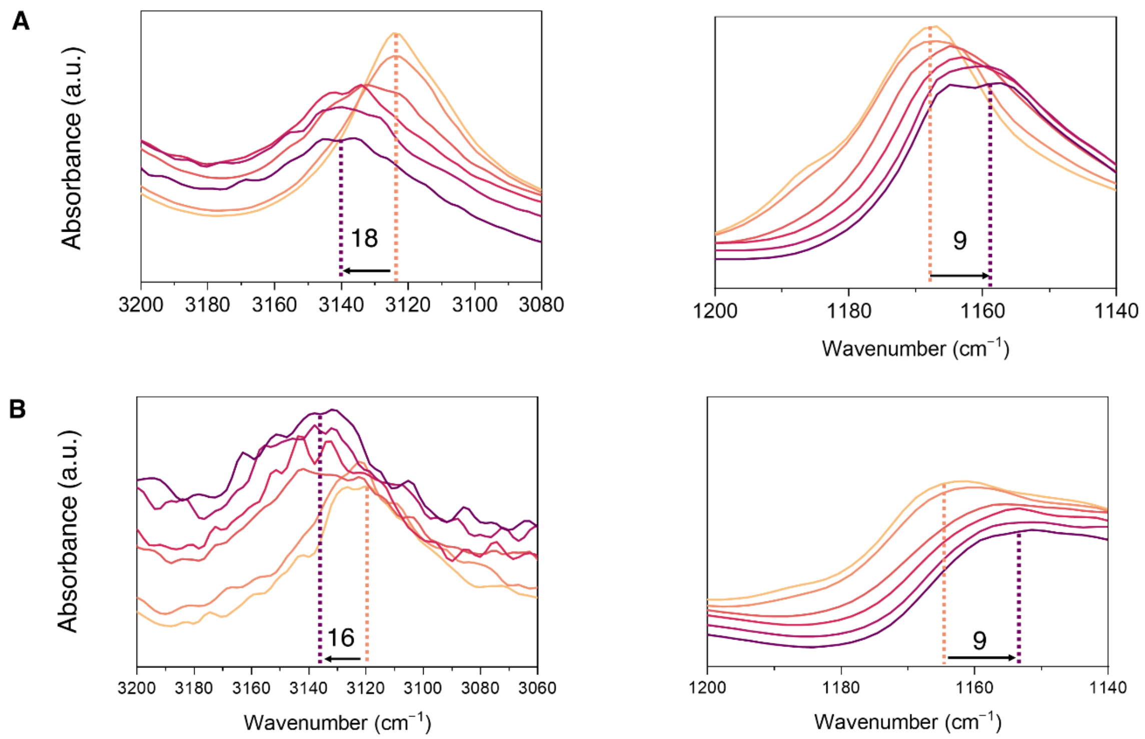

Figure S7: IR spectra showing symmetric (v_s) (3130 cm^{-1}) and N-H wagging modes (v_w) for chemisorbed NH_3 (1180 cm^{-1}) upon NH_3 loading into fully (**A**) and partially activated (**B**) STA-12(Ni). Increased NH_3 loading [yellow (1%)→purple (100%)] causes a shift in both bands, where the v_s band blue shifts, and the v_w band red shifts, suggesting a strengthening of the hydrogen bond interactions between the physisorbed and chemisorbed NH_3 on increasing loading of NH_3.

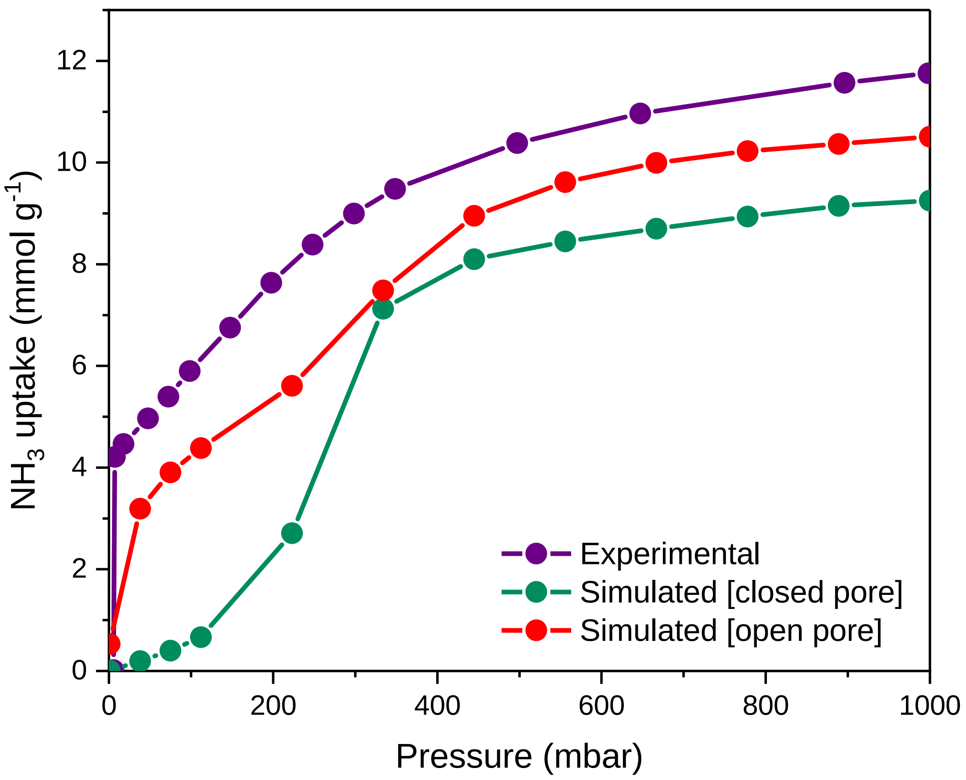

Figure S8: Simulated NH_3 uptake capacities for elliptical shaped (closed pore) activated STA-12(Ni) (green) and rhombohedral (open pore) STA-12(Ni) (red). The simulation shows a distinct uptake at low pressure for the rhombohedral framework attributable to accessible vacant metal sites within the pore.

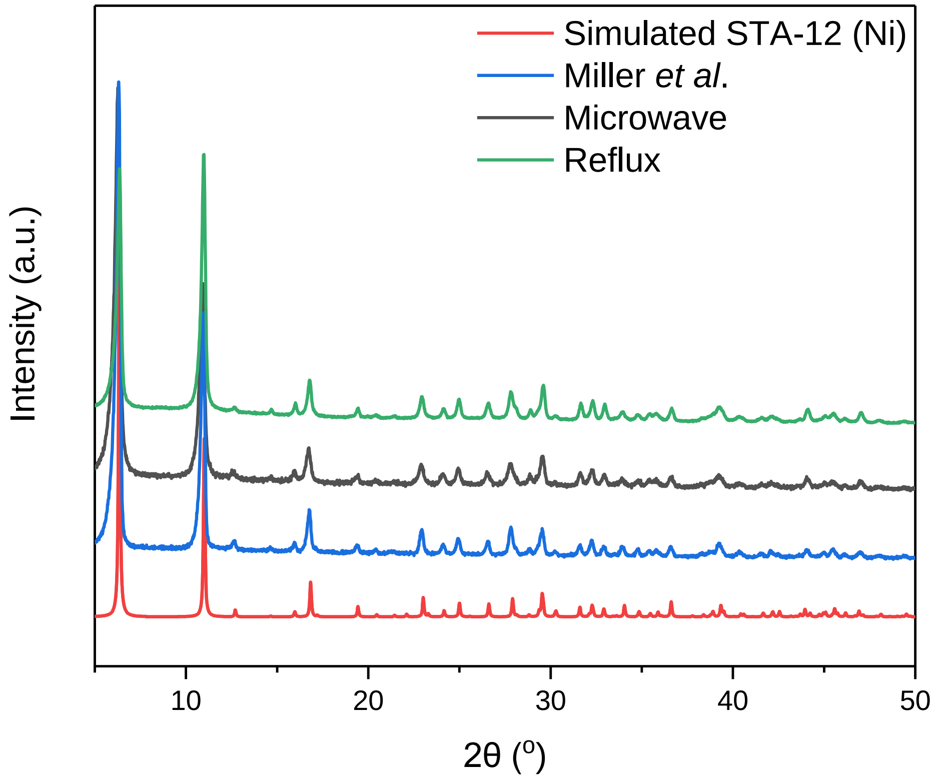

Figure S9: PXRD pattern of simulated STA-12(Ni) (red), and of isolated STA-12(Ni) obtained through various synthetic methods from the literature (blue), microwave route (black) and reflux route (green).

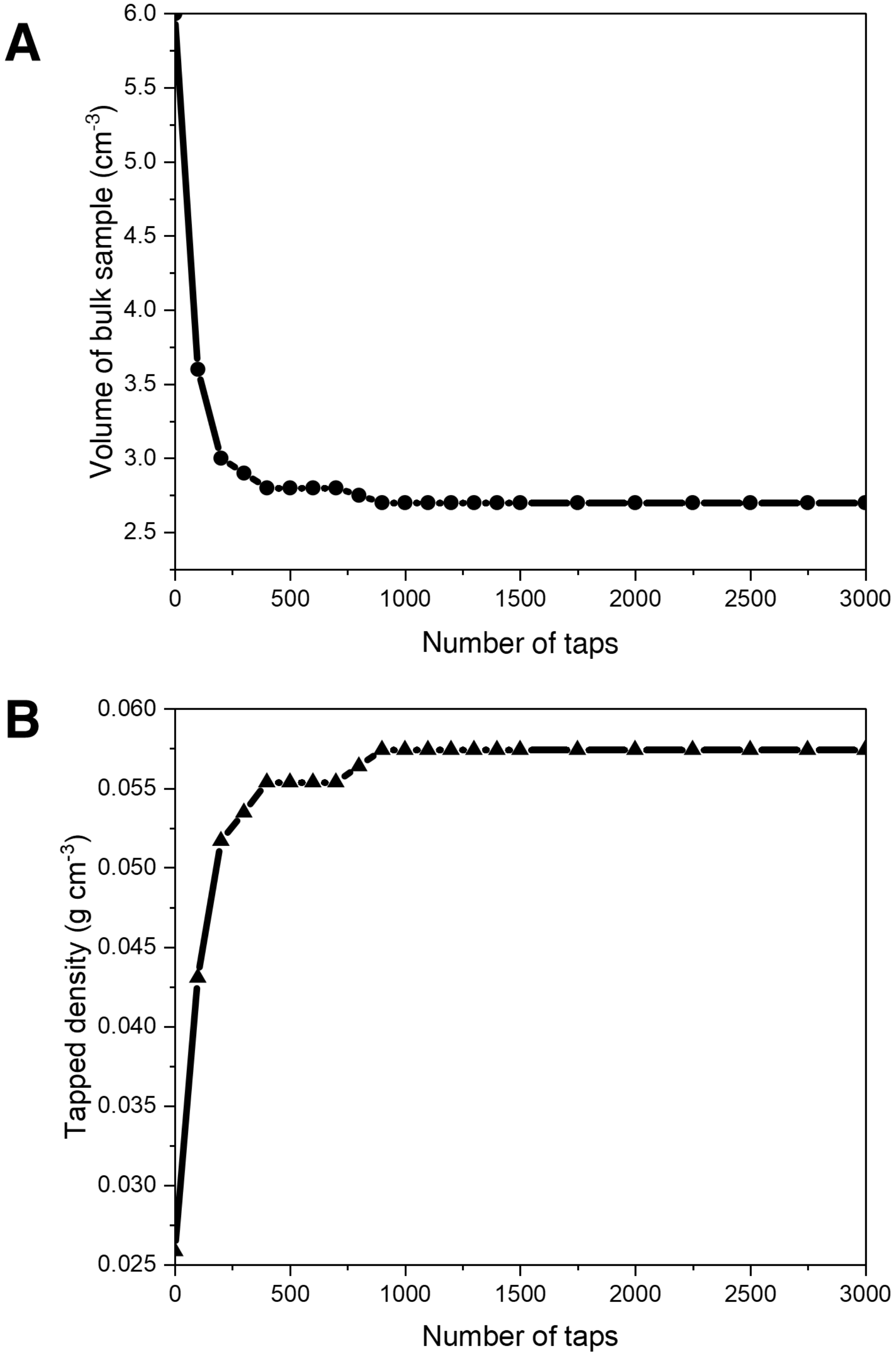

Figure S10: (**A**) variation of volume of the bulk powder sample of activated STA-12(Ni)$_{pwd}$ with increasing number of taps. (**B**) shows how the tapped density changed with increased number of taps.

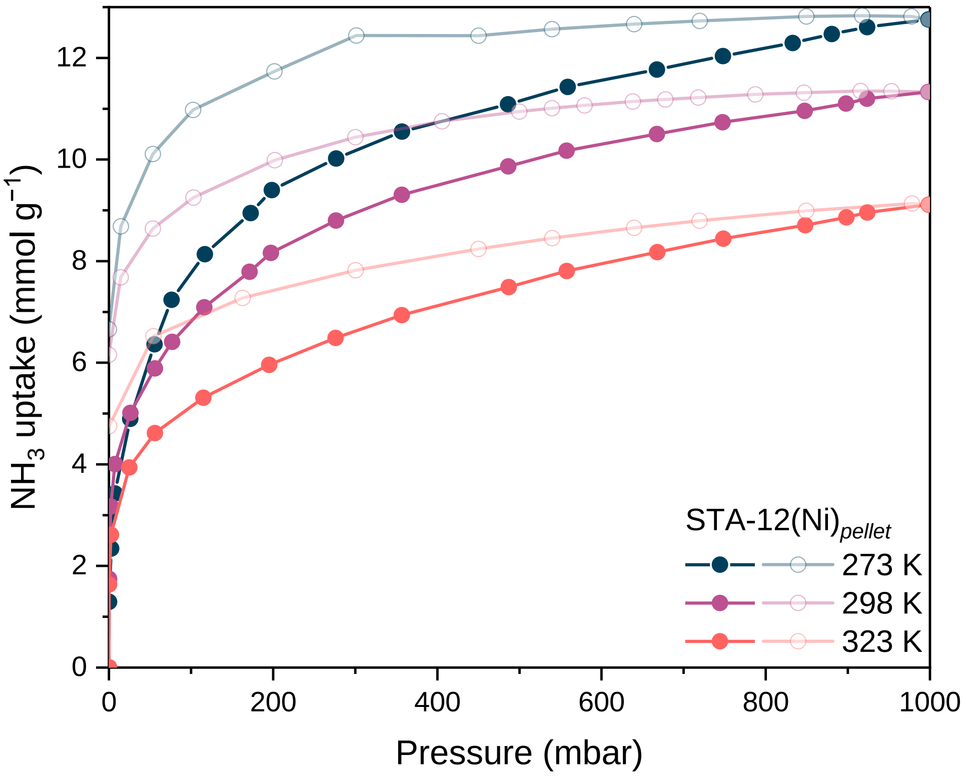

Figure S11: Gravimetric isotherms of NH_3 in STA-12(Ni)*pellet* between 273 K to 298 K. Hysteresis during desorption is attributed to slow diffusion of guest molecules within the bulk material; this effect decreases with increase in temperature as expected as molecules can diffuse through more quickly at higher temperatures.

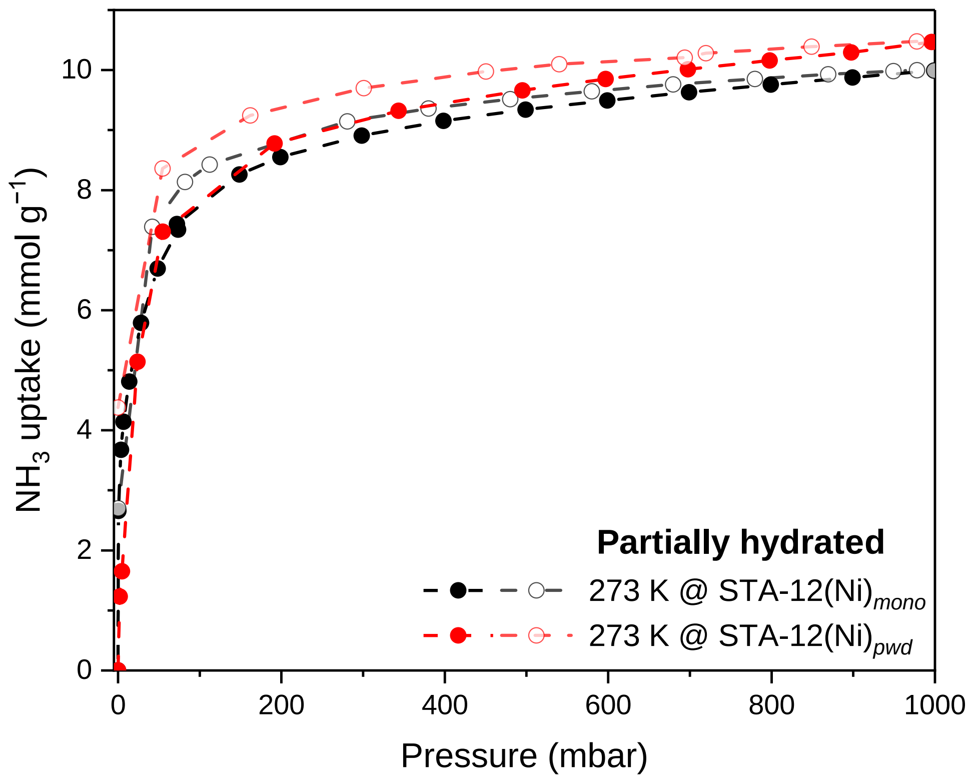

Figure S12: Gravimetric isotherms of NH_3 in partially hydrated STA-12(Ni)$_{mono}$ and STA-12(Ni)$_{pwd}$ at 273 K.

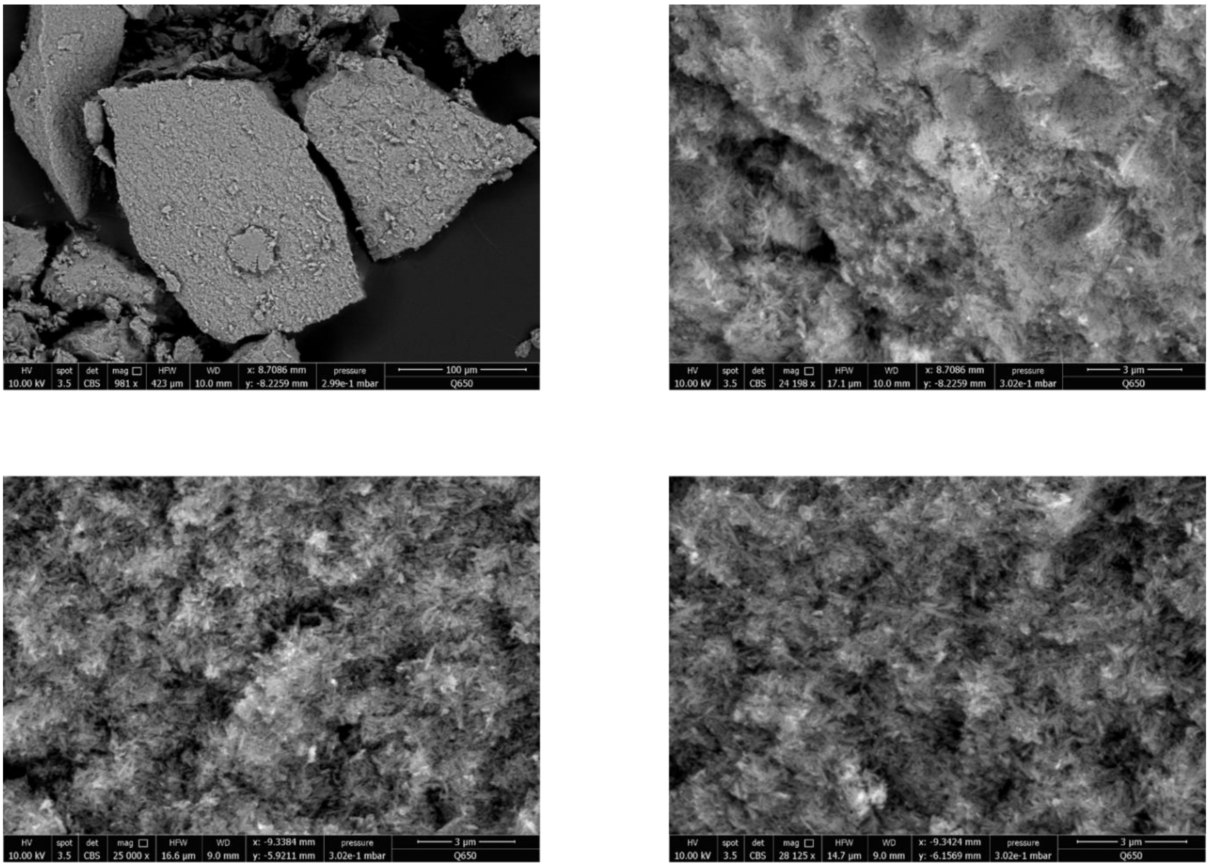

Figure S13: SEM images of STA-12(Ni)$_{mono}$.

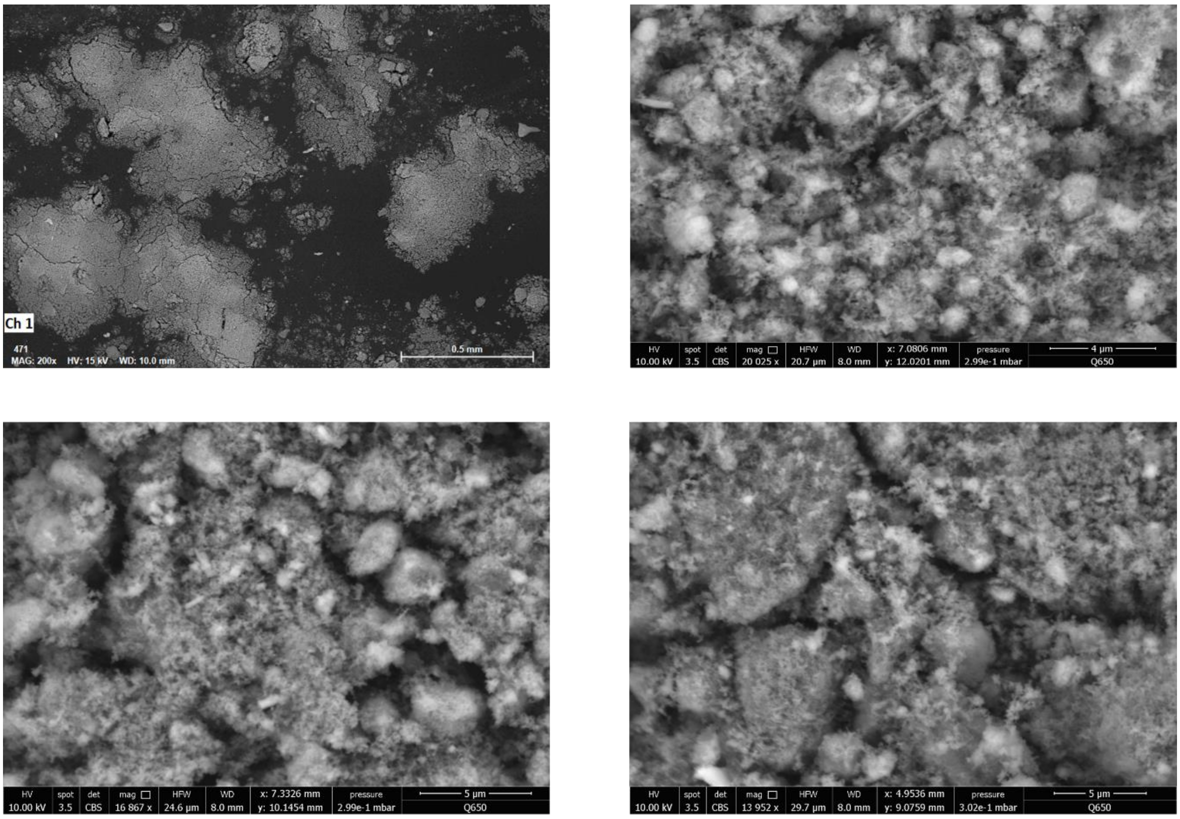

Figure S14: SEM images of STA-12(Ni)$_{pwd}$.

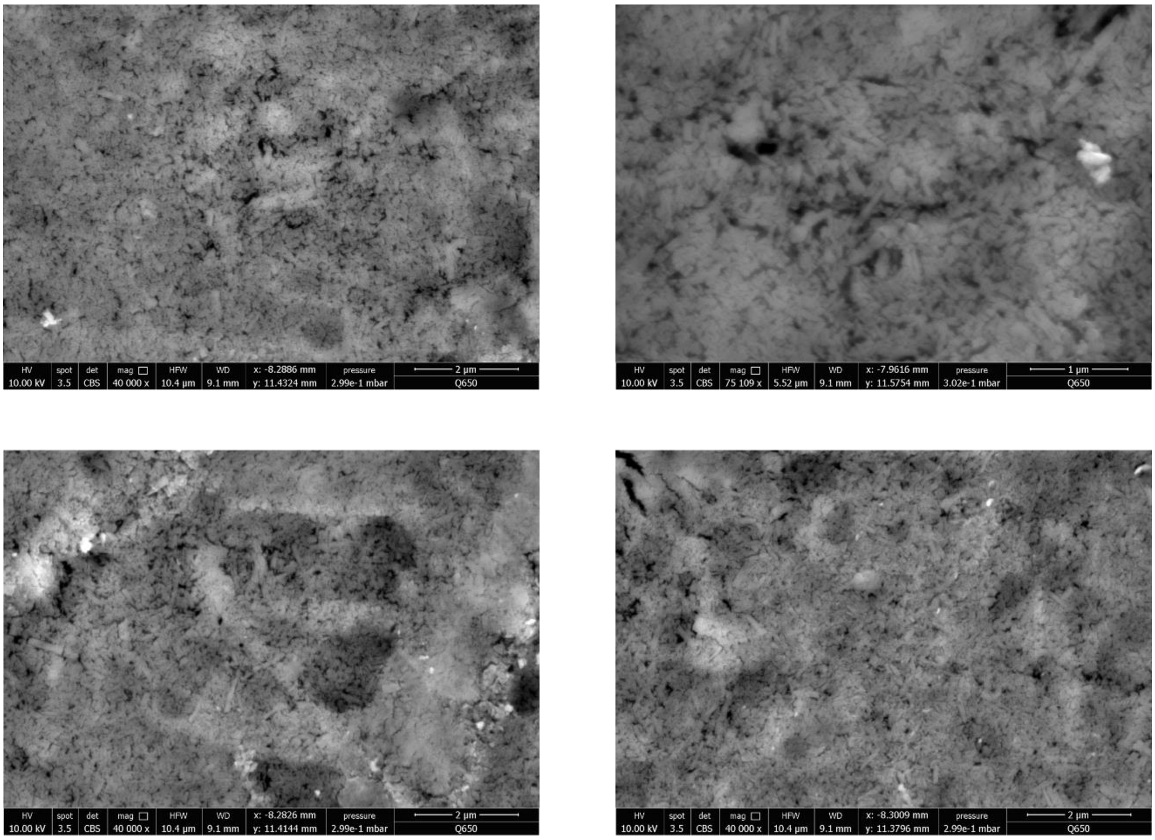

Figure S15: SEM images of STA-12(Ni)$_{pellet}$.

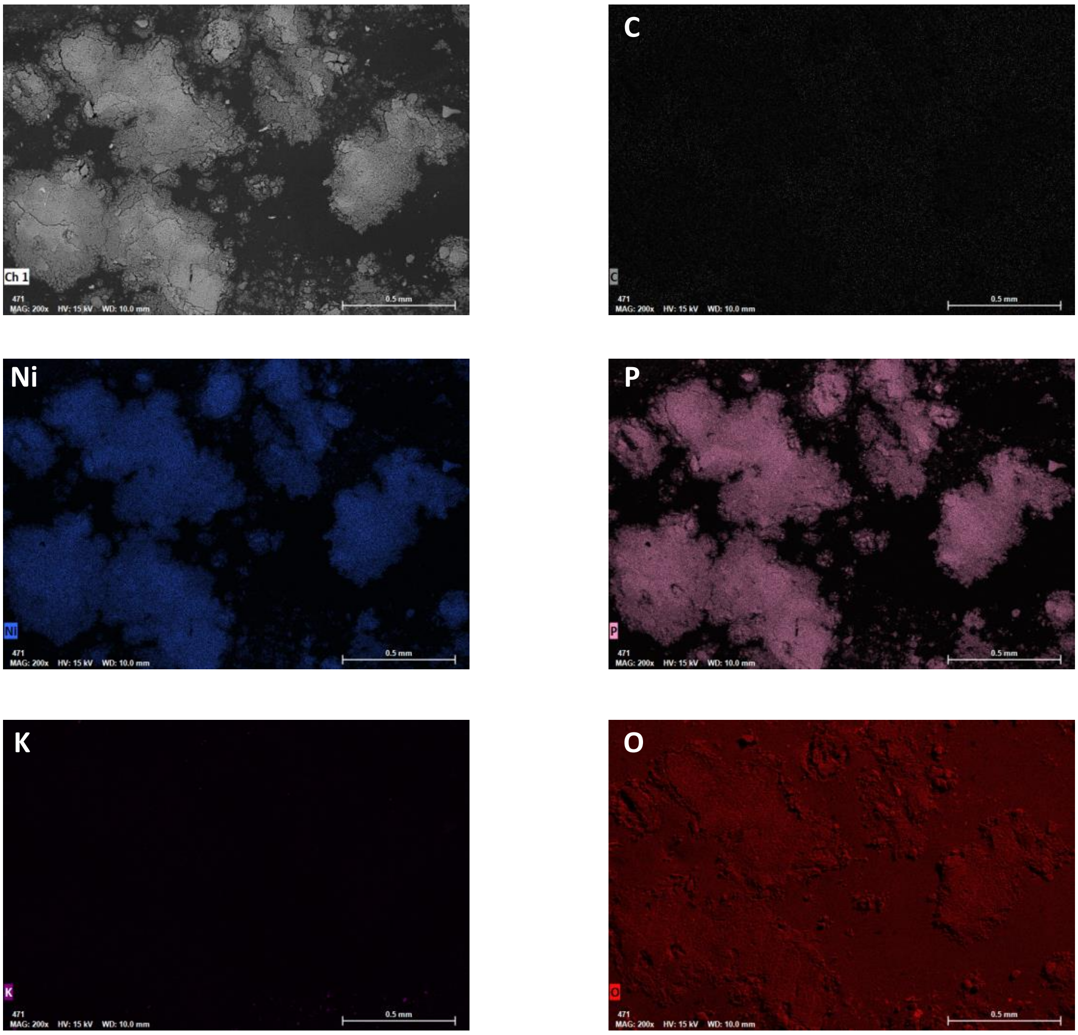

Figure S16: SEM and EDX (C, Ni, P, K and O) images of STA-12(Ni)$_{pwd}$.

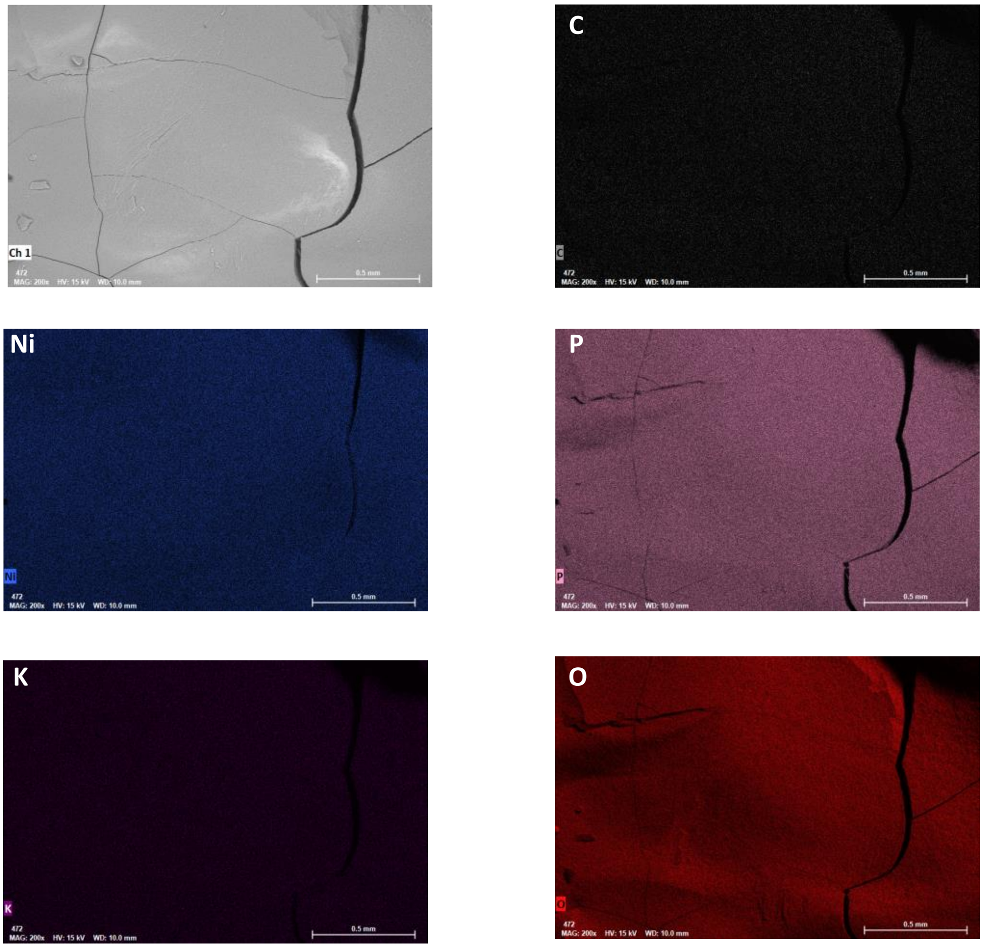

Figure S17: SEM and EDX (C, Ni, P, K and O) images of STA-12(Ni)$_{mono}$. K^+ ions were observed distributed throughout the sample.

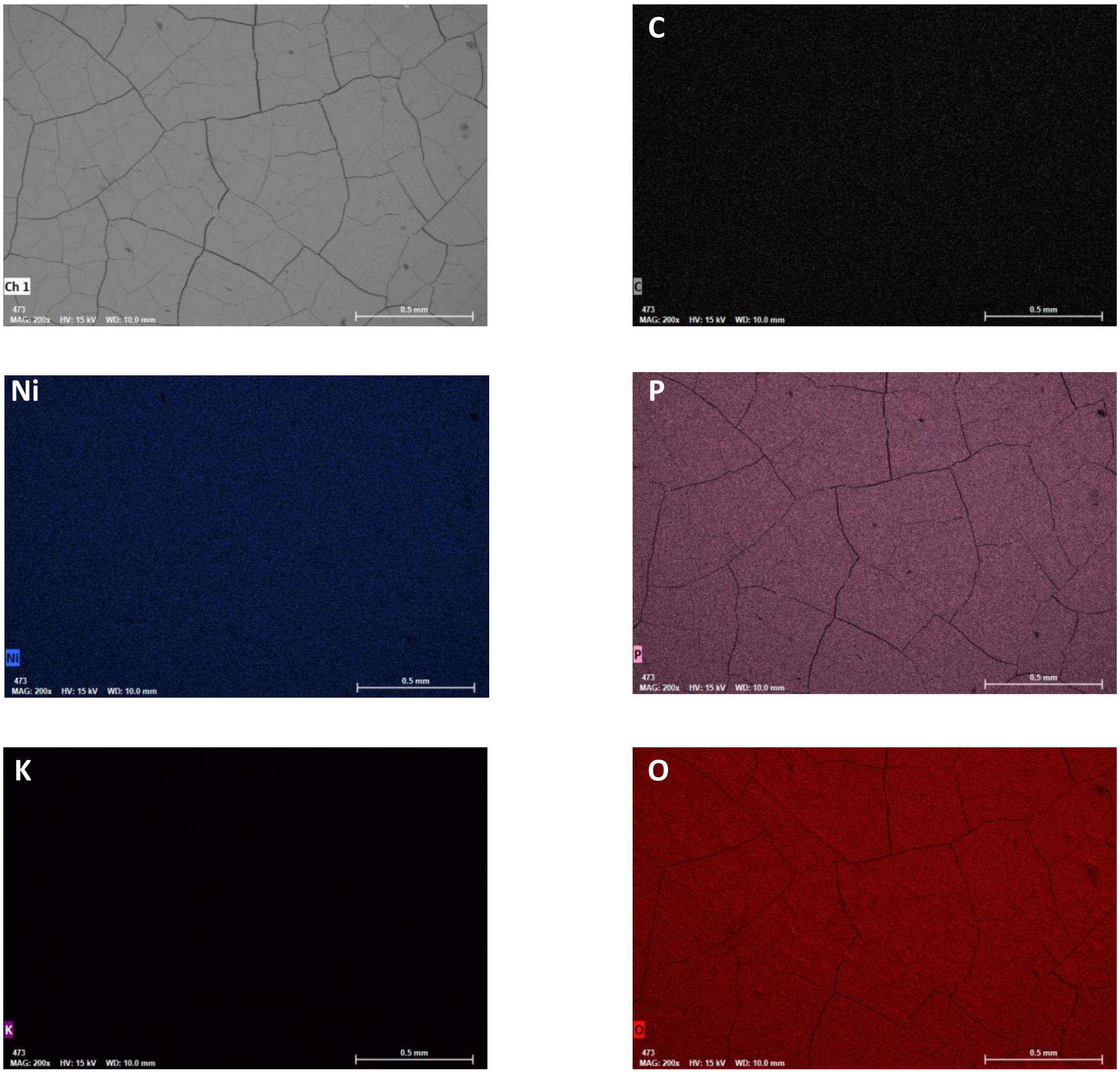

Figure S18: SEM and EDX (C, Ni, P, K and O) images of STA-12(Ni)$_{pellet}$.

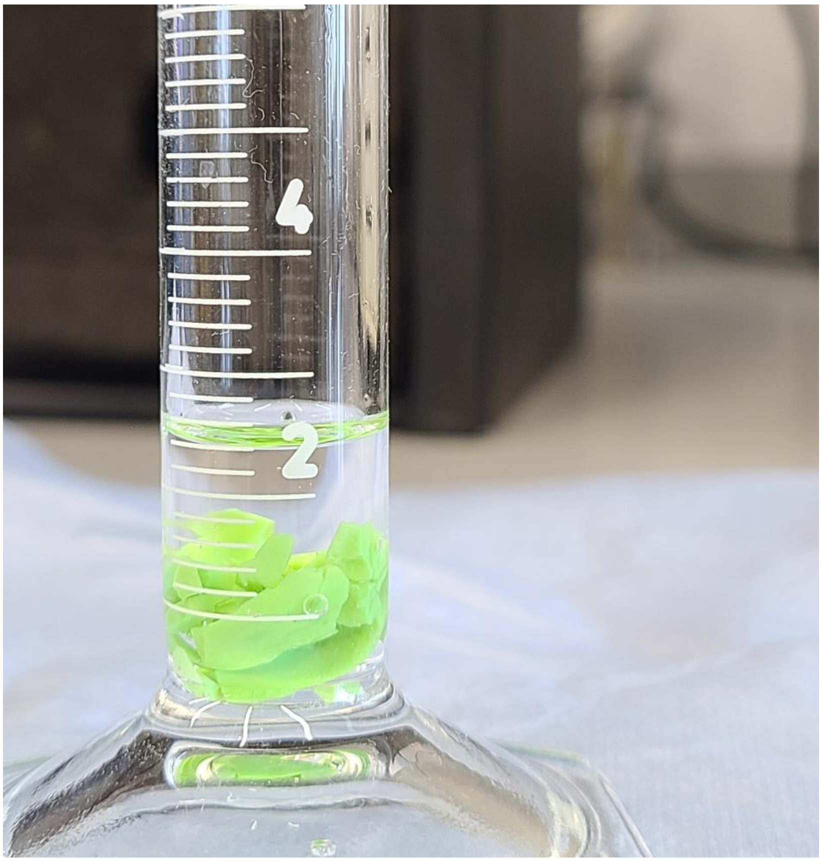

Figure S19: Volume of silicon oil (2.4 mL) after addition of partially hydrated STA-12(Ni)$_{mono}$; original volume of silicon oil was 2.0 mL. Using Archimedes' Law, a density of 1.25 g cm^{-3} was calculated.

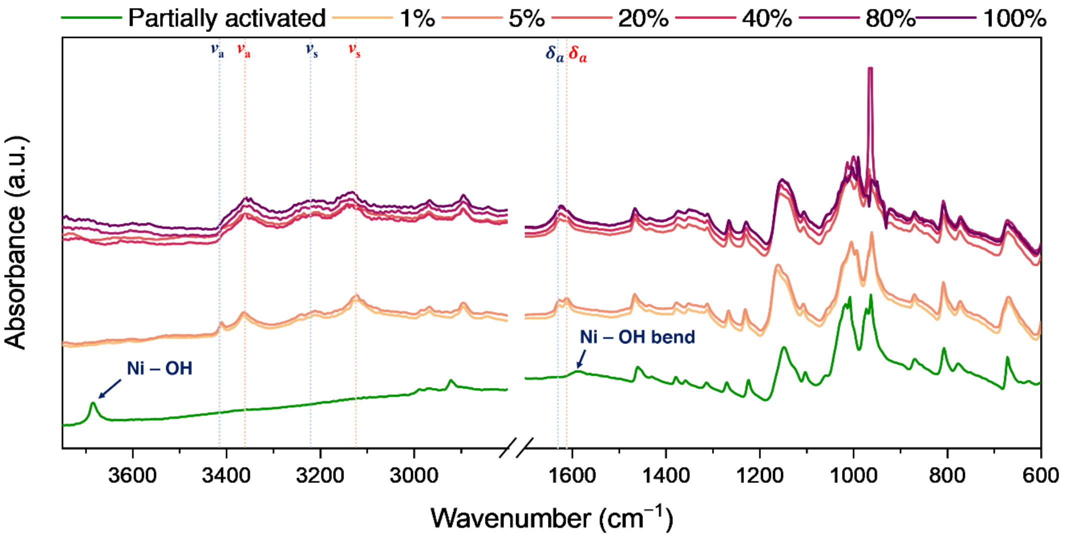

Figure S20: *In situ* IR spectra of partially hydrated STA-12(Ni) on increasing NH_3 loading. The antisymmetric (ν_a), symmetric (ν_s) NH stretching modes and the NH degenerate deformation mode (δ_a) for both physisorbed (blue) and chemisorbed NH_3 (red) are highlighted. Under a flow of 1% NH_3, bands associated with chemisorbed H_2O molecules (Ni-OH_2 stretching and bending modes) disappear, confirming that the NH_3 molecules are replacing bound H_2O molecules at the Ni^{II} sites.

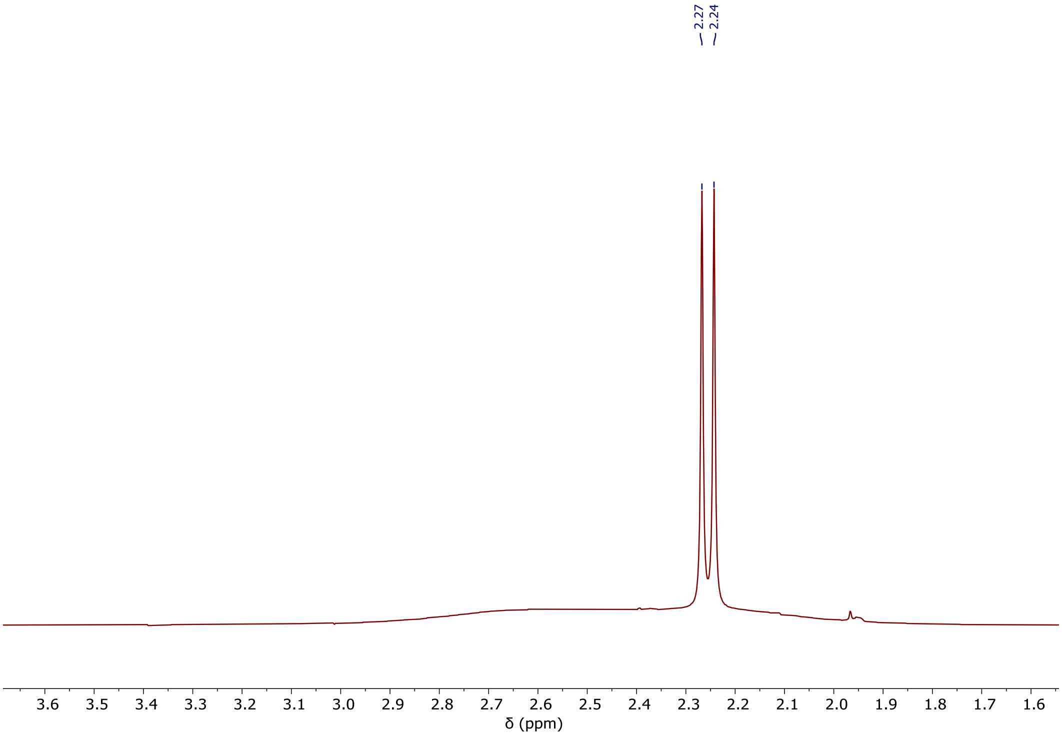

Figure S21: ^{1}H NMR spectrum (500 MHz, D_2O/NaOH, 298 K) of *N,N'*–piperazine-bis(methylenephosphonic acid). The very broad peak is attributed to protons on the piperazine ring; the broadening is due to rapid conformational flipping between axial and equatorial positions.

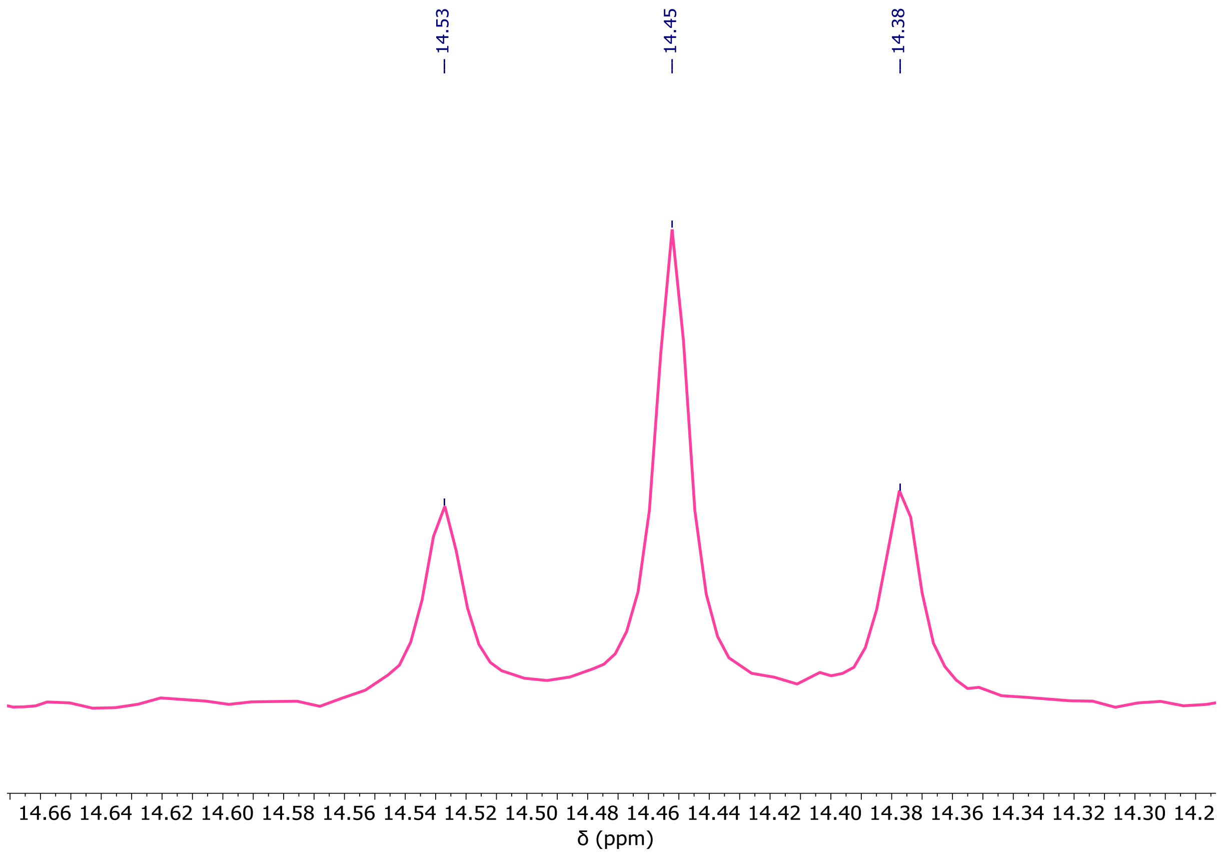

Figure S22: ^{31}P NMR spectrum (162 MHz, D_2O/NaOH, 298 K) of *N,N'*–piperazine-bis(methylenephosphonic acid).

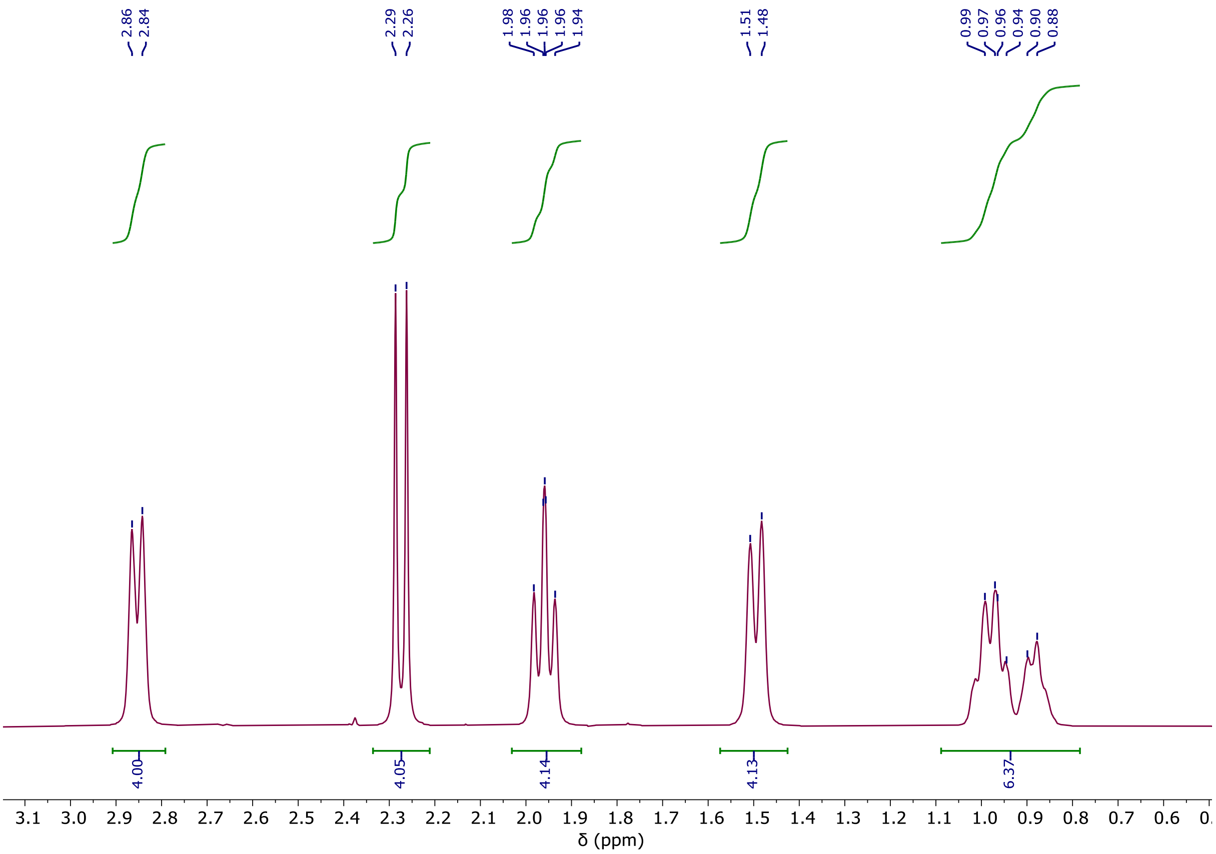

Figure S23: ^{1}H NMR spectrum (500 MHz, D_2O/NaOH, 298 K) of *N,N'*-4,4'-bipiperidine-bis(methylenephosphonic acid).

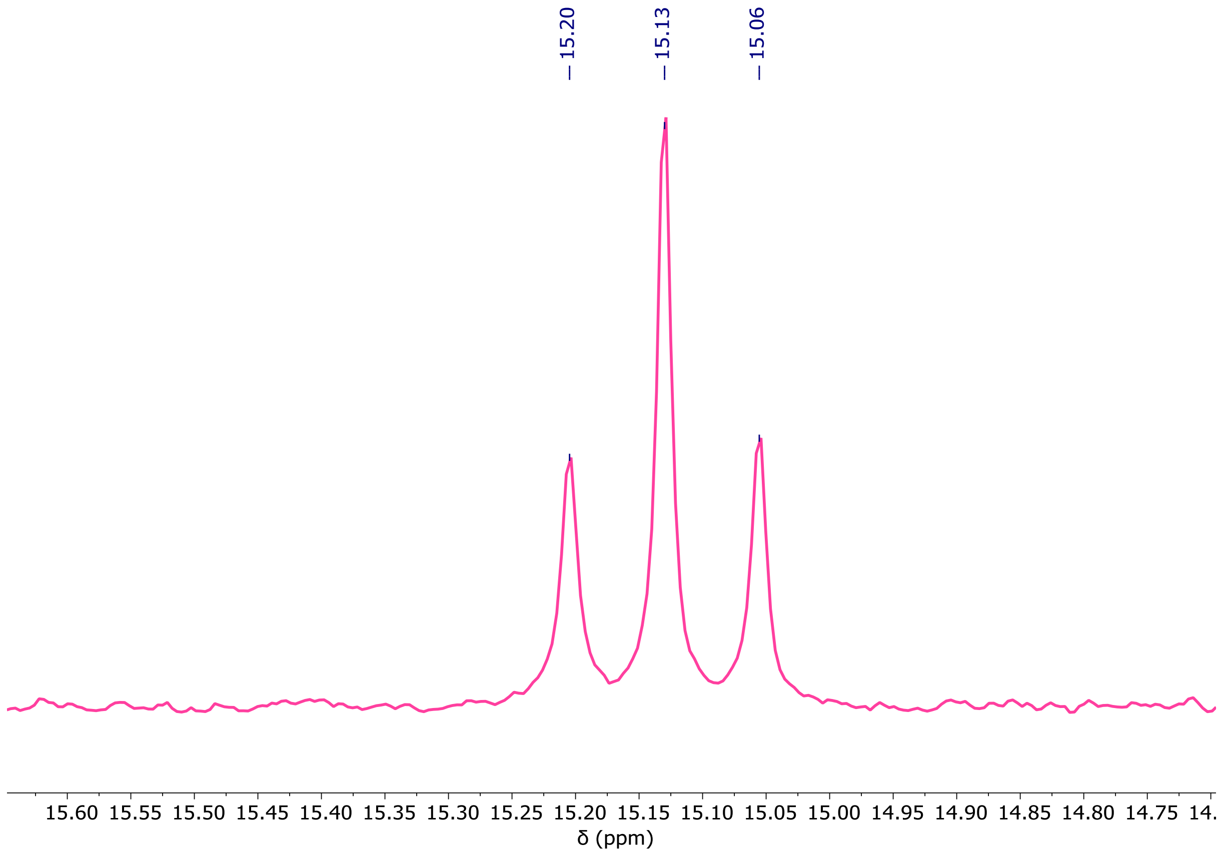

Figure S24: ^{31}P NMR spectrum (162 MHz, D_2O/NaOH, 298 K) of *N,N'*-4,4'-bipiperidine-bis(methylenephosphonic acid).

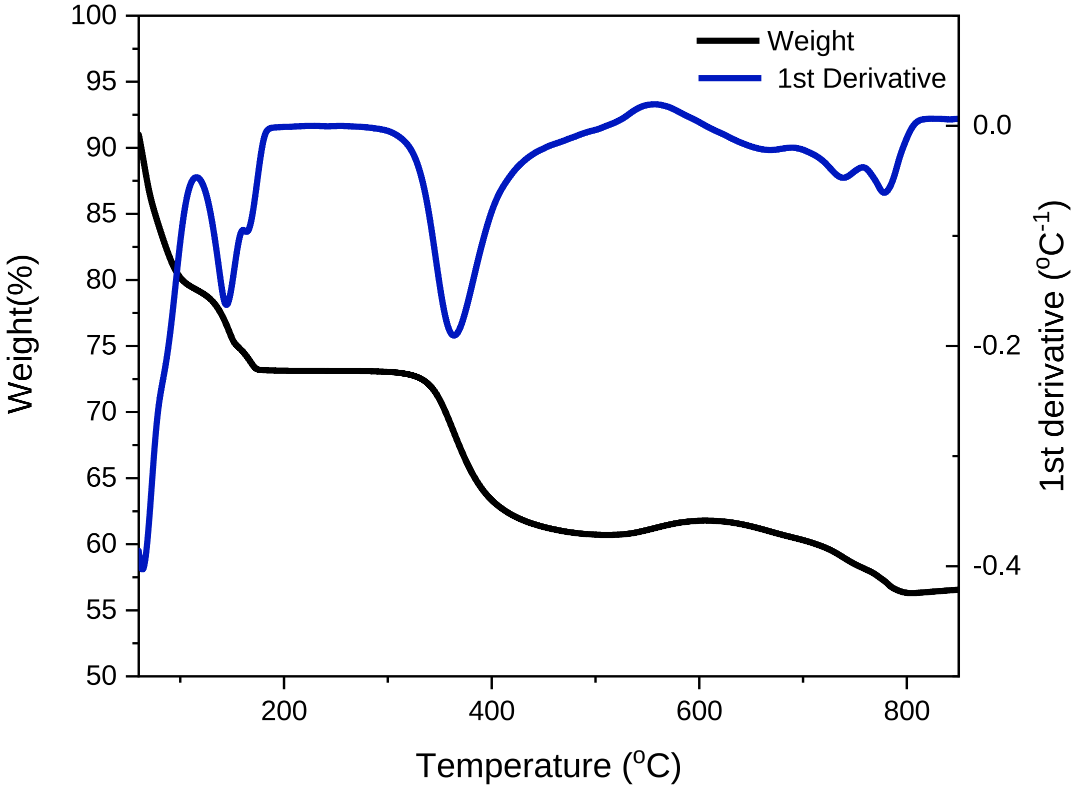

Figure S25: TGA of STA-12(Ni) under an air flow (black) with the 1st derivative of the plot shown in blue. The plot suggests *that* the material is stable until 330 °C. The increase of weight at 600 ºC is assigned to formation of nickel pyrophosphate ($Ni_2P_2O_7$) under air flow.

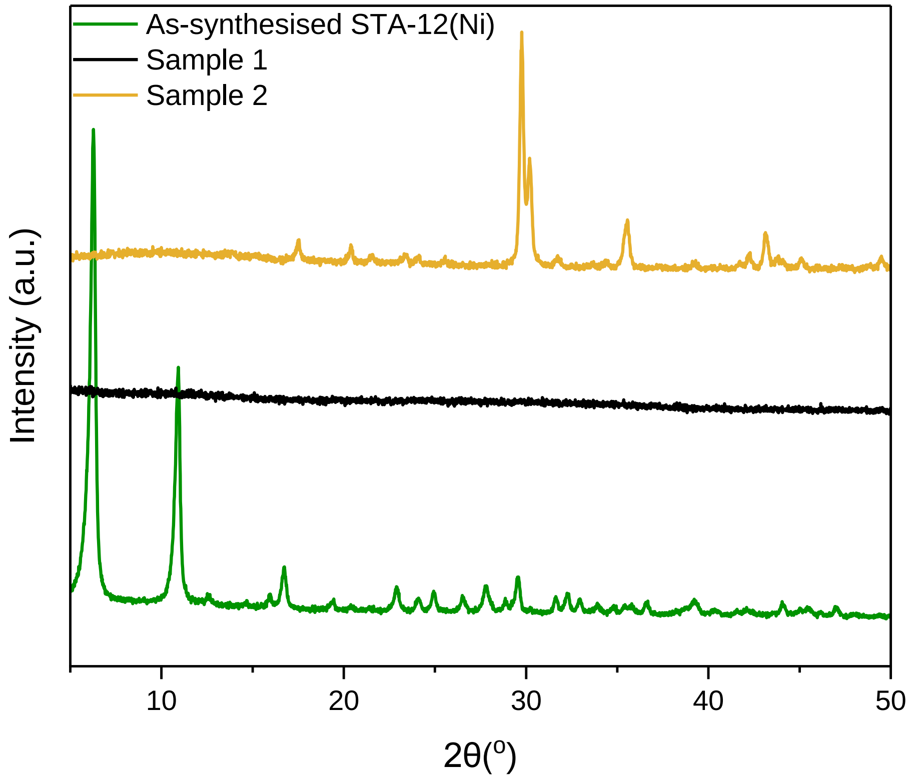

Figure S26: PXRD patterns of STA-12(Ni) (green) heated up to 600 °C under N_2 flow (black, sample 1) and air flow (yellow, sample 2). The PXRD pattern of sample 2 is assigned to formation of nickel pyrophosphate ($Ni_2P_2O_7$) under air flow.

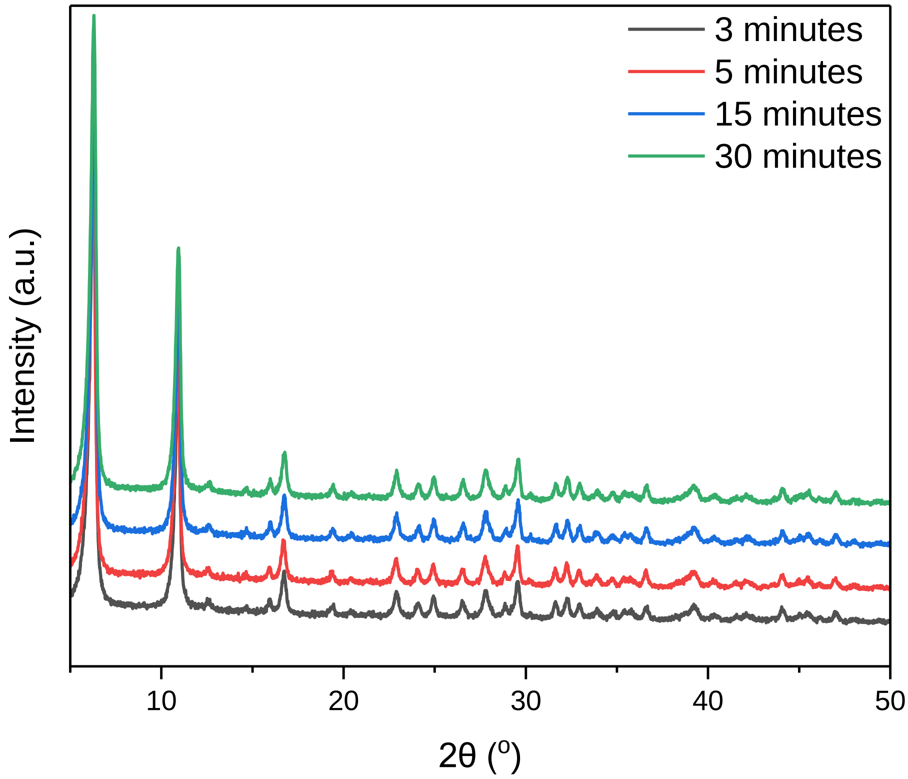

Figure S27: PXRD patterns of STA-12(Ni) synthesised by microwave over different time scales; 3 minutes (black), 5 minutes (red), 15 minutes (blue) and 30 minutes (green). No significant change in intensities of the peaks observed, suggesting that nucleation and synthesis of STA-12(Ni) crystallites was completed within a short period of time, less than 3 mins.

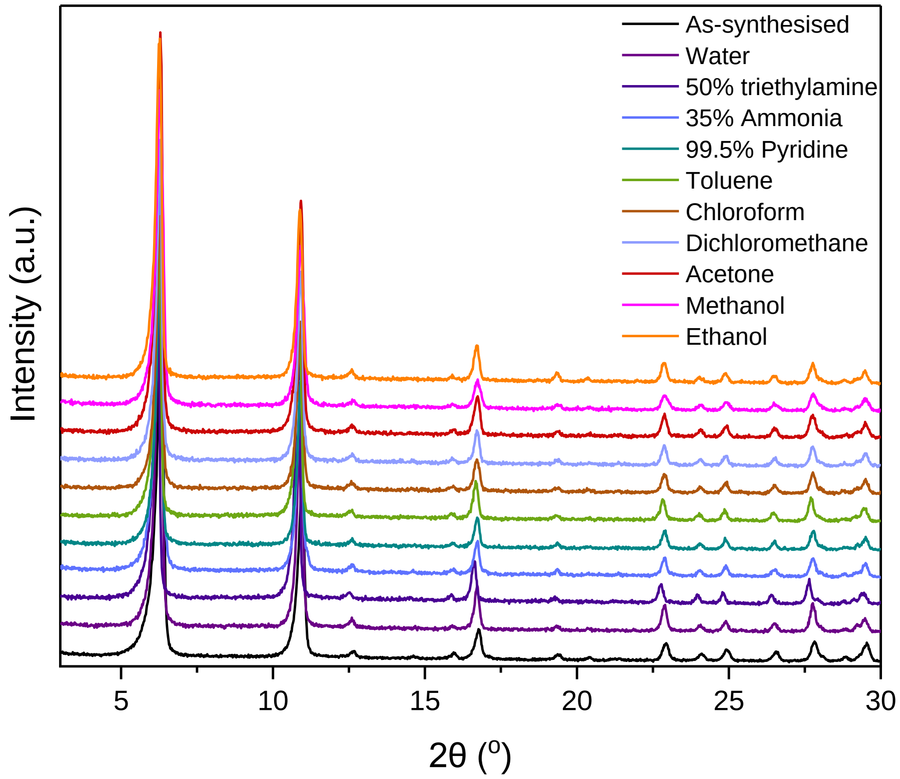

Figure S28: PXRD patterns of STA-12(Ni) after soaking for 30 h in various solutions.

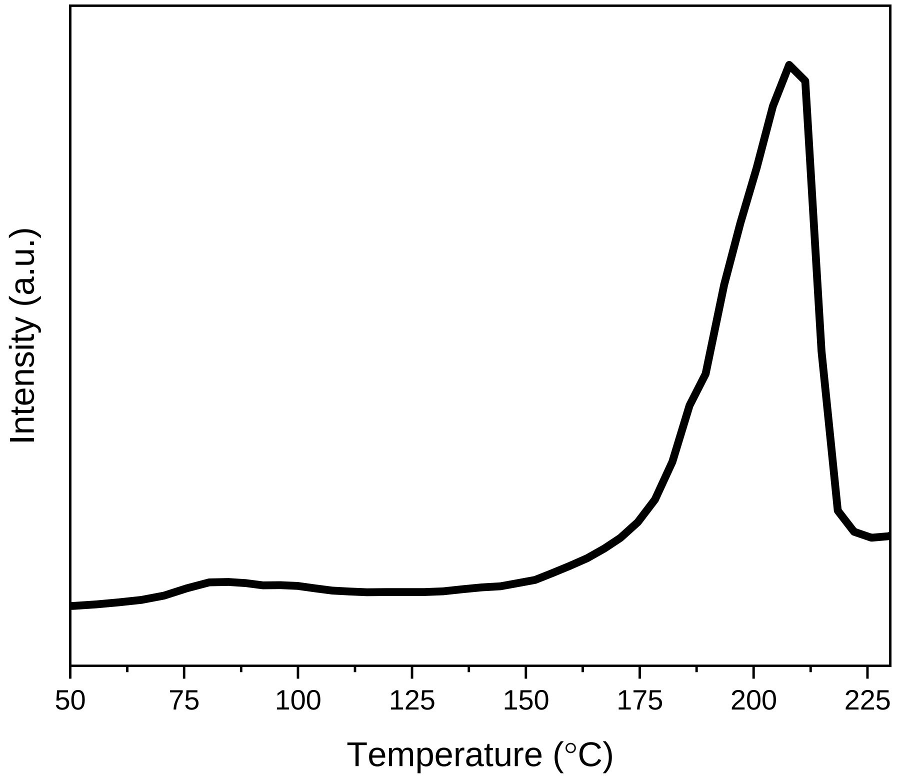

Figure S29: Temperature programmed desorption (TPD) plot of NH_3 loaded STA-12(Ni); complete desorption occurred at approximately 210 °C.

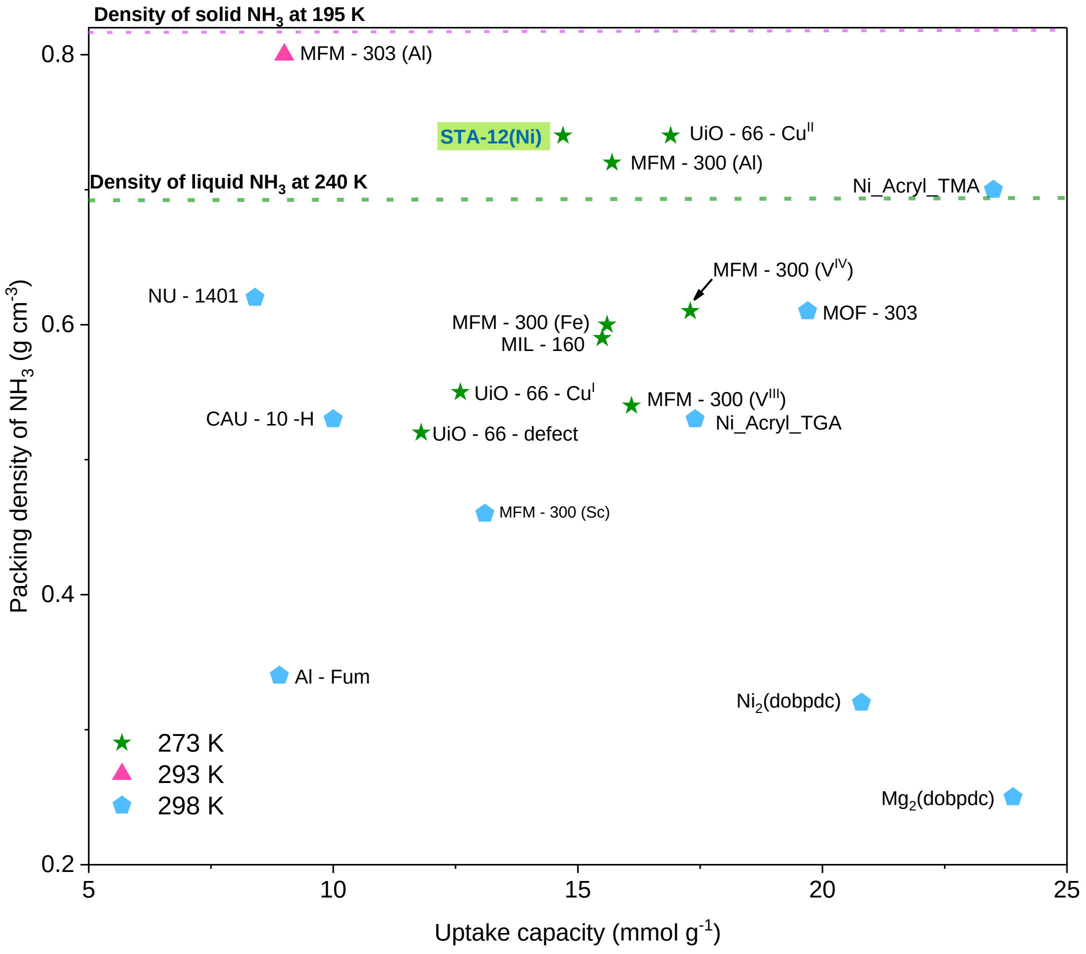

Figure S30: Plot of NH_3 packing densities of various selected state-of-art MOFs against NH_3 uptake capacity. Values are based on theoretical pore volumes or pore volumes obtained through the BET method. The value for STA-12(Ni) was determined using theoretical pore volume calculation. Packing density = Total NH_3 uptake/pore volume.

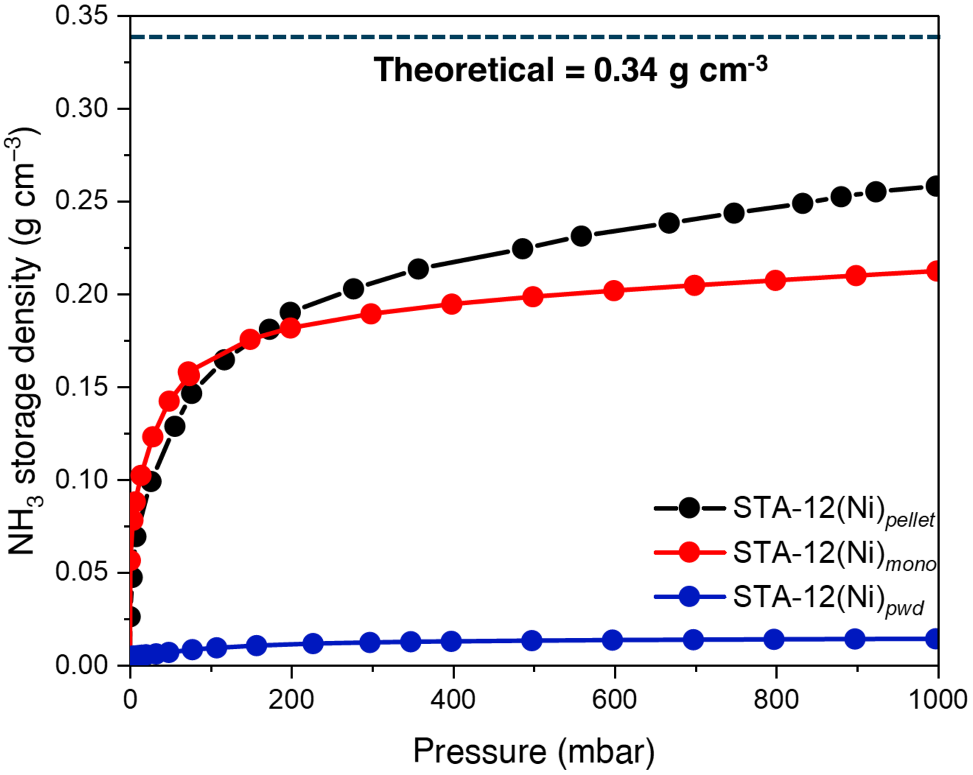

Figure S31: NH_3 storage densities of STA-12(Ni)*pellet* (black), partially hydrated STA-12(Ni)*mono* (red), and STA-12(Ni)*pwd* (blue), based on the isotherms at 273 K. The theoretical storage density (0.34 g cm^{-3}), which is the theoretically maximum NH_3 storage for STA-12(Ni) based on the crystallographic density of activated rhombohedral STA-12(Ni) is also shown in the plot.

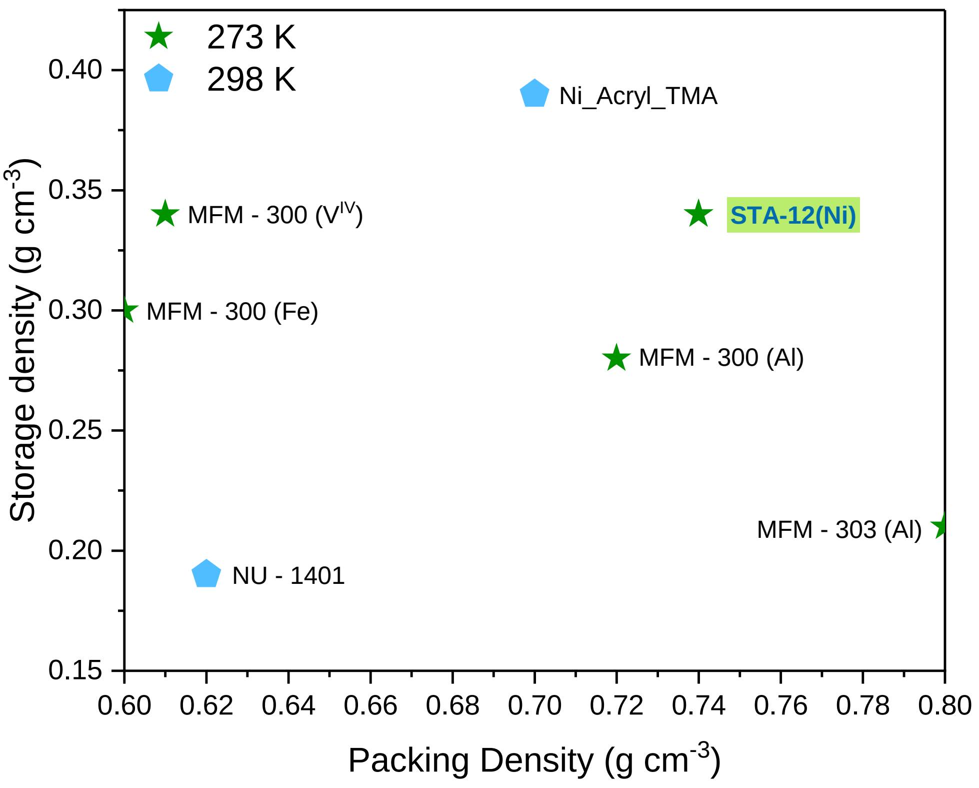

Figure S32: NH_3 packing densities *vs.* storage densities of selected state-of-art MOFs.

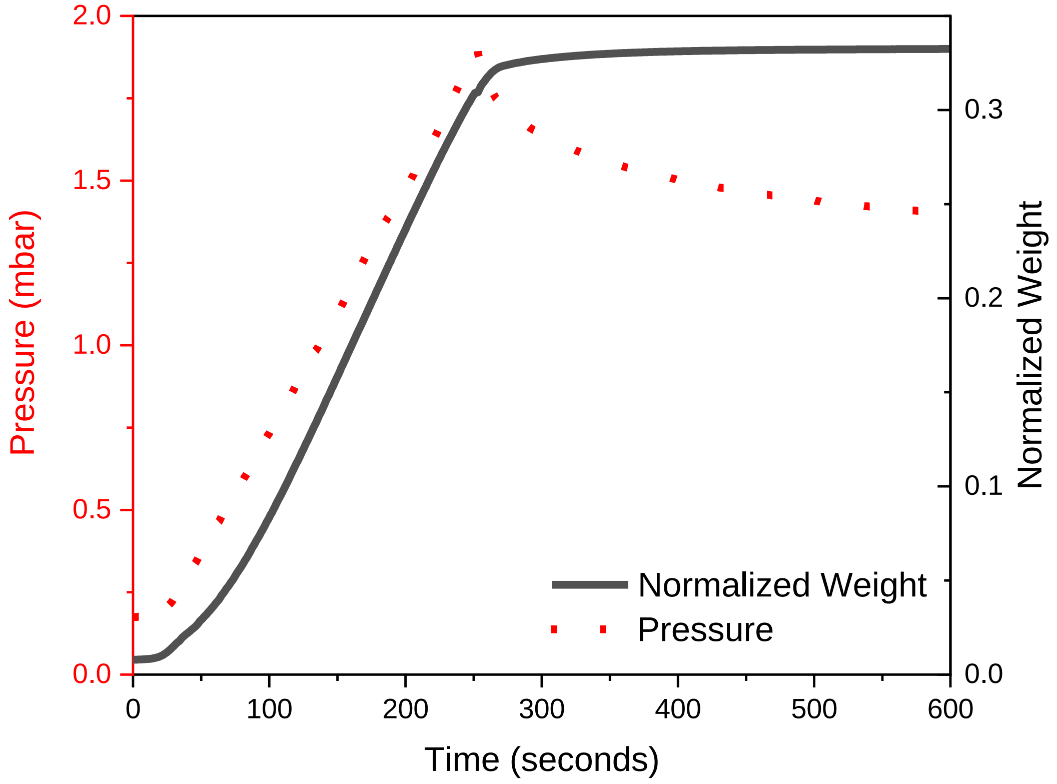

Figure S33: Change in weight and pressure during NH_3 sorption under static conditions at 298 K. At 2 mbar STA-12(Ni) adsorbs approximately 33% of its maximum NH_3 uptake capacity.

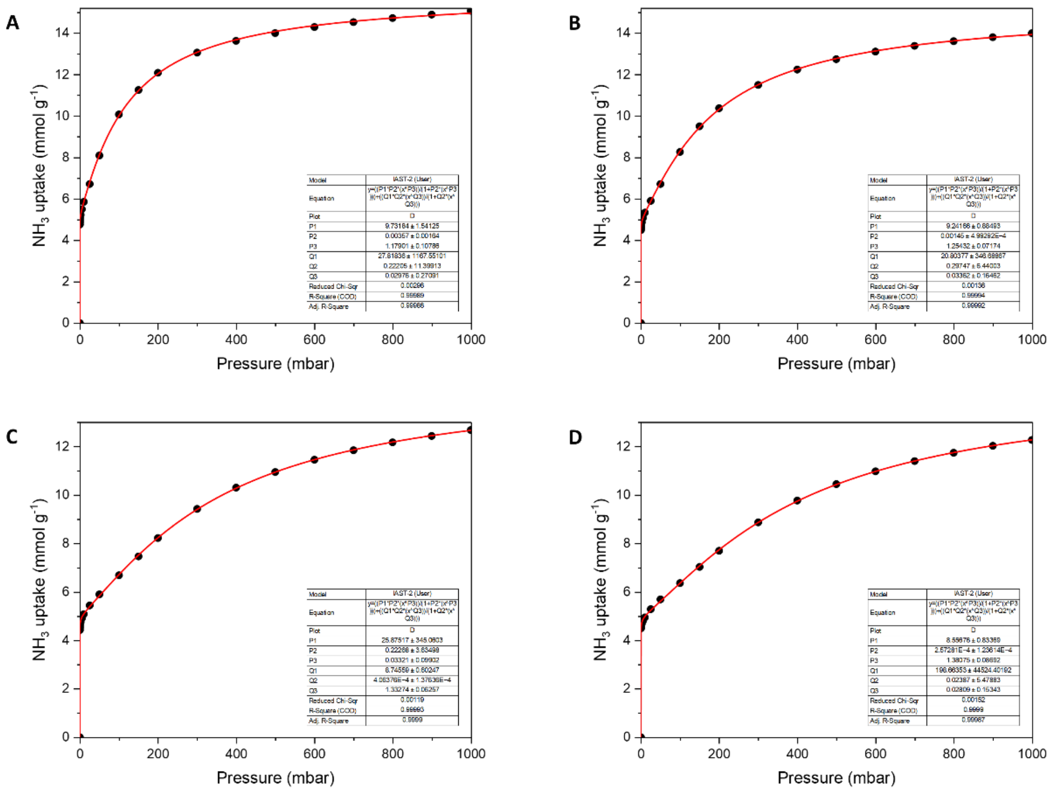

Figure S34: Fittings of isotherms at 273, 283, 298 and 302 K for NH_3 in STA-12(Ni) up to 1 bar using the Dual-site Langmuir Freundlich (DSLF) model.

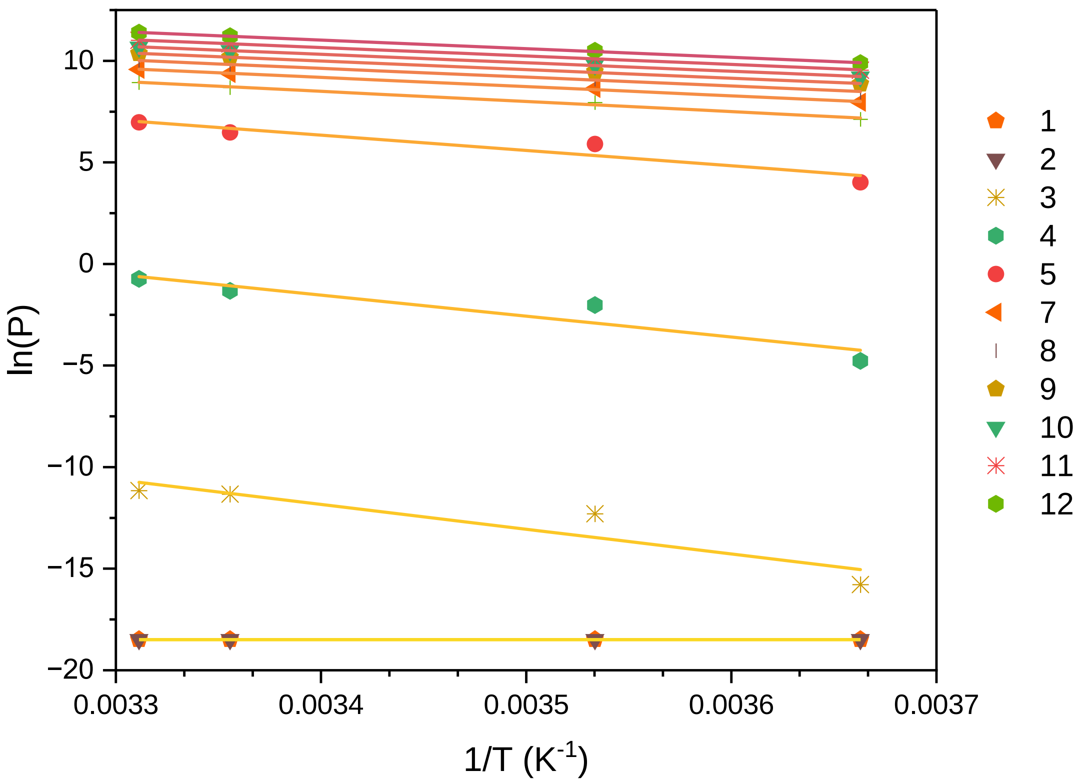

Figure S35: Van't Hoff linear fittings for NH_3 uptake in STA-12(Ni). Each symbol represents a NH_3 loading with the number x referring to x mmol g^{-1}.

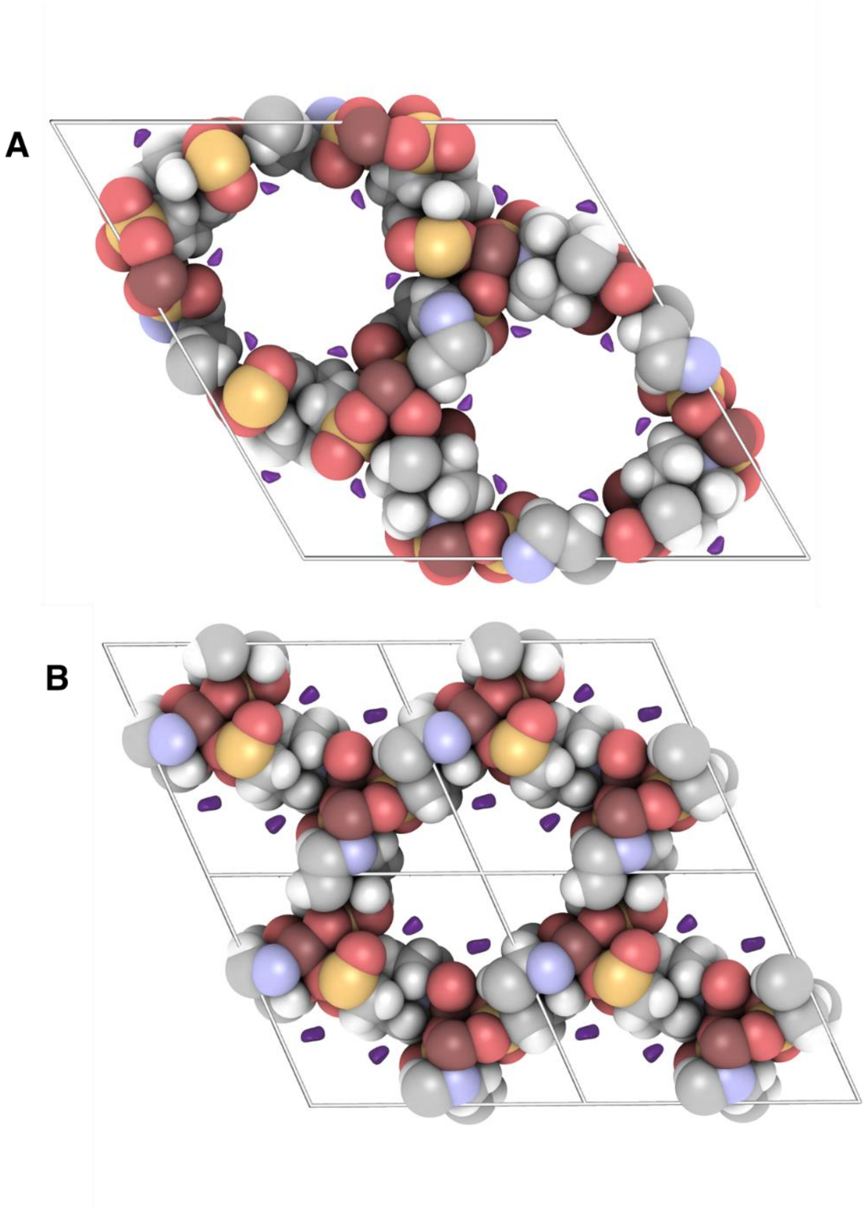

Figure S36: View of adsorption sites of STA-12(Ni) obtained by neutron powder diffraction in (**A**) rhombohedral and (**B**) triclinic forms; the rhombohedral framework has a higher number of accessible adsorption sites compared to the triclinic framework.

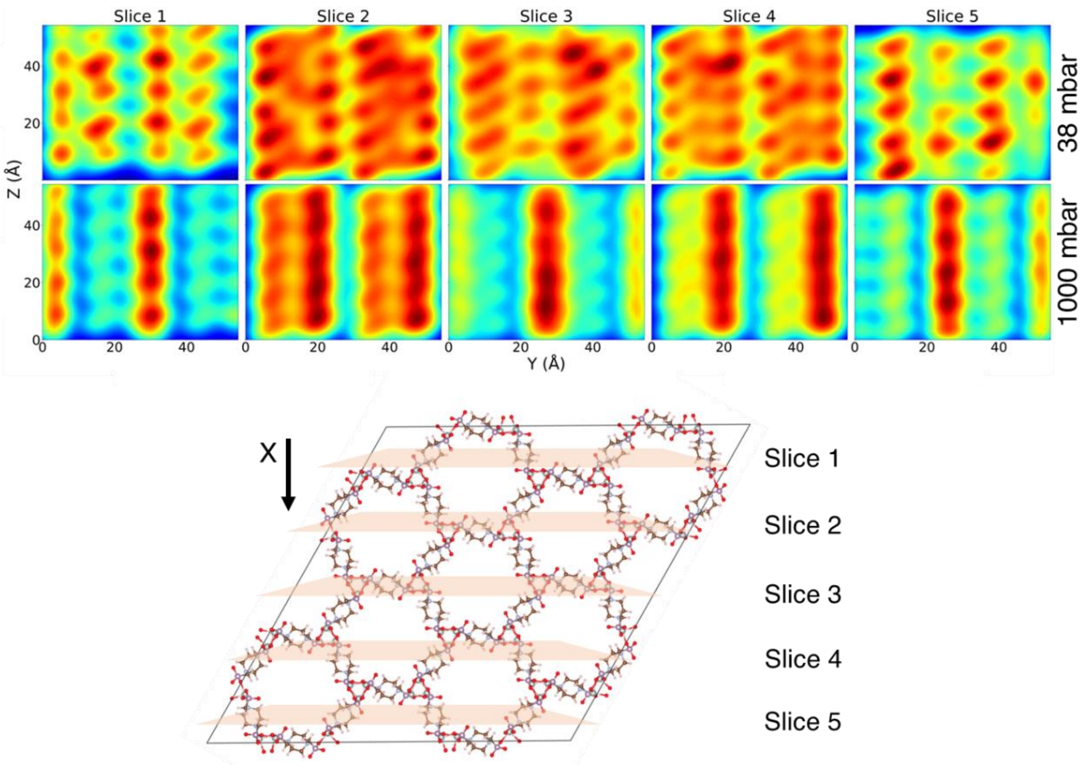

Figure S37: Calculated occupational profiles for NH_3 obtained by molecular simulation of NH_3 adsorption at 38 mbar (top) and 1000 mbar (bottom) viewed down the *c* axis; dark red and dark blue represent high and low densities of adsorbed NH_3, respectively, at each pressure. The view at the bottom shows the corresponding slice through the volume of the framework.

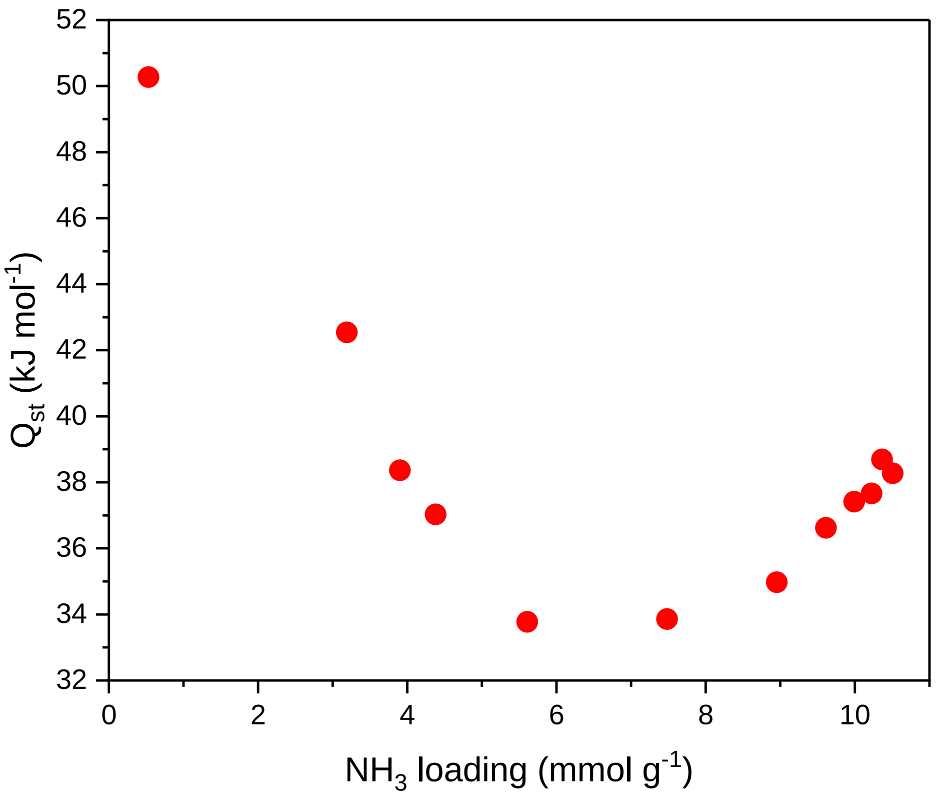

Figure S38: Calculated isosteric heat of adsorption of NH_3 at different loadings in STA-12(Ni) at 298 K. The value (*ca.* 38 kJ mol^{-1}) is consistent with the experimental data (*ca.* 40 kJ mol^{-1} at similar NH_3 loadings).

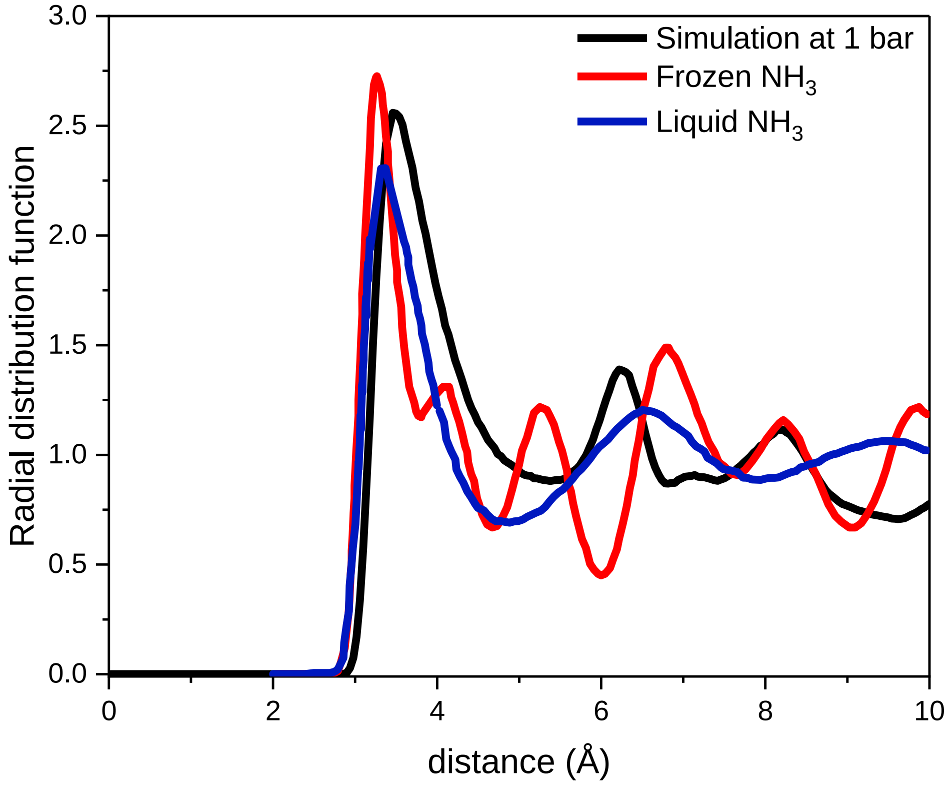

Figure S39: Simulated radial distribution function (RDF) (black) of NH_3 loaded in STA-12(Ni) (rhombohedral framework) for different $N_{NH3}\ldots N_{NH3}$ distances. The RDF of liquid NH_3 (blue) and electro-frozen NH_3 (red) is also shown, indicating that the NH_3 within the pore interior is ordered and in a phase between solid and liquid NH_3.

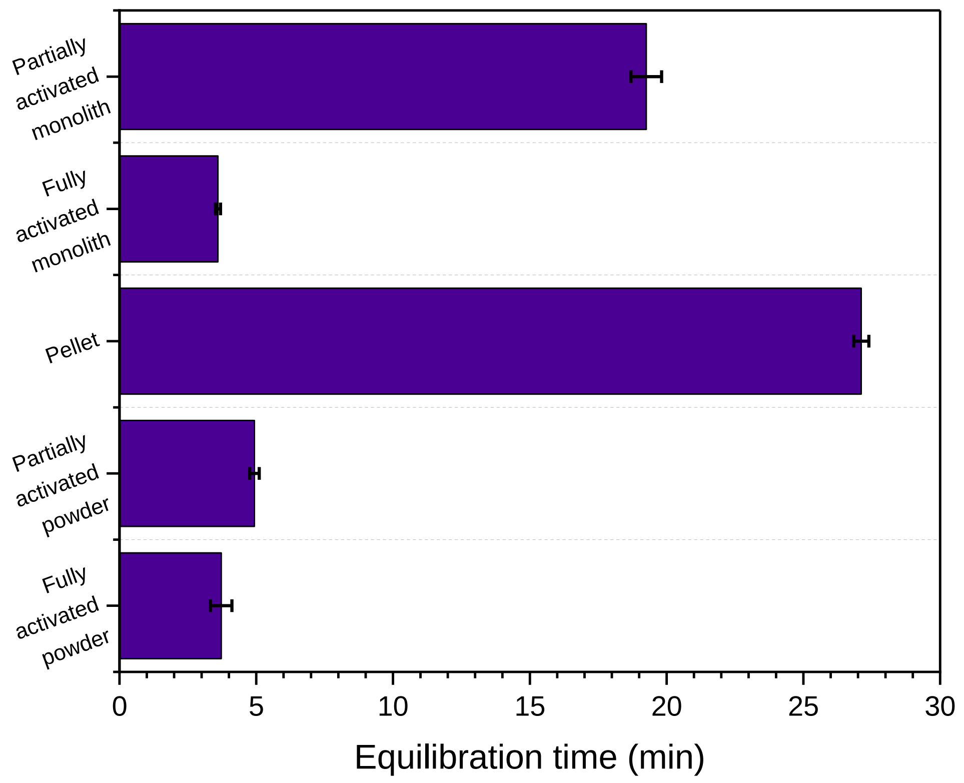

Figure S40: Equilibration time for STA-12(Ni) upon a dosing of 2 mbar of NH_3.

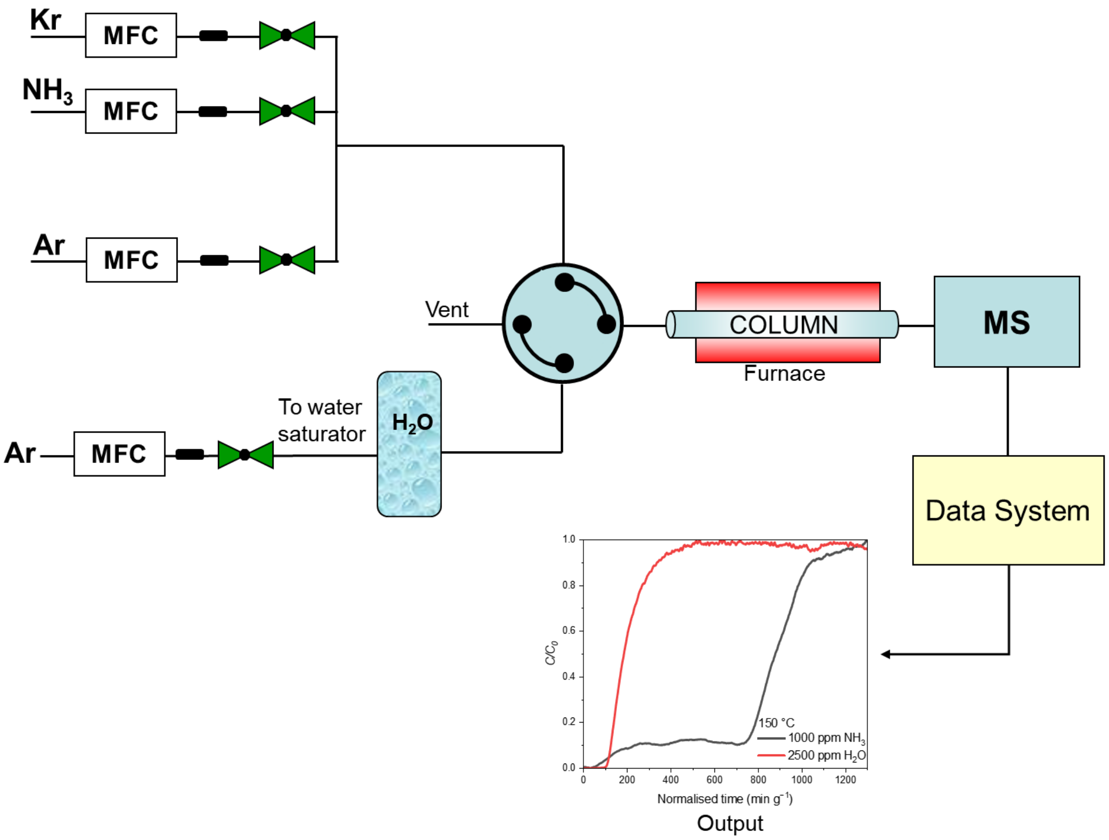

Figure S41: Schematic of breakthrough setup for NH_3. For dry NH_3 breakthrough at 25 °C, the water saturator was not used. Krypton was used as an internal standard.

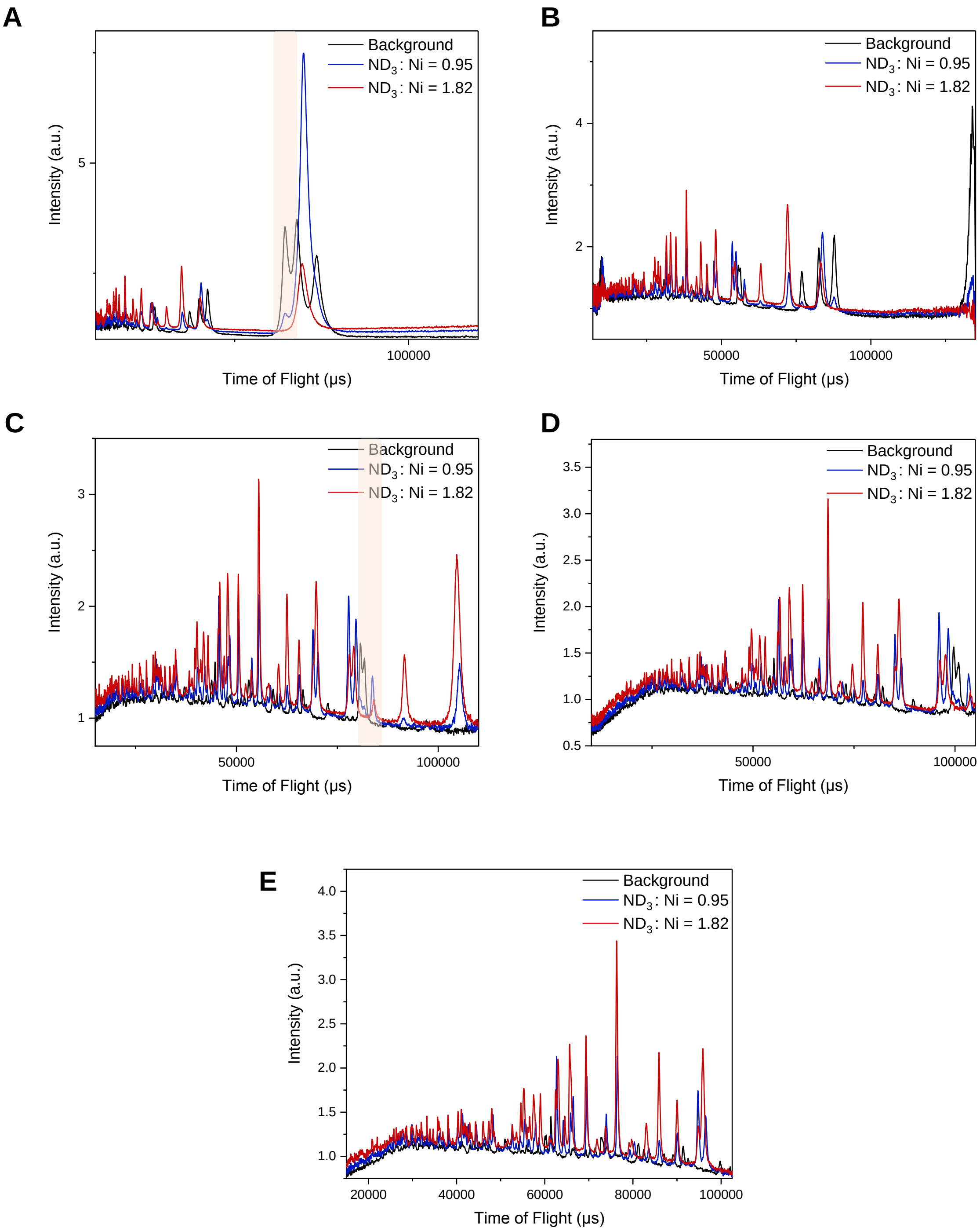

Figure S42: Neutron powder diffraction (NPD) data banks (**A**) 1–10, (**B**) 2–9, (**C**) 3–8, (**D**) 4–7 and (**E**) 5–6 for STA-12(Ni)$_{act}$ (black), ND_3@STA-12(Ni) (ND_3:Ni = 0.95; blue and ND_3:Ni = 1.82; red). Change of spectral pattern signifies a significant structural transformation upon ND_3 loading to the bare MOF. Presence of bare MOF at low loading is indicated in (**A**) and (**C**). The respective DDT1 values are 4990, 10405, 15099, 18652 and 20762. The 2θ values of the banks are 27.081, 58.330, 90.000, 121.660 and 152.827 °.

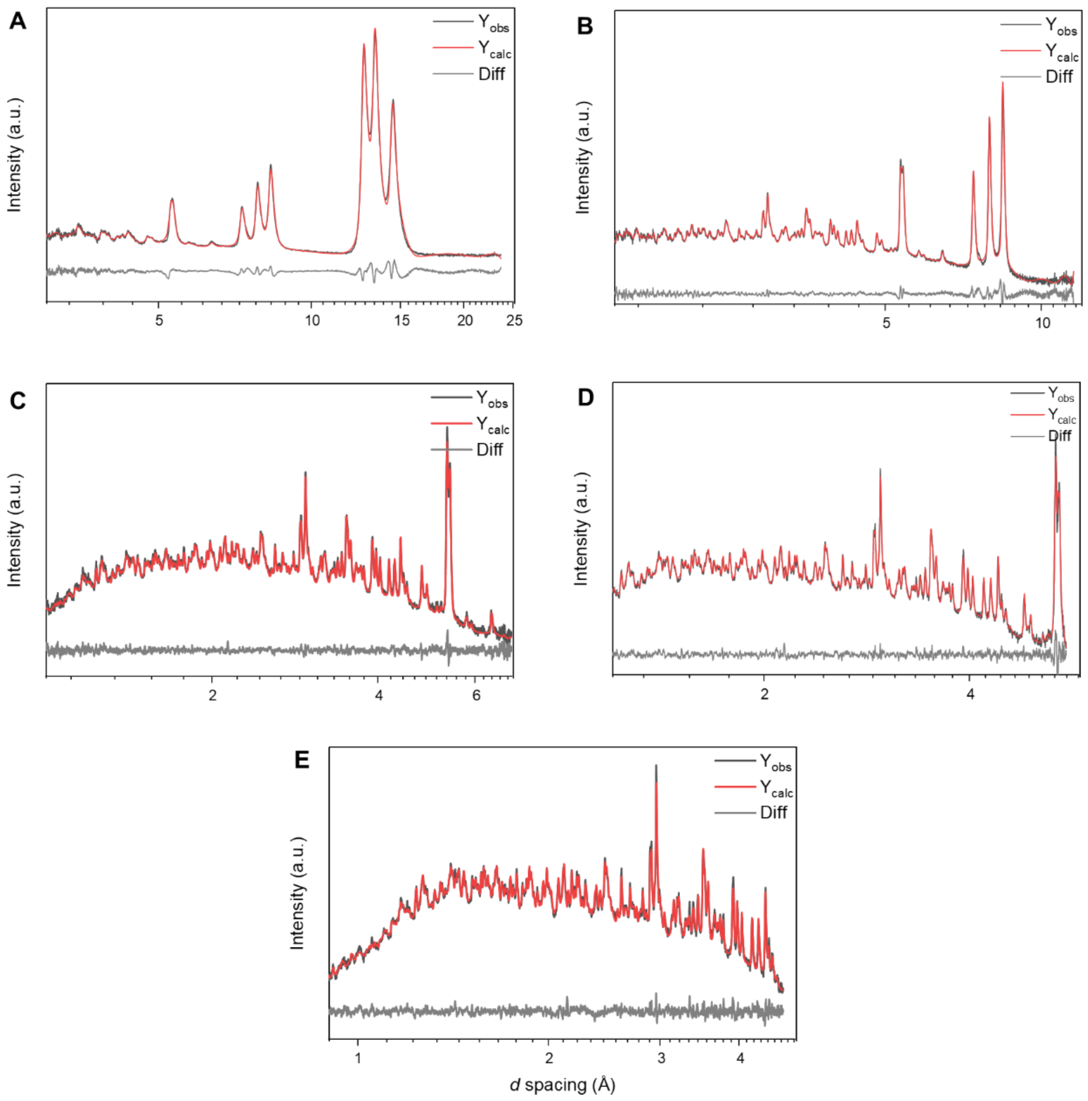

Figure S43: Neutron diffraction patterns and Rietveld refinement plots for bare STA-12(Ni); banks 1 (**A**) to 5 (**E**).

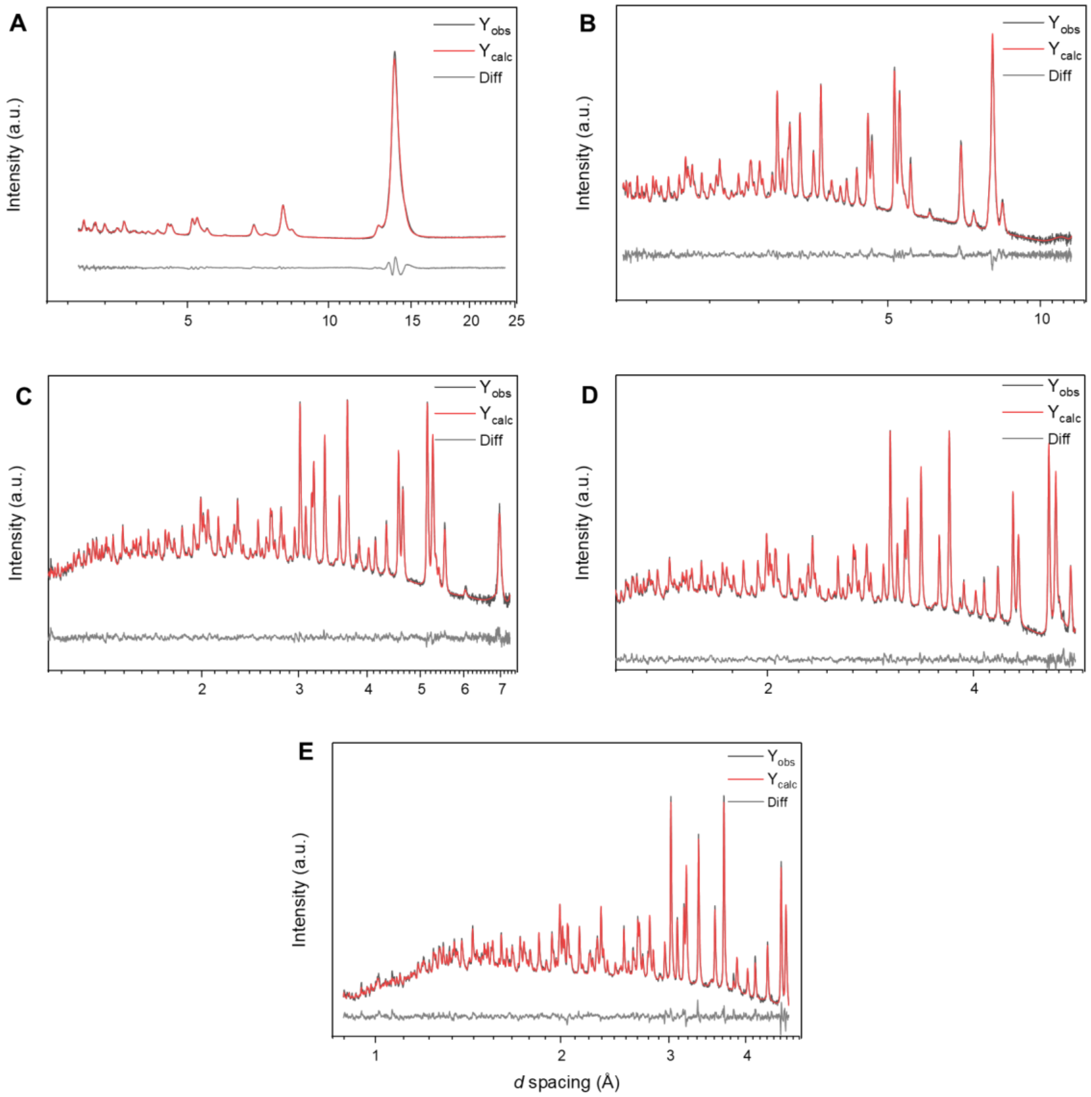

Figure S44: Neutron diffraction patterns and Rietveld refinement for STA-12(Ni).$(ND_3)_{0.95}$ (low ND_3 loading); banks 1 (**A**) to 5 (**E**).

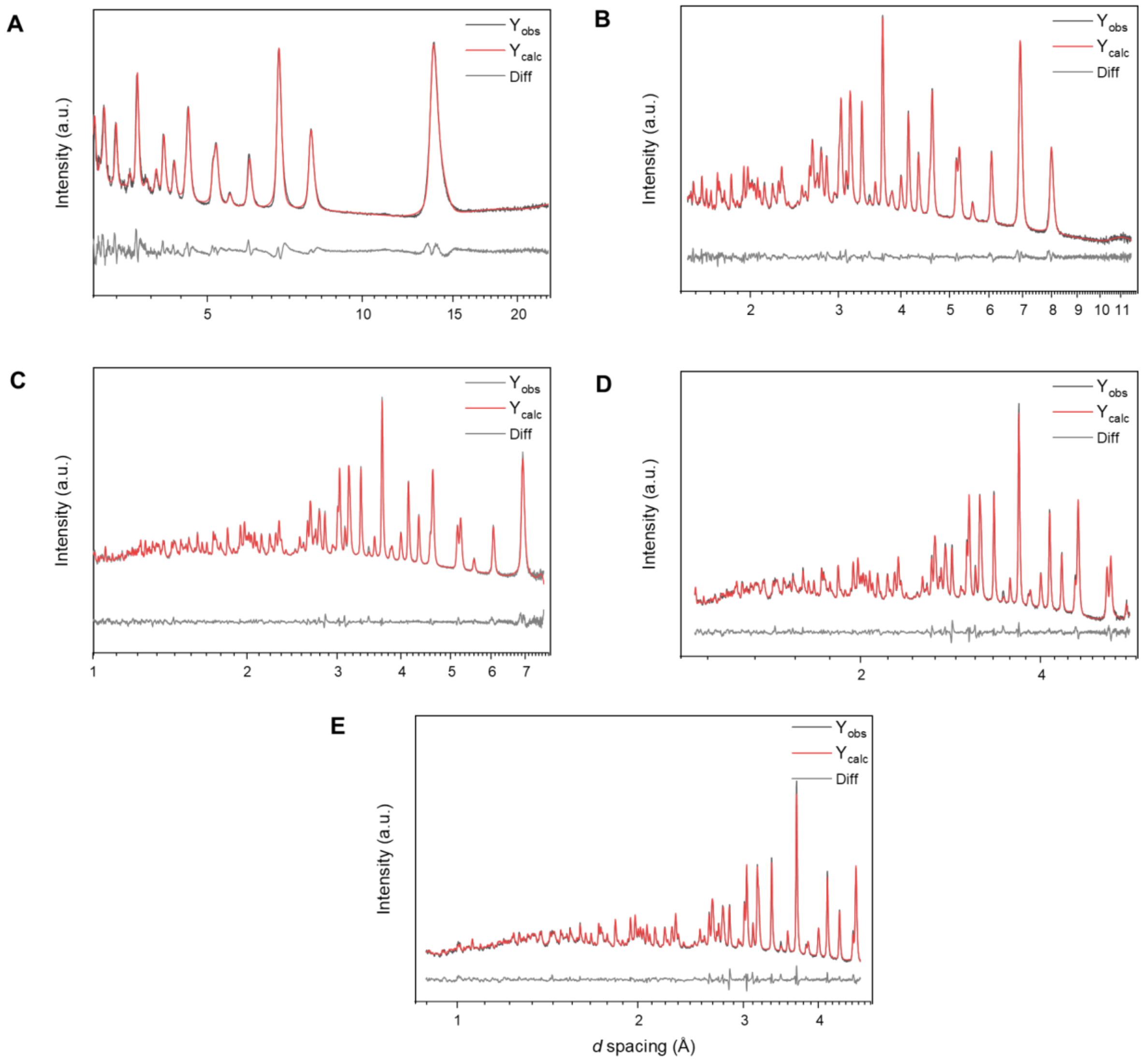

Figure S45: Neutron diffraction patterns and Rietveld refinement plots for STA-12–(Ni).$(ND_3)_{1.82}$ (high ND_3 loading); banks 1 (**A**) to 5 (**E**).

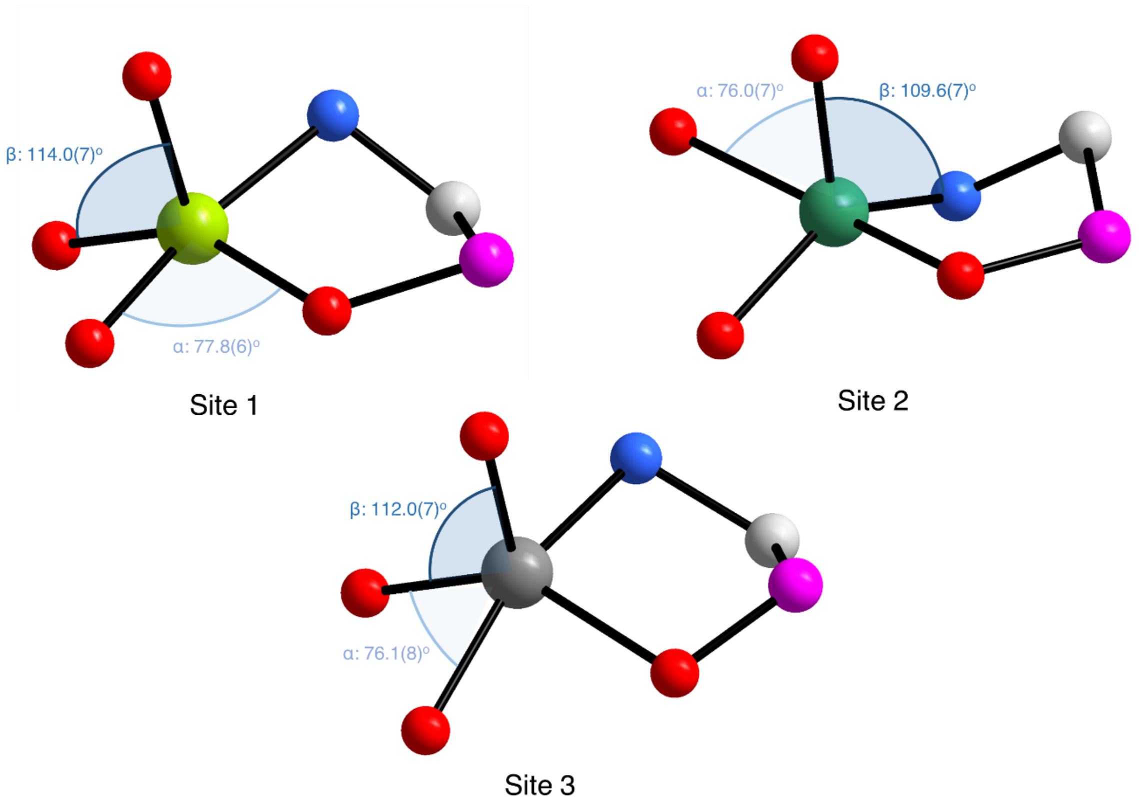

Figure S46: Coordination sites of the three crystallographically distinct Ni^{II} sites (site 1 to 3) of the triclinic framework; the three sites are closest to trigonal pyramidal geometry using the geometry index for a five–coordinate complex (τ_5); τ_5 for sites 1, 2 and 3 are 0.60, 0.56 and 0.60, respectively. O = red, N = blue, C = white, P = pink. Ni^{II} atoms at sites 1 to 3 are depicted as lime, sea green, and grey, respectively.

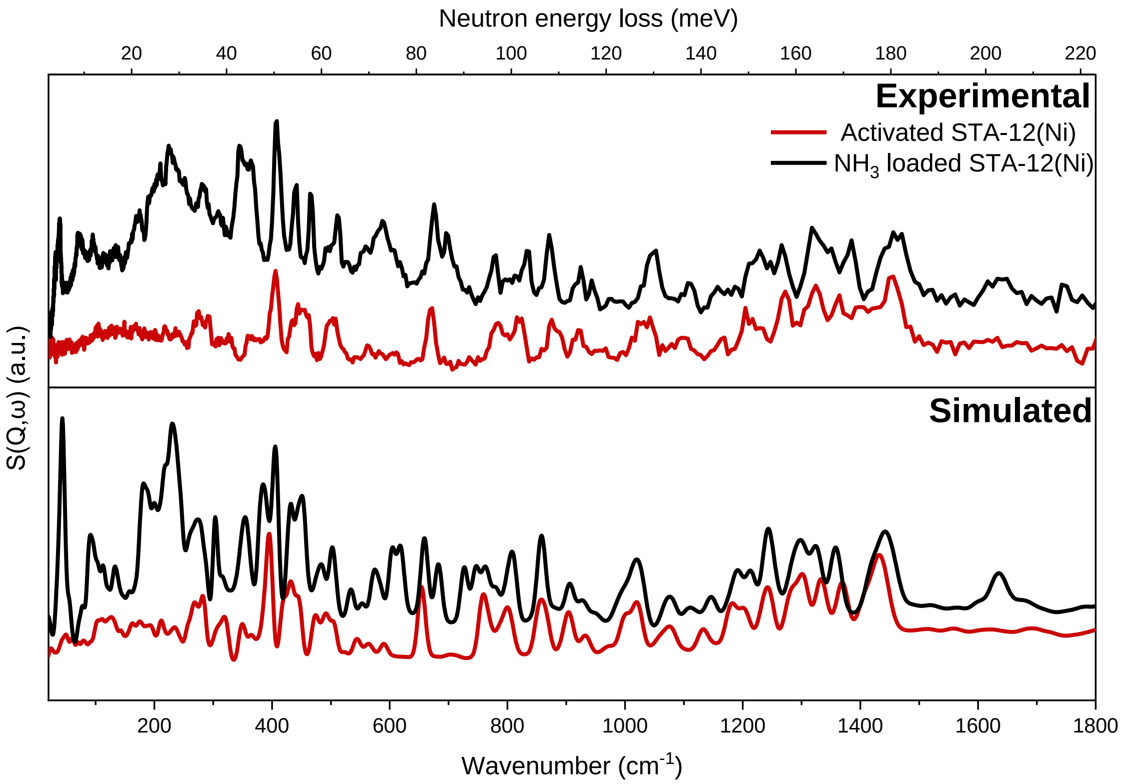

Figure S47: Experimental (top) and simulated (bottom) of bare (red) and NH_3-loaded (black) STA-12(Ni). Slight deviations between experimental and simulated data are due to the approximations and simplifications employed in the simulation from the electronic structure and the van der Waals interactions to the crystal structure. A detailed analysis of the INS spectra is available in Table S7.

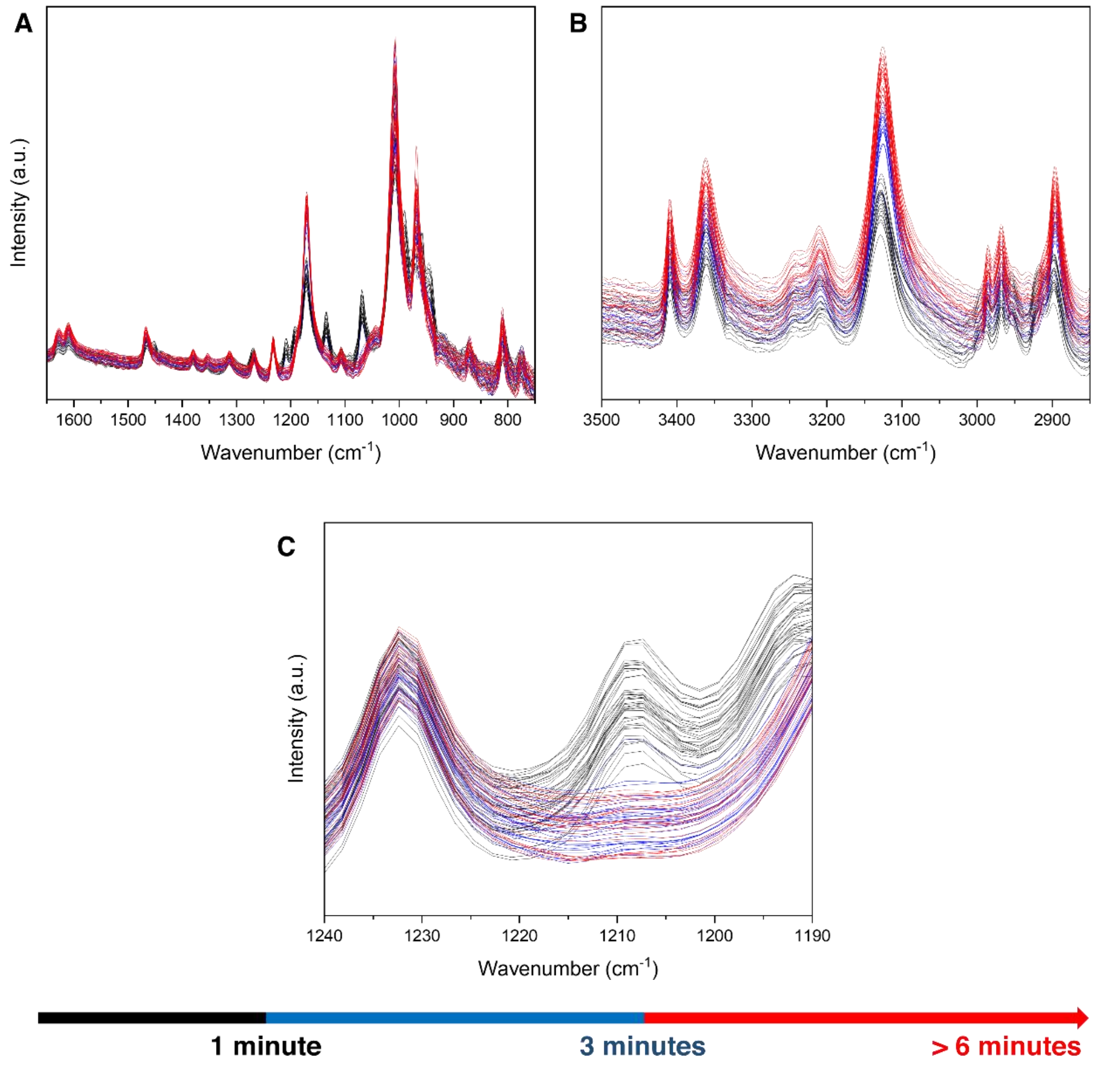

Figure S48: Rapid IR spectra of STA-12(Ni) obtained under a 100 mL flow of 1% NH_3 diluted in N_2 at 120 °C, with (**A**) highlighting the changes in the $[CPO_3]^-$ vibrational modes and the appearance of the N-H degenerate deformation modes (δ_a) at *ca.* 1610 (chemisorbed) and 1630 cm^{-1} (physisorbed). (**B**) shows the antisymmetric (v_a) and symmetric (v_s) modes of adsorbed NH_3, and highlights changes at the C-H stretching modes; here, the C-H vibrational modes split into two main bands with increased NH_3 loading, consistent with the data obtained at 25 °C, confirming that the framework has undergone the structural transition to rhombohedral symmetry. The band associated for the P=O stretch at *ca.* 1207 cm^{-1} (**C**) also disappears upon increasing NH_3 loading. The colour scheme indicates the time at which the spectra were obtained from the start of flow.

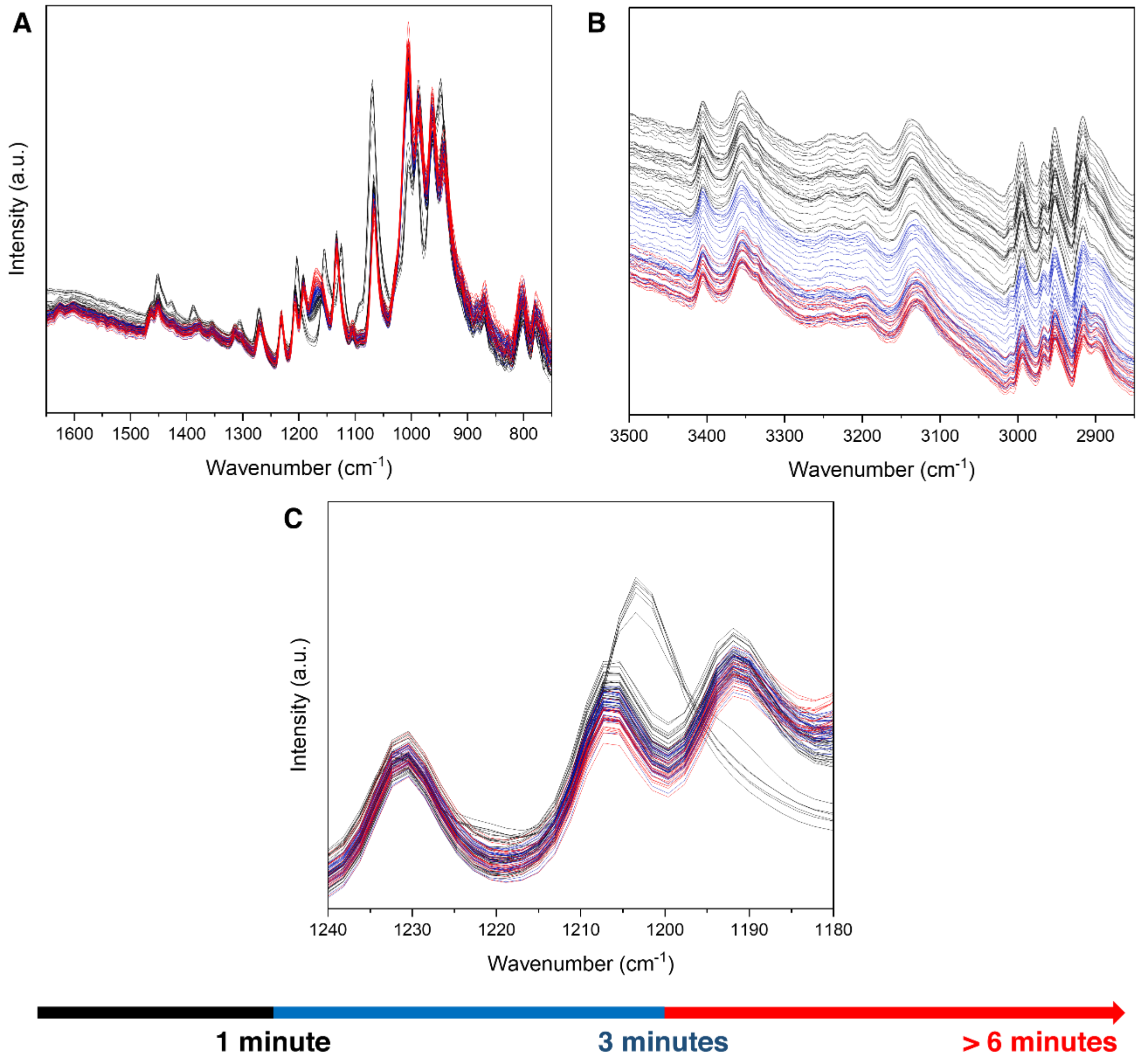

Figure S49: Rapid IR spectra of STA-12(Ni) obtained under a 100 mL flow of 1% NH_3 diluted in N_2 at 180 °C, with (**A**) highlighting the changes in the $[CPO_3]^-$ vibrational modes and the appearance of the N-H degenerate deformation modes (δ_a) at *ca.* 1610 (chemisorbed) and 1630 cm^{-1} (physisorbed). (**B**) shows the presence of the antisymmetric (ν_a) and symmetric (ν_s) modes of adsorbed NH_3, and highlights changes at the C-H stretching modes. The band associated for the P=O stretch at *ca.* 1203 cm^{-1} (**C**) also splits into two bands (1205 and 1191 cm^{-1}) with increasing NH_3 loading. The colour scheme indicates the time at which the spectra were obtained from the point of start of flow.

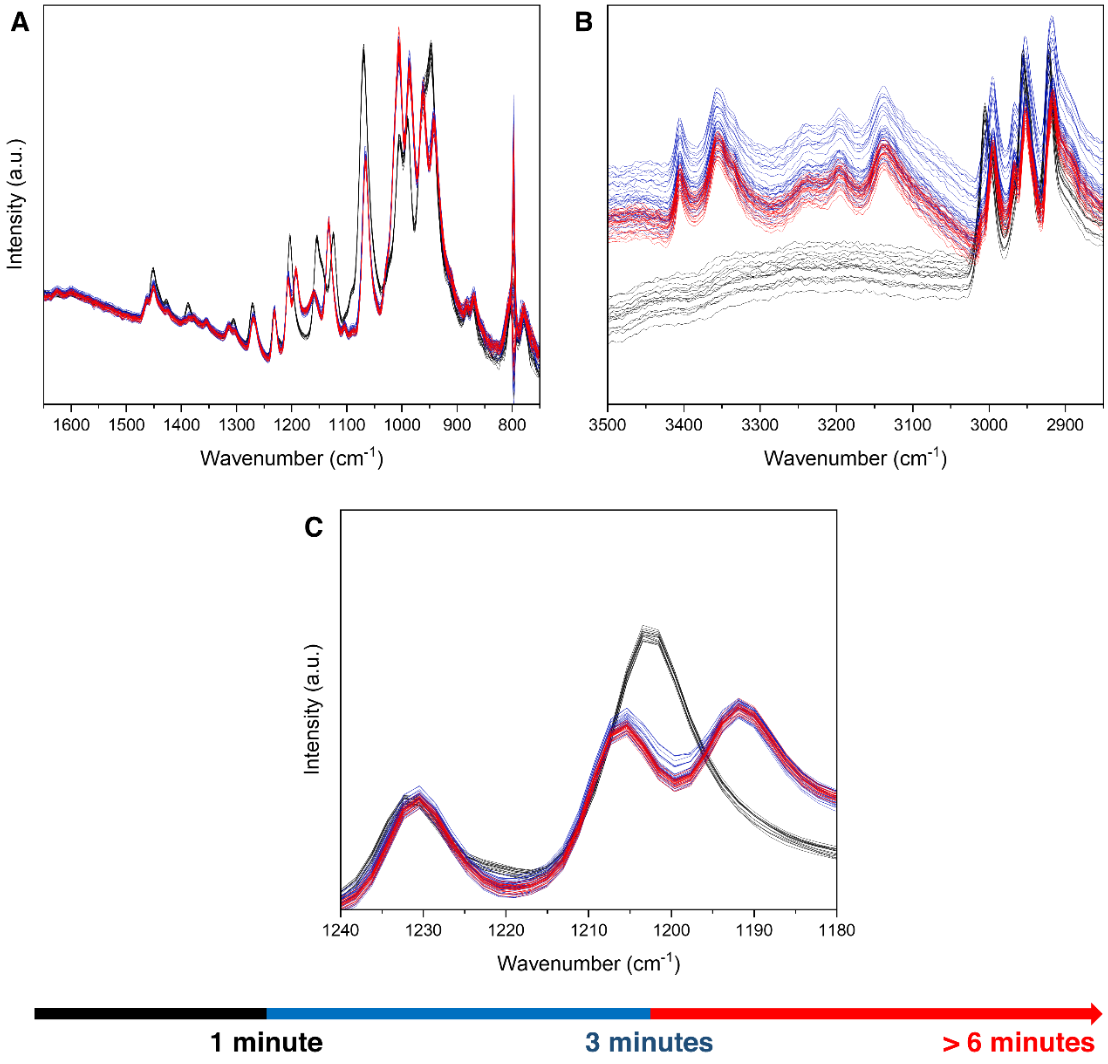

Figure S50: Rapid IR spectra of STA-12(Ni) obtained under a 100 mL flow of 1% NH_3 diluted in N_2 at 200 °C, with (**A**) highlighting the changes in the $[CPO_3]^-$ vibrational modes and the appearance of the N-H degenerate deformation modes (δ_a) at *ca.* 1610 (chemisorbed) and 1630 cm^{-1} (physisorbed). (**B**) shows the presence of the antisymmetric (ν_a) and symmetric (ν_s) modes of adsorbed NH_3, and highlights changes at the C-H stretching modes, similar to the spectra obtained at 180 °C. The band associated for the P=O stretch at *ca.* 1203 cm^{-1} (**C**) also splits into two bands (1206 and 1191 cm^{-1}) with increased NH_3 loading. The colour scheme indicates the time at which the spectra were obtained from the point of start of flow.

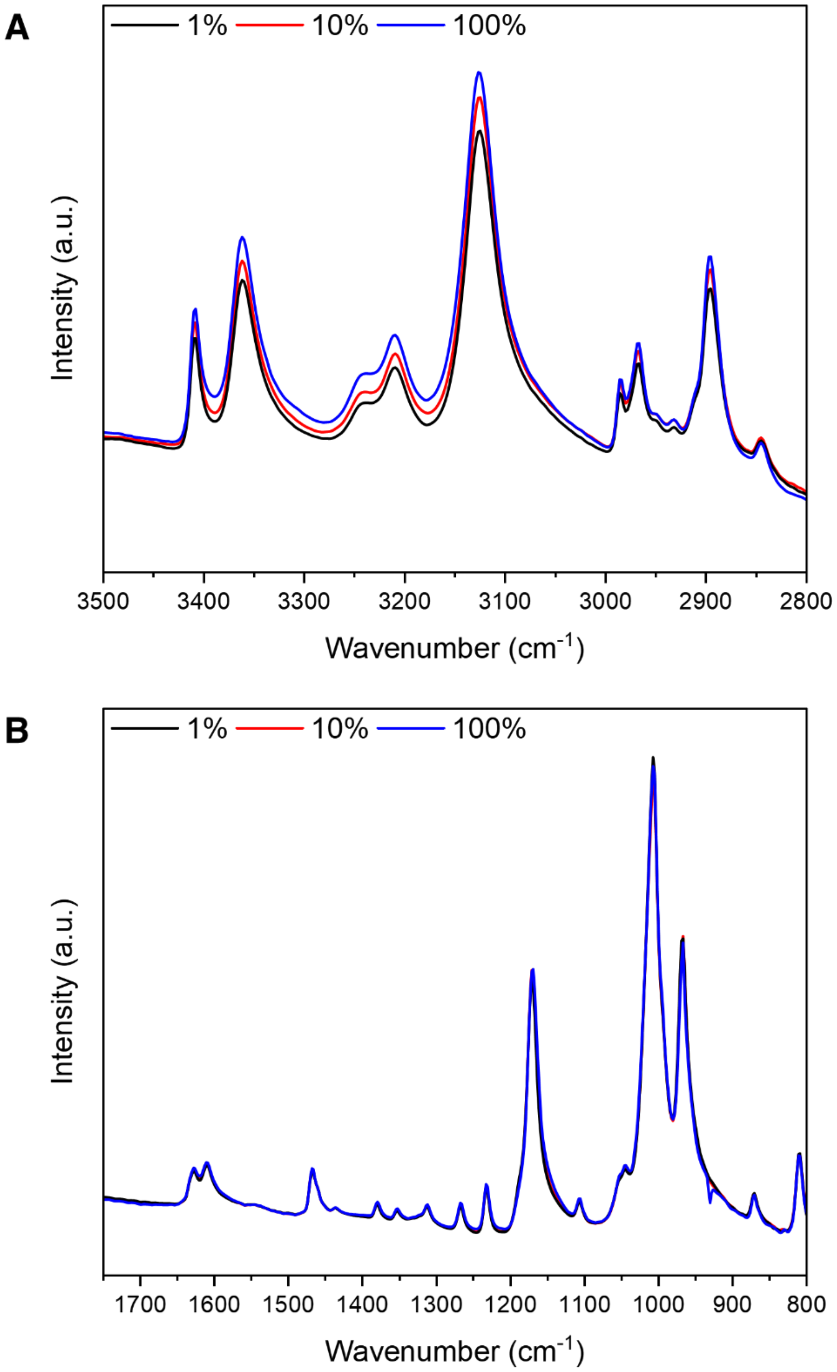

Figure S51: *In situ* IR spectra of STA-12(Ni) with 1% (black), 10% (red) and 100% (blue) NH_3 under a 100 mL flow of NH_3 diluted in N_2 at 120 °C. (**A**) shows the bands from 3500 to 2800 cm^{-1} and (**B**) highlights the bands between 1750 to 800 cm^{-1}. At this temperature, even at 1%, the material has transformed into rhombohedral symmetry, with all bands involving both chemisorbed and physisorbed NH_3 present, comparable to the spectra obtained at 30 °C.

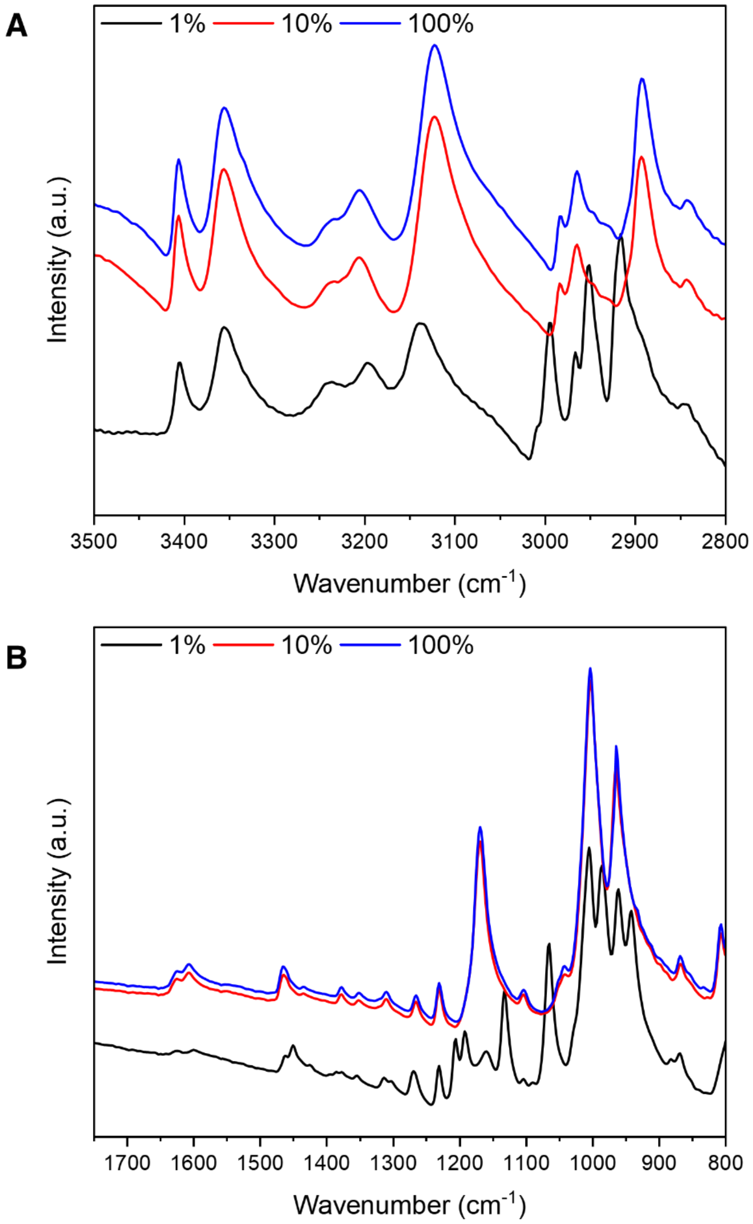

Figure S52: *In situ* IR spectra of STA-12(Ni) with 1% (black), 10% (red) and 100% (blue) NH_3 under a 100 mL flow of NH_3 diluted in N_2 at 200 °C. (**A**) shows the bands from 3500 to 2800 cm^{-1} and (**B**) highlights the bands between 1750 to 800 cm^{-1}. At 10% NH_3 has the material fully transformed into rhombohedral symmetry.

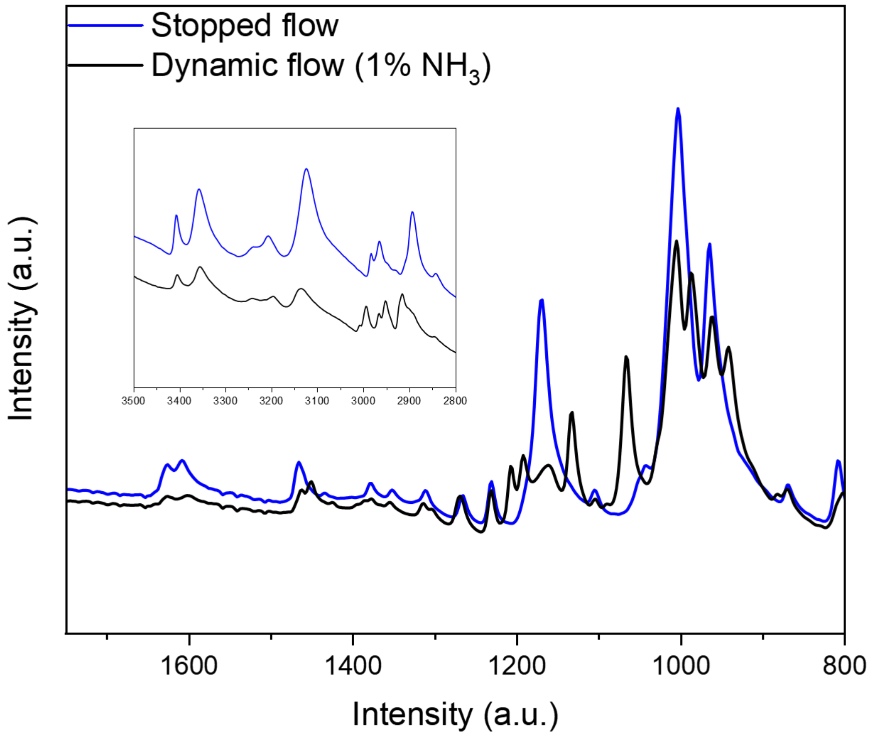

Figure S53: *In situ* IR spectra of STA-12(Ni) at 180 °C under a dynamic flow (black) of 1% NH_3 diluted in N_2. The blue spectra were obtained with the N_2 and NH_3 flow stopped.

Supplementary Tables

Table S1: Selected state-of-the-art phosphonate MOFs reported for various toxic gas and vapour capture and storage. Materials for which porosity was confirmed through N_2 adsorption studies only or by computational calculations are not reported below.

	**Gas/vapour**	**Notes**	**Ref**
STA-12(Ni)	CO_2 (2.5 mmol g^{-1}, 298 K, 1 bar)<br>CH_4 (0.25 mmol g^{-1}, 298 K, 1 bar)	Measurements were carried out on dehydrated triclinic structure.	1
STA-12 (Mg and Co)	CO_2 (*ca.* 8.5 and 6.0 mmol g^{-1} respectively, at 195 K, 0.4 bar)	Measurements were carried out on dehydrated triclinic structure for STA-12 Co.	2
CAU–14	Water vapour (*ca.* 16 mmol g^{-1} at 298 K, 1 bar)	Copper MOF with open metal sites.	32
CAU–25	CO_2 (0.40 mmol g^{-1} at 298 K, 1 bar)<br>Water vapour (7.3 mmol g^{-1} at 298 K, 1 bar)	Zinc MOF, did not show porosity towards N_2.	33
Al–CAU–60	HCl (6.9 mmol g^{-1})	HCl absorbed through protonated phosphonate linker.	34
MIL–91 (Al)	CO_2 (3.0 mmol g^{-1} at 294 K, 1 bar)<br>CH_4 (1.7 mmol g^{-1} at 294 K, 40 bar)<br>C_2H_6 (1.1 mmol g^{-1} at 294 K, 10 bar)<br>C_3H_8 (0.8 mmol g^{-1} at 294 K, 10 bar)	Microporous aluminium MOF with high selectivity towards CO_2 over hydrocarbons.	35
CALF–25	CO_2 (*ca.* 4.0 mmol g^{-1} at 195 K, 0.8 bar)<br>CH_4 (*ca.* 0.28 mmol g^{-1} at 278 K, 1 bar)	A phosphonate monoester linker–based barium MOF.	36

Table S2: Elemental analytical data for the powder, monolithic and pelletised forms of STA-12(Ni). Slight discrepancies between calculated and experimental values are due to slightly different amounts of guest molecules present.

	C	H	N	Ni^{II}	P	K
STA-12(Ni) (expected)	13.66	4.58	5.31	22.25	11.74	0
STA-12(Ni)$_{pwd}$	13.38	5.04	5.05	21.65	11.36	0
STA-12(Ni)$_{mono}$	13.57	5.12	5.22	20.47	10.98	2.72
STA-12(Ni)$_{pellet}$	13.24	5.31	4.99	21.71	11.29	0

Table S3: Crystallographic data obtained from the refinement of neutron diffraction patterns for activated and ND_3-loaded STA-12(Ni).

	bare [STA-12 (Ni)]	[STA-12 (Ni)].$(ND_3)_{0.95}$	[STA-12 (Ni)].$(ND_3)_{1.82}$
Formula	$C_9H_{18}N_3Ni_3O_9P_3$	$C_3H_6D_{2.88}N_{1.96}NiO_3P$	$C_3D_{5.45}H_6N_{2.82}NiO_3P$
Formula weight	581.24	212.99	230.16
Crystal system	Triclinic	Rhombohedral	Rhombohedral
Space group	$P\bar{1}$	$R\bar{3}$	$R\bar{3}$
a (Å)	6.0201(6)	27.8707(11)	27.7016(12)
b (Å)	14.8900(15)	27.8707(11)	27.7016(12)
c (Å)	16.1125(16)	6.2322(3)	6.2726(3)
α (°)	112.7316(19)	90	90
β (°)	95.721(3)	90	90
γ (°)	96.485(3)	120	120
Volume (Å^3)	1307.3(2)	4192.5(4)	4168.6(4)
ρ (calc) (g cm^{-3})	1.477	1.518	1.6503
Z	2	18	18
Radiation type	Neutron	Neutron	Neutron
Scan method	Time of flight	Time of flight	Time of flight
R_{exp} (%)	0.45495	0.44431	0.43250
R_{wp} (%)	0.95765	1.06881	1.29296
R_p (%)	0.83006	0.93023	1.07421
GoF (χ^2)	2.10493	2.40550	2.98951

Table S4: Atomic positions for atoms in activated STA-12(Ni).

	x	*y*	*z*	Occupancy	B_{iso}/Å^{-2}
Ni1	0.8239(14)	0.3218(5)	0.7787(5)	1	2.07(16)
Ni2	0.0521(15)	0.2454(5)	0.6109(5)	1	2.07(16)
Ni3	0.4630(15)	0.4449(6)	0.6812(6)	1	2.07(16)
N1	–0.0094(16)	0.4186(6)	0.9086(6)	1	2.00(18)
P1	0.361(3)	0.3536(12)	0.8147(14)	1	4.6(4)
O11	0.516(3)	0.2728(10)	0.8173(8)	1	2.56(17)
O12	0.117(3)	0.2929(10)	0.7356(9)	1	2.56(17)
O13	0.421(2)	0.4471(11)	0.8050(11)	1	2.56(17)
C11	0.206(2)	0.3701(8)	0.9148(8)	1	2.00(10)
C12	0.0444(19)	0.5194(10)	0.9201(8)	1	2.00(10)
C13	–0.1409(18)	0.4141(8)	0.9884(9)	1	2.00(10)
H11a	0.294(2)	0.4327(8)	0.9769(9)	1	2.40(13)
H11b	0.185(2)	0.3018(9)	0.9267(10)	1	2.40(13)
H12a	0.148(2)	0.5166(10)	0.8668(9)	1	2.40(13)
H12b	–0.132(2)	0.5230(10)	0.8981(9)	1	2.40(13)
H13a	–0.308(2)	0.4357(9)	0.9804(9)	1	2.40(13)
H13b	–0.160(2)	0.3359(10)	0.9777(9)	1	2.40(13)
N2	0.0752(16)	0.0999(6)	0.5557(6)	1	2.00(18)
P2	0.523(3)	0.2060(13)	0.5835(14)	1	4.6(4)
O21	0.368(3)	0.2963(11)	0.5962(12)	1	2.56(17)
O22	0.727(2)	0.2309(9)	0.6528(9)	1	2.56(17)
O23	0.535(3)	0.1762(9)	0.4711(9)	1	2.56(17)
C21	0.339(2)	0.0972(8)	0.5975(7)	1	2.00(10)
C22	0.0551(16)	0.0730(10)	0.4625(10)	1	2.00(10)

C23	–0.0661(18)	0.0255(8)	0.5930(9)	1	2.00(10)
H21a	0.393(2)	0.0272(9)	0.5569(7)	1	2.40(13)
H21b	0.404(2)	0.1206(9)	0.6703(8)	1	2.40(13)
H22a	–0.1105(19)	0.0957(10)	0.4543(10)	1	2.40(13)
H22b	0.1797(19)	0.1090(11)	0.4348(10)	1	2.40(13)
H23a	–0.235(2)	0.0460(8)	0.5863(9)	1	2.40(13)
H23b	0.030(2)	0.0713(9)	0.6610(10)	1	2.40(13)
N3	0.5620(14)	0.4955(6)	0.5821(6)	1	2.00(18)
P3	0.939(3)	0.4480(15)	0.6557(13)	1	4.6(4)
O31	0.802(3)	0.4397(11)	0.7338(11)	1	2.56(17)
O32	1.196(3)	0.5126(10)	0.7106(10)	1	2.56(17)
O33	0.979(2)	0.3614(12)	0.5801(11)	1	2.56(17)
C31	0.835(2)	0.5336(8)	0.6219(8)	1	2.00(10)
C32	0.5018(17)	0.4039(8)	0.5079(9)	1	2.00(10)
C33	0.4365(18)	0.5618(8)	0.5823(9)	1	2.00(10)
H31a	0.880(2)	0.5468(8)	0.5626(9)	1	2.40(13)
H31b	0.811(2)	0.6028(9)	0.6762(9)	1	2.40(13)
H32a	0.327(2)	0.3721(8)	0.5071(9)	1	2.40(13)
H32b	0.617(2)	0.3510(9)	0.5059(9)	1	2.40(13)
H33a	0.4540(18)	0.6355(9)	0.6376(9)	1	2.40(13)
H33b	0.262(2)	0.5224(9)	0.5684(10)	1	2.40(13)

Table S5: Atomic positions for atoms in STA-12(Ni).$(ND_3)_{0.95}$.

	x	*y*	*z*	Occupancy	$B_{iso}/Å^{-2}$
Ni	–0.03139(15)	0.35911(15)	0.0526(7)	1	1.76(11)
P1	0.0901(3)	0.3760(3)	–0.1016(14)	1	1.00(16)
O1	0.1447(3)	0.4278(2)	–0.1292(13)	1	2.88(13)
O2	0.0515(3)	0.3826(3)	0.0481(12)	1	2.88(13)
O3	0.0563(3)	0.3430(3)	–0.2883(11)	1	2.88(13)
N1	0.11989(17)	0.34194(19)	0.2811(7)	1	2.55(11)
C1	0.1141(3)	0.2958(3)	0.4159(10)	1	2.65(7)
C2	0.1570(3)	0.2793(2)	0.3609(8)	1	2.65(7)
C3	0.1017(3)	0.3233(2)	0.0541(10)	1	2.65(7)
H1a	0.0718(4)	0.2618(5)	0.400(2)	1	2.91(8)
H1b	0.1187(4)	0.3051(4)	0.5876(18)	1	2.91(8)
H2a	0.1558(5)	0.2672(4)	0.1934(18)	1	2.91(8)
H2b	0.1520(4)	0.2478(4)	0.4760(15)	1	2.91(8)
H3a	0.1348(4)	0.3218(5)	–0.0336(15)	1	2.91(8)
H3b	0.0640(5)	0.2836(5)	0.073(2)	1	2.91(8)
N1_1	–0.1119(2)	0.3356(2)	0.0878(10)	0.960(4)	3.80(13)
D1_1	–0.1202(3)	0.3468(3)	0.2324(12)	0.960(4)	3.80(13)
D2_1	–0.1338(3)	0.3421(3)	–0.0278(11)	0.960(4)	3.80(13)
D3_1	–0.1364(3)	0.2942(3)	0.1061(12)	0.960(4)	3.80(13)

Table S6: Atomic positions for atoms in STA-12(Ni).$(ND_3)_{1.82}$.

	x	*y*	*z*	Occupancy	B_{iso}/Å^{-2}
Ni	–0.03335(16)	0.35939(17)	0.0471(8)	1	2.64(13)
P1	0.0868(3)	0.3723(4)	–0.0890(14)	1	2.4(2)
O1	0.1384(3)	0.4234(3)	–0.1565(11)	1	2.33(14)
O2	0.0545(3)	0.3850(3)	0.0439(12)	1	2.33(14)
O3	0.0534(3)	0.3379(3)	–0.2949(10)	1	2.33(14)
N1	0.11976(19)	0.3421(2)	0.2768(8)	1	2.83(14)
C1	0.1128(3)	0.2953(3)	0.4068(11)	1	3.10(8)
C2	0.1576(3)	0.2766(3)	0.3563(11)	1	3.10(8)
C3	0.1020(3)	0.3243(3)	0.0594(10)	1	3.10(8)
H1a	0.0710(5)	0.2594(6)	0.386(2)	1	3.42(9)
H1b	0.1094(5)	0.3003(5)	0.577(2)	1	3.42(9)
H2a	0.1571(5)	0.2623(5)	0.194(2)	1	3.42(9)
H2b	0.1555(5)	0.2463(6)	0.4716(18)	1	3.42(9)
H3a	0.1338(6)	0.3221(6)	–0.0344(16)	1	3.42(9)
H3b	0.0669(5)	0.2821(5)	0.0491(18)	1	3.42(9)
N1_1	–0.1115(2)	0.3359(2)	0.0938(9)	0.915(6)	2.00(11)
D1_1	–0.1176(3)	0.3484(3)	0.2396(11)	0.915(6)	2.00(11)
D2_1	–0.1358(3)	0.3416(3)	–0.0127(10)	0.915(6)	2.00(11)
D3_1	–0.1364(3)	0.2939(3)	0.1160(10)	0.915(6)	2.00(11)
N1_2	0.3443(3)	0.5394(2)	0.2444(5)	0.674(4)	5.9(2)
D1_2	0.3707(5)	0.5807(3)	0.273(3)	0.674(4)	5.9(2)
D2_2	0.3067(4)	0.5309(5)	0.306(2)	0.674(4)	5.9(2)
D3_2	0.3578(7)	0.5186(7)	0.3385(14)	0.674(4)	5.9(2)
N1_3	0.4214(6)	0.6039(6)	0.717(3)	0.181(3)	2.0(5)
D1_3	0.4553(9)	0.621(3)	0.815(4)	0.181(3)	2.0(5)
D2_3	0.3899(12)	0.5733(15)	0.804(4)	0.181(3)	2.0(5)
D3_3	0.430(3)	0.5839(18)	0.600(7)	0.181(3)	2.0(5)
N1_4	0.393(3)	0.716(3)	–0.015(7)	0.046(3)	2.0(2)
D1_4	0.396(12)	0.692(8)	0.104(13)	0.046(3)	2.0(2)
D2_4	0.396(8)	0.750(5)	0.059(18)	0.046(3)	2.0(2)
D3_4	0.352(4)	0.693(9)	–0.07(3)	0.046(3)	2.0(2)

Table S7: Assignments of vibrational modes and molecular motions of adsorbed NH_3 and the framework.

Wavenumber (cm^{-1})	Assignment
1630 – 1637	HNH degenerate deformation of coordinated and physisorbed NH_3
1290 – 1299, 1320 – 1329, 1357 – 1359, 1442 – 1447	HC in and out of plane wagging
1172 – 1186, 1240 – 1257	HN symmetric deformation of coordinated NH_3 and CH_2 in plane wagging of cyclohexane ring
1058 – 1063	HN wagging of physisorbed NH_3
781 – 807	HC in plane wagging of cyclohexane ring
751 – 763	HC wagging of NCH_2P group
683, 725, 743 – 747	HN rocking of coordinated NH_3
656 – 660	HC wagging
568 – 570, 583 – 585 , 600 – 604	HN rocking of coordinated NH_3
536 – 548	HN wagging of coordinated NH_3
462	HN wagging of physisorbed NH_3
451 – 453	CH_2 wagging (cyclohexane ring) and HN wagging of physisorbed NH_3
408	Rotation of coordinated NH_3
380 – 398	Rotational motion of CH_2
354	Ni – NH_3 stretching mode
303 – 305, 310	Rotation of coordinated NH_3
230 – 238, 247, 254 – 257, 261, 281, 288	Rotation of physisorbed NH_3
215 – 217, 218 – 220	Rotation and liberational motion of physisorbed NH_3 and translation of coordinated NH_3
199 – 200	Translation of coordinated NH_3
155 – 159, 162 – 179	Liberational and translational motion of physisorbed NH_3
44 – 60, 90 – 91, 105, 121 – 124, 128, 132 – 137	Translation of physisorbed NH_3

Table S8: Reported static NH_3 uptake of selected state-of-the-art MOF materials.

Material	Uptake (mmol g^{-1})	Conditions	Stability	Ref.
STA-12(Ni)	4.76	273 K, 100 µbar	Reversible for more than 90 cycles	This work
	4.42	298 K, 100 µbar		
	15.0	273 K, 1 bar		
	12.7	298 K, 1 bar		
Ni_acryl_TMA	23.5	298 K, 1 bar	Reversible for 5 cycles	37
Ni_acryl_TGA	17.4			
Ni_acrylate	13.1			
Ni_2Cl_2[BTDD]	11.7			
Mg_2(dobpdc)	8.25	298 K, 570 µbar	Reduction in uptake after 1st cycle	38
	23.9	298 K, 1 bar		
Ni_2(dobpdc)	5.16	298 K, 580 µbar		
	20.82	298 K,		
Zn_2(dobpdc)	4.98	298 K, 420 µbar		
	15.24	298 K, 1 bar		
Mn_2(dobpdc)	4.77	298 K, 360 µbar		
	13.26	298 K, 1 bar		
Co_2(dobpdc)	4.72	298 K, 540 µbar		
	13.34	298 K, 1 bar		
MFM-300(Al)	13.0	298 K, 1 bar	Reversible for 50 cycles	39
MFM-300(V^{III})	15.6	273 K, 1 bar	Reversible for 20 cycles	40
MFM-300(V^{IV})	17.3			
MFM-300(Fe)	15.6			
MFM-300(Cr)	14.0			
MFM-300(In)	12.0	293 K, 1 bar	Loss of crystallinity	
MFM-300(Sc)	19.5	273 K, 1 bar	Reversible for 90 cycles	41
MFM-300(Ti)	23.4	273 K, 1 bar	Reversible for 25 cycles	42
MFM-303(Al)	9.9	273 K, 1 bar	Reversible for 30 cycles	43
Cu_2Cl_2BBTA	19.8	298 K, 1 bar	Loss of crystallinity	44
Co_2Cl_2BBTA	17.9		Reversible for 3 cycles	
Ni_2Cl_2BBTA	14.7		Reversible for 3 cycles	
Mn_2Cl_2BTDD	15.5	298 K, 1 bar	Loss of crystallinity	45
Ni_2Cl_2BTDD	12.0		Reversible for 3 cycles	
Co_2Cl_2BTDD	12.0		Loss of crystallinity	
Ga-PMOF	10.5	298 K, 1 bar	Loss of crystallinity	46
In-MOF	9.4		Loss of crystallinity	
Al-PMOF	7.7		Reversible for 2 cycles	
NU-1000-Cl-120	7.8	298 K, 1 bar	Reversible for 3 cycles	47
NU-1000-Cl-300	7.1			
NU-1000-F-120	7.8	298 K, 1 bar	Reversible for 3 cycles	48
NU-1000-F-60	7.6			
Fe-MIL-101-SO_3H	17.8	298 K, 1 bar	Not discussed	49
UiO-66-defect	11.8	273 K, 1 bar	Reversible for 15 cycles	50
UiO-66-Cu^{I}	12.6			

UiO-66-Cu^{II}	16.9			
UiO-67	8.4	298 K, 1 bar	Loss of crystallinity after 1 cycle	51
Cu(cyhdc)	18.7	273 K, 1 bar	Reversible phase-change after NH_3 sorption	52
MFM-100	19.8	273 K, 1 bar	Phase change/loss of crystallinity	53
MFM-101	21.9			
MFM-102	20.4			
MFM-126	15.9			
MFM-127	21.5			
MFM-190(F)	16.8			
MFM-170	17.2			
Cu-MOP-1a	14.2			

References

1. Miller, S. R.; Pearce, G. M.; Wright, P. A.; Bonino, F.; Chavan, S.; Bordiga, S.; Margiolaki, I.; Guillou, N.; Férey, G.; Bourrelly, S.; Llewellyn, P. L. Structural Transformations and Adsorption of Fuel-Related Gases of a Structurally Responsive Nickel Phosphonate Metal−Organic Framework, Ni-STA-12. *J. Am. Chem. Soc.* **2008**, *130*, 15967–15981.
2. Wharmby, M. T.; Pearce, G. M.; Mowat, J. P. S.; Griffin, J. M.; Ashbrook, S. E.; Wright, P. A.; Schilling, L.-H.; Lieb, A.; Stock, N.; Chavan, S.; Bordiga, S.; Garcia, E.; Pirngruber, G. D.; Vreeke, M.; Gora, L. Synthesis and Crystal Chemistry of the STA-12 Family of Metal N,N′-Piperazinebis(methylenephosphonate)s and Applications of STA-12(Ni) in the Separation of Gases. *Microporous Mesoporous Mater.* **2012**, *157*, 3–17
3. Wharmby, M. T.; Mowat, J. P. S.; Thompson, S. P.; Wright, P. A. Extending the Pore Size of Crystalline Metal Phosphonates toward the Mesoporous Regime by Isoreticular Synthesis. *J. Am. Chem. Soc.* **2011**, *133*, 1266–1269.
4. Hou, X.; Sun, J.; Lian, M.; Peng, Y.; Jiang, D.; Xu, M.; Li, B.; Xu, Q. Emerging Synthetic Methods and Applications of MOF-Based Gels in Supercapacitors, Water Treatment, Catalysis, Adsorption, and Energy Storage. *Macromol. Mater. Eng.* **2023**, *308*, 2200469.
5. Zhuang, Z.; Mai, Z.; Wang, T.; Liu, D. Strategies for Conversion between Metal–Organic Frameworks and Gels. *Coord. Chem. Rev.* **2020**, *421*, 213461. DOI: 10.1016/j.ccr.2020.213461.
6. Hou, J.; Sapnik, A. F.; Bennett, T. D. Metal–Organic Framework Gels and Monoliths. *Chem. Sci.* **2020**, *11*, 310–323.
7. Livage, J.; Henry, M.; Sanchez, C. Sol-Gel Chemistry of Transition Metal Oxides. *Prog. Solid State Chem.* **1988**, *18*, 259–341.
8. Chapon, L. C.; Manuel, P.; Radaelli, P. G.; Benson, C.; Perrott, L.; Ansell, S.; Rhodes, N. J.; Raspino, D.; Duxbury, D.; Spill, E.; Norris, J. WISH: The New Powder and Single Crystal Magnetic Diffractometer on the Second Target Station. *Neutron News* **2011**, *22*, 22–25.
9. Coelho, A. TOPAS and TOPAS-Academic: An Optimization Program Integrating Computer Algebra and Crystallographic Objects Written in C++. *J. Appl. Crystallogr.* **2018**, *51*, 210–218.
10. Stephens, P. Phenomenological Model of Anisotropic Peak Broadening in Powder Diffraction. *J. Appl. Crystallogr.* **1999**, *32*, 281–289.
11. Kresse, G.; Furthmüller, J. Efficient Iterative Schemes for Ab Initio Total-Energy Calculations Using a Plane-Wave Basis Set. *Phys. Rev. B* **1996**, *54*, 11169–11186.
12. Kresse, G.; Joubert, D. From Ultrasoft Pseudopotentials to the Projector Augmented-Wave Method. *Phys. Rev. B* 1999, *59*, 1758–1775.
13. Blöchl, P. E. Projector Augmented-Wave Method. *Phys. Rev. B* **1994**, *50*, 17953–17979.
14. Perdew, J. P.; Burke, K.; Ernzerhof, M. Generalized Gradient Approximation Made Simple. *Phys. Rev. Lett.* **1996**, *77*, 3865–3868.

15. Klimeš, J.; Bowler, D. R.; Michaelides, A. Chemical Accuracy for the van der Waals Density Functional. *J. Phys. Condens. Matter* **2010**, *22*, 022201.
16. Wang, T.; Maxisch, T.; Ceder, G. Oxidation Energies of Transition Metal Oxides within the GGA+U Framework. *Phys. Rev. B* **2006**, *73* (19), 195107. DOI: 10.1103/PhysRevB.73.195107.
17. Togo, A.; Tanaka, I. First Principles Phonon Calculations in Materials Science. *Scripta Mater.* **2015**, *108*, 1–5.
18. Cheng, Y. Q.; Daemen, L. L.; Kolesnikov, A. I.; Ramirez-Cuesta, A. J. Simulation of Inelastic Neutron Scattering Spectra Using OCLIMAX. *J. Chem. Theory Comput.* **2019**, *15* (3), 1974–1982.
19. Dubbeldam, D.; Calero, S.; Ellis, D. E.; Snurr, R. Q. RASPA: Molecular Simulation Software for Adsorption and Diffusion in Flexible Nanoporous Materials. *Mol. Simul.* **2016**, *42* (2), 81–101.
20. Herrera, L. F.; Do, D. D.; Birkett, G. R. Comparative Simulation Study of Nitrogen and Ammonia Adsorption on Graphitized and Nongraphitized Carbon Blacks. *J. Colloid Interface Sci.* **2008**, *320*, 415–422.
21. Mayo, S. L.; Olafson, B. D.; Goddard, W. A. DREIDING: A Generic Force Field for Molecular Simulations. *J. Phys. Chem.* **1990**, *94*, 8897–8909.
22. Rappe, A. K.; Casewit, C. J.; Colwell, K. S.; Goddard, W. A., III; Skiff, W. M. UFF, A Full Periodic Table Force Field for Molecular Mechanics and Molecular Dynamics Simulations. *J. Am. Chem. Soc.* **1992**, *114*, 10024–10035.
23. Kancharlapalli, S.; Gopalan, A.; Haranczyk, M.; Snurr, R. Q. Fast and Accurate Machine Learning Strategy for Calculating Partial Atomic Charges in Metal–Organic Frameworks. *J. Chem. Theory Comput.* **2021**, *17*, 3052–3064.
24. Vlugt, T. J. H.; García-Pérez, E.; Dubbeldam, D.; Ban, S.; Calero, S. Computing the Heat of Adsorption Using Molecular Simulations: The Effect of Strong Coulombic Interactions. *J. Chem. Theory Comput.* **2008**, *4*, 1107–1118.
25. Finsy, V.; Calero, S.; García-Pérez, E.; Merkling, P. J.; Vedts, G.; De Vos, D. E.; Barona, G. V.; Denayer, J. F. M. Low-Coverage Adsorption Properties of the Metal–Organic Framework MIL-47 Studied by Pulse Chromatography and Monte Carlo Simulations. *Phys. Chem. Chem. Phys.* **2009**, *11*, 3515–3521.
26. Rogacka, J.; Firlej, L.; Kuchta, B. Modeling of Low Temperature Adsorption of Hydrogen in Carbon Nanopores. *J. Mol. Model.* **2017**, *23*, 20.
27. Rogacka, J.; Formalik, F.; Triguero, A. L.; Firlej, L.; Kuchta, B.; Calero, S. Intermediate States Approach for Adsorption Studies in Flexible Metal–Organic Frameworks. *Phys. Chem. Chem. Phys.* **2019**, *21*, 3294–3303.

28. Rogacka, J.; Seremak, A.; Luna-Triguero, A.; Formalik, F.; Matito-Martos, I.; Firlej, L.; Calero, S.; Kuchta, B. High-Throughput Screening of Metal–Organic Frameworks for CO□ and CH□ Separation in the Presence of Water. *Chem. Eng. J.* **2021**, *403*, 126392.

29. Macrae, C. F.; Sovago, I.; Cottrell, S. J.; Galek, P. T. A.; McCabe, P.; Pidcock, E.; Platings, M.; Shields, G. P.; Stevens, J. S.; Towler, M.; Wood, P. A. Mercury 4.0: From Visualization to Analysis, Design, and Prediction. *J. Appl. Crystallogr.* **2020**, *53*, 226–235.

30. Spek, A. Structure Validation in Chemical Crystallography. *Acta Crystallogr., Sect. D* **2009**, *65*, 148–155.

31. Dolomanov, O. V.; Bourhis, L. J.; Gildea, R. J.; Howard, J. A. K.; Puschmann, H. OLEX2: A Complete Structure Solution, Refinement, and Analysis Program. *J. Appl. Crystallogr.* **2009**, *42*, 339–341.

32. Hermer, N.; Stock, N. The New Triazine-Based Porous Copper Phosphonate [Cu□(PPT)(H□O)□]·10H□O. *Dalton Trans.* **2015**, *44*, 3720–3723.

33. Hermer, N.; Reinsch, H.; Mayer, P.; Stock, N. Synthesis and Characterisation of the Porous Zinc Phosphonate [Zn□(H□PPB)(H□O)□]·xH□O. *CrystEngComm* **2016**, *18*, 8147–8150.

34. Reichenau, T. M.; Steinke, F.; Wharmby, M. T.; Näther, C.; Engesser, T. A.; Stock, N. Targeted Synthesis of a Highly Stable Aluminium Phosphonate Metal–Organic Framework Showing Reversible HCl Adsorption. *Angew. Chem. Int. Ed.* **2023**, *62*, e202303561.

35. Medikonda, P.; Pilli, R.; Sastri, C. V.; Gumma, S. Adsorption of Gases on Small–Pore Aluminum Bisphosphonate MOF MIL–91(Al). *J. Chem. Sci.* **2021**, *133*, 100.

36. Taylor, J. M.; Vaidhyanathan, R.; Iremonger, S. S.; Shimizu, G. K. H. Enhancing Water Stability of Metal–Organic Frameworks via Phosphonate Monoester Linkers. *J. Am. Chem. Soc.* **2012**, *134*, 14338–14340.

37. Kim, D. W.; Kang, D. W.; Kang, M.; Choi, D. S.; Yun, H.; Kim, S. Y.; Lee, S. M.; Lee, J.-H.; Hong, C. S. High Gravimetric and Volumetric Ammonia Capacities in Robust Metal–Organic Frameworks Prepared via Double Postsynthetic Modification. *J. Am. Chem. Soc.* **2022**, *144*, 9672–9683.

38. Kim, D. W.; Kang, D. W.; Kang, M.; Lee, J.-H.; Choe, J. H.; Chae, Y. S.; Choi, D. S.; Yun, H.; Hong, C. S. High Ammonia Uptake of a Metal–Organic Framework Adsorbent in a Wide Pressure Range. *Angew. Chem., Int. Ed.* **2020**, *59*, 22531-22536.

39. Godfrey, H. G. W.; da Silva, I.; Briggs, L.; Carter, J. H.; Morris, C. G.; Savage, M.; Easun, T. L.; Manuel, P.; Murray, C. A.; Tang, C. C.; Frogley, M. D.; Cinque, G.; Yang, S.; Schröder, M. Ammonia Storage by Reversible Host–Guest Site Exchange in a Robust Metal–Organic Framework. *Angew. Chem., Int. Ed.* **2018**, *57*, 14778-14781.

40. Han, X.; Lu, W.; Chen, Y.; da Silva, I.; Li, J.; Lin, L.; Li, W.; Sheveleva, A. M.; Godfrey, H. G. W.; Lu, Z.; Tuna, F.; McInnes, E. J. L.; Cheng, Y.; Daemen, L. L.; McCormick McPherson, L. J.; Teat, S. J.; Frogley, M. D.; Rudić, S.; Manuel, P.; Ramirez-Cuesta, A. J.; Yang, S.; Schröder,

M. High Ammonia Adsorption in MFM-300 Materials: Dynamics and Charge Transfer in Host–Guest Binding. *J. Am. Chem. Soc.* **2021**, *143*, 3153–3161.

41. Guo, L.; Han, X.; Ma, Y.; Li, J.; Lu, W.; Li, W.; Lee, D.; da Silva, I.; Cheng, Y.; Rudić, S.; Manuel, P.; Frogley, M. D.; Ramirez-Cuesta, A. J.; Schröder, M.; Yang, S. High Capacity Ammonia Adsorption in a Robust Metal–Organic Framework Mediated by Reversible Host–Guest Interactions. *Chem. Commun.* **2020**, *58*, 5753-5756.
42. Zeng, X.; Li, J.; He, M.; Lu, W.; Crawshaw, D.; Guo, L.; Ma, Y.; Kippax-Jones, M.; Cheng, Y.; Manuel, P.; Rudić, S.; Frogley, M. D.; Schröder, M.; Yang, S. High Adsorption of Ammonia in a Titanium-Based Metal–Organic Framework. *Chem. Commun.* **2024**, *60*, 5912–5915.
43. Marsh, C.; Han, X.; Li, J.; Lu, Z.; Argent, S. P.; da Silva, I.; Cheng, Y.; Daemen, L. L.; Ramirez-Cuesta, A. J.; Thompson, S. P.; Blake, A. J.; Yang, S.; Schröder, M. Exceptional Packing Density of Ammonia in a Dual-Functionalized Metal–Organic Framework. *J. Am. Chem. Soc.* **2021**, *143*, 6586–6592.
44. Rieth, A. J.; Dincă, M. Controlled Gas Uptake in Metal–Organic Frameworks with Record Ammonia Sorption. *J. Am. Chem. Soc.* **2018**, *140*, 3461–3466.
45. Rieth, A. J.; Tulchinsky, Y.; Dincă, M. High and Reversible Ammonia Uptake in Mesoporous Azolate Metal–Organic Frameworks with Open Mn, Co, and Ni Sites. *J. Am. Chem. Soc.* **2016**, *138*, 9401–9404.
46. Moribe, S.; Chen, Z.; Alayoglu, S.; Syed, Z. H.; Islamoglu, T.; Farha, O. K. Ammonia Capture within Isoreticular Metal–Organic Frameworks with Rod Secondary Building Units. *ACS Mater. Lett.* **2019**, *1*, 476–480.
47. Liu, J.; Chen, Z.; Wang, R.; Alayoglu, S.; Islamoglu, T.; Lee, S.-J.; Sheridan, T. R.; Chen, H.; Snurr, R. Q.; Farha, O. K.; Hupp, J. T. Zirconium Metal–Organic Frameworks Integrating Chloride Ions for Ammonia Capture and/or Chemical Separation. *ACS Appl. Mater. Interfaces* **2021**, *13*, 22485–22494.
48. Liu, J.; Lu, Z.; Chen, Z.; Rimoldi, M.; Howarth, A. J.; Chen, H.; Alayoglu, S.; Snurr, R. Q.; Farha, O. K.; Hupp, J. T. Ammonia Capture within Zirconium Metal–Organic Frameworks: Reversible and Irreversible Uptake. *ACS Appl. Mater. Interfaces* **2021**, *13*, 20081–20093.
49. Van Humbeck, J. F.; McDonald, T. M.; Jing, X.; Wiers, B. M.; Zhu, G.; Long, J. R. Ammonia capture in porous organic polymers densely functionalized with Brønsted acid groups. *J. Am. Chem. Soc.* **2014**, *136*, 2432–2440.
50. Ma, Y.; Lu, W.; Han, X.; Chen, Y.; da Silva, I.; Lee, D.; Sheveleva, A. M.; Wang, Z.; Li, J.; Li, W.; Fan, M.; Xu, S.; Tuna, F.; McInnes, E. J. L.; Cheng, Y.; Rudić, S.; Manuel, P.; Frogley, M. D.; Ramirez-Cuesta, A. J.; Schröder, M.; Yang, S. Direct Observation of Ammonia Storage in UiO-66 Incorporating Cu(II) Binding Sites. *J. Am. Chem. Soc.* **2022**, *144*, 8624–8632.

51. Yoskamtorn, T.; Zhao, P.; Wu, X. P.; Purchase, K.; Orlandi, F.; Manuel, P.; Taylor, J.; Li, Y.; Day, S.; Ye, L.; Tang, C. C.; Responses of defect-rich Zr-based metal-organic frameworks toward NH_3 adsorption. *J. Am. Chem. Soc.* **2021**, *143*, 3205–3218.

52. Snyder, B. E. R.; Turkiewicz, A. B.; Furukawa, H.; Paley, M. V.; Velasquez, E. O.; Dods, M. N.; Long, J. R. A Ligand Insertion Mechanism for Cooperative NH□ Capture in Metal–Organic Frameworks. *Nature* **2023**, *613*, 287–291.

53. Lu, W.; Chen, Y.; Wang, Z.; Chen, J.; Ma, Y.; Li, W.; Li, J.; He, M.; Fan, M.; Sheveleva, A. M.; Tuna, F.; McInnes, E. J. L.; Frogley, M. D.; Chater, P. A.; Dejoie, C.; Schröder, M.; Yang, S.; Guo, L. High Ammonia Adsorption in Copper-Carboxylate Materials: Host-Guest Interactions and Crystalline-Amorphous-Crystalline Phase Transitions. *Chem. Commun.* **2024**, Accepted Manuscript.
